# Systematic review and meta-analysis of studies in which burrowing behaviour was assessed in rodent models of disease-associated persistent pain

**DOI:** 10.1097/j.pain.0000000000002632

**Published:** 2022-03-29

**Authors:** Xue Ying Zhang, Ahmed Barakat, Marta Diaz-delCastillo, Jan Vollert, Emily S. Sena, Anne-Marie Heegaard, Andrew S.C. Rice, Nadia Soliman

**Affiliations:** a Pain Research, Department of Surgery and Cancer, Faculty of Medicine, Imperial College London, London, United Kingdom; b Department of Drug Design and Pharmacology, Faculty of Health and Medical Sciences, University of Copenhagen, Copenhagen, Denmark; c Department of Pharmacology and Toxicology, Faculty of Pharmacy, Assiut University, Assiut, Egypt; d Division of Neurological Pain Research and Therapy, Department of Neurology, University Hospital of Schleswig-Holstein, Campus Kiel, Germany; e Department of Anaesthesiology, Intensive Care and Pain Medicine, University Hospital Muenster, Germany; f Neurophysiology, Mannheim Centre of Translational Neuroscience (MCTN), Medical Faculty Mannheim, Heidelberg University, Germany; g Centre for Clinical Brain Sciences, University of Edinburgh, Edinburgh, United Kingdom

**Keywords:** Burrowing, Pain, Systematic review, Meta-analysis, Rodents, Preclinical, Behavioural test, Animal models

## Abstract

Burrowing behaviour is used to assess pain-associated behaviour in laboratory rodents. To gain insight into how models of disease-associated persistent pain and analgesics affect burrowing behaviour, we performed a systematic review and meta-analysis of studies that assessed burrowing behaviour. A systematic search in March 2020 and update in September 2020 was conducted in 4 databases. Study design characteristics and experimental data were extracted, followed by a random-effects meta-analysis. We explored the association between burrowing and monofilament-induced limb withdrawal. Dose response relationship was investigated for some analgesics. Forty-five studies were included in the meta-analysis, in which 16 model types and 14 drug classes were used. Most experiments used rat (79%) and male (72%) animals. Somatic inflammation and trauma-induced neuropathy models were associated with reduced burrowing behaviour. Analgesics (nonsteroidal anti-inflammatory drug and gabapentinoids) attenuated burrowing deficits in these models. Reporting of measures to reduce risk of bias was unclear except for randomisation which was high. There was not a correlation (*R*^2^ = 0.1421) between burrowing and monofilament-induced limb withdrawal. Opioids, gabapentin, and naproxen showed reduced burrowing behaviour at high doses, whereas ibuprofen and celecoxib showed opposite trend. The findings indicate that burrowing could be used to assess pain-associated behaviour. We support the use of a portfolio of composite measures including spontaneous and stimulus-evoked tests. The information collected here could help in designing experiments involving burrowing assessment in models of disease-associated pain.

## Abstract 2

Supplemental Digital Content is Available in the Text.

## 1. Introduction

Chronic pain is a leading cause of disability and disease burden worldwide.^44,57^ Developing analgesics with better efficacy and safety profiles remains a high priority. Many novel analgesics with promising preclinical results failed to translate into the clinic.^4,38,43,60^ This raises concerns about the validity of animal pain research, particularly the clinical and ethological relevance of the models and whether outcome measures used are reflecting the clinical construct they claim to measure.

Stimulus-evoked limb withdrawal responses (eg, monofilaments test) are widely used as surrogate outcome measures to quantify nociception in rodents.^13^ However, these stimulus-evoked behavioural outcomes have limitations: first, they are only useful in assessing certain sensory phenotypes associated with gain of function, allodynia, and hyperalgesia.^43^ They cannot address spontaneous pain and pain in clinical phenotypes relating to sensory loss of function; hence, they do not fully reflect the construct (ie, pain) being measured. Second, they are prone to generating false positive or negative results. Rodents are prey species and can mask behaviours that make them appear weak or vulnerable during the stimulus-evoked paradigms. Rodents can also associate premature withdrawal with less stimulation and human interaction.^13^ Furthermore, these paradigms cannot distinguish analgesic effects from adverse effects such as sedation, and the subjective assessment of stimulus-evoked behaviours can potentially threaten a study's predictive validity further. Finally, they cannot provide information on how pain affects the emotional and physical function of an animal. To capture different aspects of pain and improve the validity of preclinical pain research, stimulus-evoked behavioural outcomes should be assessed in combination with other ethologically relevant outcome measures.

Using complex ethologically relevant behaviours as a form of non-evoked pain-related outcome measures has become increasingly popular in recent years.^1,34,36,47,61^ Ethologically relevant behaviours can provide insights into how an animal's physical wellbeing and its affective state can be affected by pain. These behaviours are not pain specific, and can be perturbed by various stress factors and disease conditions, so it is crucial to contextualise these ethologically relevant behaviours to pain. Researchers can achieve pain contextualisation by showing that changes in these behaviours are caused by disease models associated with pain and that the changes can be reversed by administering known analgesics.

Burrowing is an ethological behaviour observed in some rodent species.^3,10^ Rodents excavate underground holes and tunnels to construct habitation. In laboratory rodent strains, burrowing is also a highly motivated social behaviour with a self-rewarding component.^11^ Deficits in burrowing behaviour correlate with various perturbations, including pain, and are quantified by measuring the weight of substrate displaced from an artificial burrow. The risk of handling–induced stress-related false positive or negative results is mitigated as animals are left alone during the assessment. Reduced rodent burrowing behaviour has been observed in numerous disease models associated with persistent pain^1,5,18,45,49^ and has been validated in a prospective multicentre study.^59^ Studies have demonstrated that clinically used analgesics attenuated burrowing deficits caused by experimental persistent pain, supporting the predictive validity of the test.^18,27,29,45^ Furthermore, burrowing is an ethologically fundamental activity, particularly for rats, as deficits in such behaviour can negatively affect their chance of survival in the wild or “quality of life” under domestication.^40,42^ Studies have demonstrated that laboratory-bred rat strains also readily burrow when they are placed in a more naturalistic environment.^42,52^ Given laboratory-bred rodents spontaneously exhibit burrowing, this behaviour has good face validity and is considered comparable with the “activities of daily living” in humans. Therefore, measuring changes in burrowing behaviour could help to address the global impact of pain on rodents.

Finally, the wide usage of monofilaments tests in rodent pain research inspired us to assess the association between monofilament-evoked limb withdrawal and burrowing outcomes.

### 1.1. Aims and objectives

This systematic review aimed to (1) assess whether rodent burrowing behaviour is influenced by rodent models associated with persistent pain and analgesic drug interventions, (2) explore study design characteristics and assess their impact on burrowing outcomes, (3) perform a risk of bias assessment to evaluate studies' methodological quality, (4) identify the presence of publication bias and determine its direction and magnitude, and (5) assess the correlation between monofilament-evoked limb withdrawal and burrowing outcomes in the same cohort of animals.

## 2. Methods

The review protocol was registered on PROSPERO (CRD42020172320; full protocol: https://www.crd.york.ac.uk/prospero/display_record.php?RecordID=172320). The only protocol deviation is that the rationale for conducting power analysis was changed (see 2.6.5. Power analysis).

### 2.1. Search strategy

We systematically searched EMBASE using Ovid, PubMed, Scopus, and Web of Science on March 23, 2020, and September 29, 2020, with no restrictions on languages and date of publication. The full search strategy for each database is provided in Supplemental Digital Content 1 (available at http://links.lww.com/PAIN/B603).

Duplicates of retrieved studies were removed using EndNote. In addition, reference lists of eligible studies were manually searched to identify studies missed by the database search.

### 2.2. Eligibility criteria

#### 2.2.1. Inclusion criteria

(1) Population—in vivo rodent models of disease associated with persistent pain (ie, induced chemically, surgically, or genetically and developed over a period of an hour, weeks, or months).
(2) Intervention—any clinically approved or novel analgesics used to interfere with nociception.
(3) Comparison—a cohort of control animals.
  (i) For animal modelling experiments (ie, assessed effects of persistent pain-related disease models on rodent burrowing behaviour), a control population was defined as sham or naive. If sham and naive controls were not reported, the baseline measurements of the same animals before model induction were regarded as control.
  (ii) For studies that used transgenic rodents to study persistent pain, a wild-type control was required.
  (iii) For drug intervention experiments (ie, assessed effects of pharmacological interventions on rodent burrowing behaviour), a vehicle control was required.
(4) Outcome—burrowing outcomes.

For the meta-analysis, we required a study to report the following data: (1) the mean burrowing outcome, (2) its variance (ie, SD or SEM), and (3) the number of animals per group.

#### 2.2.2. Exclusion criteria

Non-rodent in vivo studies and studies that investigated acute nociception (ie, measure reflex withdrawal responses to mechanical and thermal stimulus in non–disease-induced animals) were excluded. Studies were also excluded if the burrowing outcome was not reported or there was not an appropriate control group. Studies that were not primary research articles were excluded.

### 2.3. Study selection

Screening of retrieved studies were completed on the Systematic Review Facility (SyRF)^2^ platform. Studies were screened against the inclusion criteria twice based on (1) titles and abstracts and (2) full texts by 2 independent reviewers (X.Y.Z. and A.B.). Discrepancies were resolved by a third independent reviewer (N.S.).

### 2.4. Data extraction

Data extraction was conducted concurrently to the full-text screening stage on SyRF by 2 independent reviewers (X.Y.Z. and A.B.).

#### 2.4.1. Data collection

Study-level data were extracted (Table 1), and studies that were eligible for meta-analysis had experimental data extracted (Table 2). The primary outcome of interest was any outcome metric that denoted burrowing behaviour. The secondary outcome of interest was monofilament-evoked limb withdrawal assessed in the same cohort of animals. Continuous data were extracted independent of the unit of measurement. Digital ruler software (WebPlotDigitizer) was used to manually extract graphically presented data. When multiple time points were reported, the time point of the maximum effect was extracted. If the type of variance (ie, SEM or SD) was not reported, it was characterised as SD (ie, to give the most conservative estimate). The most conservative estimate was extracted when sample size data were given as a range. When key information was unclear or not reported (ie, sample size and variance), the corresponding author was contacted. If the author did not respond or was unable to provide the information, the study was recorded as having missing data and was excluded from the meta-analysis.

**Table 1:** Study-level data extracted from each included study.

Study level
Bibliographic detail First author Year of publication Title
Reporting quality Reporting guidelines, such as the ARRIVE, were developed for the purpose to improve the reporting of animal research. The following items were extracted: Reference following a reporting guideline for in vivo experimentation Provide evidence of reporting in accordance with the chosen guideline
Abstract spin Spin is defined as intentional and unintentional reporting practices that mislead the readers by misinterpreting the true effects so that conclusions are perceived in a favourable light. We used the following criteria from the “Protocol of intervention to reduce spin in the abstract conclusion” (registered on Open Science Framework: https://osf.io/49r5c/) to assess abstract spin: Report information that is not supported by evidence or in accordance with the study results Report interpretation that is not consistent with the study design or results
Methodological citing Often experiments are conducted in line with previously reported protocols. We extracted the cited publication(s) for the burrowing assessment protocol from each study to assess variations in outcome measurement. In addition, we determined whether the burrowing outcome metric was the same as described by Andrews et al.^10^: weight displaced, which was the first study reported of using burrowing behaviour as a pain-related outcome measure, and whether authors provided justifications for using alternative burrowing outcome metrics.
Acclimatisation and animal husbandry Time period of acclimatisation to housing environment following transportation Housing environment Light–dark cycle Feeding regime
Burrowing assessment characteristics Experimental environment Characteristics of the artificial burrow tube (ie, colour, size, and material) Type of substrate Training or social facilitation Predefined baseline burrowing threshold as an inclusion criterion
Curated content Whether the study assessed the model effect on animal's motor activity Whether the study assessed the drug treatment effect on animal's motor activity Whether the analgesic dose was determined by conducting pilot experiments in naive animals of which the burrowing behaviours were not affected

**Table 2:** Experiment-level data extracted from each included study.

Experiment level
Animal Species Strain Sex Animal supplier Age (at the start of experiments) Weight
Disease model Method of model induction Perioperative analgesic(s) given before or during or after model induction
Intervention Dose Route of administration No. of administrations Time between drug treatment and model induction Time between drug treatment and burrowing assessment
Outcome measure assessment **Primary outcome: burrowing** Habituation time Assessment duration Direction of effect No. of trials and time separation between trials Time between the model induction and the first assessment Time between the model induction and the last assessment Time between the first treatment and the first assessment Sex of the investigator Presence of the investigator during assessments **Secondary outcome: limb withdrawal evoked by monofilaments** Habituation time Method of assessment (ie, duration, force, and area of application) Direction of effect No. of trials and time separation between trials Time between the model induction and the first assessment Time between the model induction and the last assessment Time between the first treatment and the first assessment
Numerical outcome data Unit Mean outcome Variance No. of animals per group No. of groups served by the control group

#### 2.4.2. Risk of bias assessment

Risk of bias was evaluated by using the adapted version of the CAMARADES checklist and SYRCLE Risk of Bias tool,^26,32^ which assessed the reporting of 6 methodological quality criteria: random group allocation, allocation concealment, blinding of outcome assessment, sample size calculation, predefined animal inclusion criteria, and animal exclusions. Reviewers stated whether each criterion was reported with a description of the method that the study used. A separate rating was given to each item according to the following criteria: low risk (accepted methods and were adequately described), high risk (inappropriate methods that did not efficiently mitigate bias), and unclear risk (the methodological quality criterion was not reported or details of methods were insufficiently reported). Reporting of potential conflict of interests and compliance of animal welfare regulations were also extracted but were not included in the overall risk of bias.

### 2.5. Reconciliation

After data extraction, reconciliation was performed by a third independent reviewer (M.D.-d.C. and A.-M.H.). For graphically presented data, the third reviewer calculated the standardised mean difference (SMD) effect sizes of individual comparisons for the 2 reviewers. When individual comparisons differed by <10%, the third reviewer took an average of the 2 means and variance measures. When they differed by >10%, the outcome data were required to be extracted by the third reviewer.

### 2.6. Data analyses

X.Y.Z. and A.B. conducted the meta-analysis by following the guidelines described by Vesterinen et al.^56^ Burrowing outcome data were first separated according to the analytic approach reported by the protocol of the original study: intention-to-treat (ITT) analysis (animal exclusion was applied before experiments) and per protocol analysis, where animal exclusion was applied after experiments (ie, during analysis). Burrowing outcome data were primarily analysed using the ITT approach (ie, animal exclusion during the training phase); we, therefore, focused on the interpretation of ITT data in this article. Per protocol burrowing data are available at https://osf.io/96hmw/. Burrowing data were further separated by the type of experiment (ie, animal modelling or intervention experiments). The number of independent cohort-level effect sizes (*k*) required for each meta-analysis is ≥10. When *k* is <10, a descriptive summary was presented. Subgroup analyses were conducted to investigate how study characteristics influences effect sizes. All analyses were conducted using R statistical packages: dmetar (version 0.0.9), meta (version 4.15.1), and metafor (version 2.4.0).

#### 2.6.1. Effect size calculation

An effect size was calculated for each individual comparison, which was defined as a cohort of animals receiving treatment vs a control group using the Hedges' *g* SMD method. The use of sham control data was prioritised over naive control and then baseline of the same animals during effect size calculations. All sample size was corrected by dividing the reported number of animals in the control group by the number of treatment groups it served to obtain a “true number of control animals.” Effect sizes were weighted using the inverse variance method to reflect the contribution of each comparison with the total effect estimate. When more than 1 outcome metric of the same behavioural outcome was reported from the same cohort of animals, a single nested effect size, which denotes a summary effect of the cohort, was calculated. Cohort-level effect sizes were pooled using a random-effects model as it considers within-study and between-study variances. The restricted maximum-likelihood method was used to estimate the variance of the distribution of true effect sizes.^55^ The Hartung–Knapp–Sidik–Jonkman method was also applied to adjust confidence intervals (CIs).^20,21,48^

#### 2.6.2. Heterogeneity

Heterogeneity was assessed by Cochran *Q* and *I*^2^ tests. A *P* value was calculated for *Q*, giving an indication of whether all cohort-level effect sizes shared a common effect size (*P* > 0.05) or not (*P* < 0.05). The *I*^2^ test calculates the proportion of total variance between studies that is due to true differences in effect sizes as opposed to chance. *I*^2^ values were interpreted according to the definition given by Higgins and Thompsons^24^: 0% to 25% indicates very low heterogeneity, 25% to 50% indicates low heterogeneity, 50% to 75% indicates moderate heterogeneity, and >75% indicates high heterogeneity.

#### 2.6.3. Subgroup analyses

Stratified meta-analyses for categorical variables were performed according to rodent species, strain, sex, model type, drug class, type of burrowing substrate, type of burrowing measurement, and methodological quality criteria. Multivariate meta-regressions were planned to identify other factors that influence the burrowing outcome and, however, were not possible because of the low *k* number from each variable.

#### 2.6.4. Publication bias

Funnel plots were generated to visually inspect plot asymmetry. Standardised mean differences were plotted against sample size–based precision estimates (1/√N).^62^ Egger's regression test provided a statistical assessment of the presence of publication bias. Trim-and-fill analysis attempted to correct funnel plot asymmetry by imputing the theoretically missing studies to enable a recalculation of the effect size.

#### 2.6.5. Power analysis

Power analysis was originally planned to compare the number of animals required for burrowing and monofilaments tests. However, because the 2 tests measure different pain-associated behaviours, we conducted a power analysis to illustrate how researchers can use our metadata. We performed a power calculation for the burrowing outcome in rats induced with complete Freund's adjuvant (CFA). The most conservative estimate of the 95% CI of the pooled effect size was used to calculate the number of animals required. Calculations were based on the 2-sample 2-sided *t* test, with 80% power and a significance level of 0.05 (G*Power version: 3.1.9.7).

#### 2.6.6. Sensitivity tests

Sensitivity analyses were conducted to ascertain the robustness of our findings and to investigate whether a single study or group of studies have skewed the analysis. The following tests were performed:

(1) Baujat plot
(2) Single study exclusion sensitivity
(3) Cumulative study exclusion sensitivity
(4) Excluding studies with high risk of bias

A sensitivity analysis based on “excluding studies that reported burrowing as a primary outcome measure from those reporting it as a secondary outcome measure” could not be performed because only 5 studies declared this information.

Several post hoc (not planned in the registered protocol) analyses were also conducted:

#### 2.6.7. Correlation of burrowing and mechanically evoked limb withdrawal behavioural outcomes

Cohort-level comparisons of trauma-induced neuropathy models that assessed both burrowing and limb withdrawal were used to investigate correlation. A line was fitted using the least square method with subsequent *R*^2^ calculation.

#### 2.6.8. Dose-response relationships

Logarithmic transformation of different reported analgesics doses (mg/kg) was plotted against SMD effect sizes. Dose-response relationships were investigated in disease modelled and naive or sham animals. To avoid the confounding effect of repeated administration, single administration cohort-level comparisons were used. A post hoc analysis was admitted (not planned in the protocol) to calculate the significance level using the unpaired 2-tailed *t* test for each cohort-level comparison using the extracted mean and standard error of the control and intervention groups.^30^

#### 2.6.9. Drug effect on naive animals

Subgroup analysis based on intervention class was conducted to assess the effect of drug interventions on burrowing behaviour in naive rats.

## 3. Results

### 3.1. Study selection

**Figure 1.**
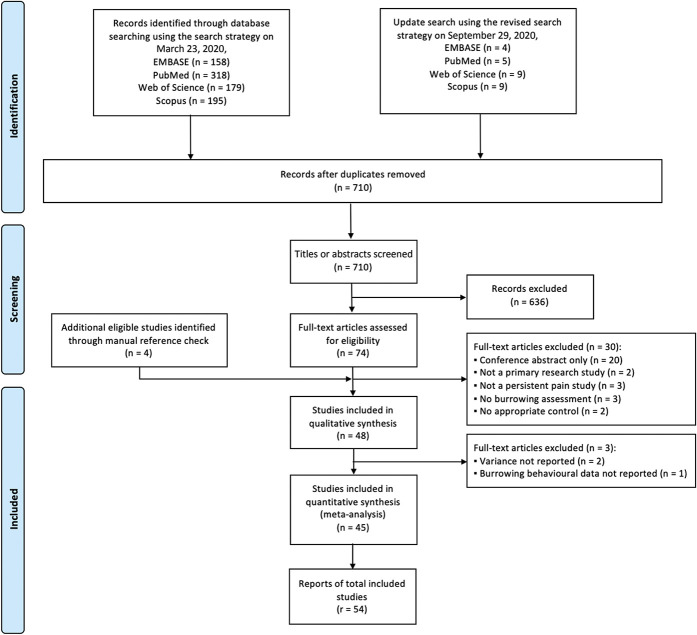
A flow diagram of publications identified through 2 separate systematic searches of 4 electronic databases (EMBASE, PubMed, Web of Science, and Scopus), which were conducted on March 23 and September 29, 2020. The diagram illustrates the number of records (n) at deduplication, screening, and study eligibility for both qualitative and quantitative analyses. *r* denotes the number of reports, which is defined as experimental data obtained by an individual research group within a study. Reported in accordance with the PRISMA 2020 guideline.^35^

A total of 710 publications were retrieved; of which, 74 studies were included after title and abstract screening. Full-text screening identified 48 studies for this review (Fig. 1). Of which, 3 studies were missing key information for meta-analysis. Among the 45 studies which were included in meta-analysis, there are 3 multicentre studies,^1,45,59^ and, thus, we extracted the data for each individual participant laboratory. A report is defined as experimental data obtained by an individual research group. In total, data from 54 reports were extracted.

### 3.2. Study characteristics

Meta-analysis of the 45 studies included a total of 3232 rodents (1590 in animal modelling and 1642 in intervention experiments). Burrowing behaviour was reported as investigated in 33 different rodent models associated with persistent pain. The models are listed according to the model type (16 model types) in Table 3; inflammation (27%, *k* = 53), arthropathy (23%, *k* = 44), and trauma-induced neuropathy (15%, *k* = 29) were the most frequently reported. Furthermore, 27 drug interventions were used to investigate the treatment effect on burrowing outcome in rodents modelled with persistent pain. The drugs are listed by their mechanism of action (14 drug classes) in Table 4; gabapentinoids (26%, *k* = 28), nonsteroidal anti-inflammatory drugs (NSAIDs) (25%, *k* = 27), and opioids (22%, *k* = 24) were the most frequently tested. Mice were used in 21% of experiments (*k* = 41), and 79% (*k* = 154) used rats. Moreover, 74% (*k* = 145) used male animals, 17% (*k* = 33) used female animals, 2% (*k* = 3) used mixed sexes, and 7% (*k* = 14) did not report the sex of the animals used.

**Table 3:** Summary of the model types used in animal modelling and drug intervention experiments of burrowing and monofilaments tests.

Model type	Model name	Burrowing experiments					Limb withdrawal evoked by monofilaments experiments				
		No. of studies	No. of reports	No. of *k*	No. of rats	No. of mice	No. of studies	No. of reports	No. of *k*	No. of rats	No. of mice
Arthropathy	Monosodium iodoacetate–induced osteoarthritis (intra-articular)	1	1	24	298	—	—	—	—	—	—
	Complete Freund's Adjuvant (intra-articular)	3	3	16	222	—	—	—	—	—	—
	Collagen-induced arthritis	1	1	3	48	—	1	1	3	40	—
	Medial meniscectomy–induced osteoarthritis	1	1	1	19	—	1	1	1	19	—
Neuropathy: antiretroviral-induced	Stavudine-induced neuropathy	1	1	1	15	—	—	—	—	—	—
Neuropathy: chemotherapy-induced	Bortezomib (cancer chemotherapy)-induced neuropathy	1	1	1	13	—	—	—	—	—	—
	Paclitaxel (cancer chemotherapy)-induced neuropathy	1	1	1	12	—	—	—	—	—	—
Neuropathy: diabetic-induced	Zucker diabetic fatty–induced neuropathy	1	1	11	203	—	1	1	1	29	—
	Streptozotocin-induced neuropathy	1	1	2	39	—	—	—	—	—	—
Neuropathy: trauma injury	Spared nerve injury	3	3	13	130	104	3	3	9	131	37
	Chronic constriction injury	2	3	11	204	—	1	1	1	37	—
	Tibial nerve transection	2	2	2	12	—	1	1	1	12	—
	L5 spinal nerve transection	1	1	1	21	—	1	1	1	21	—
	Partial sciatic nerve ligation	1	1	1	20	—	1	1	1	20	—
	Unilateral ligation of the infraorbital nerve	1	1	1	20	—	1	1	1	20	—
Spinal cord injury	Spinal cord contusion (thoracolumbar)	1	1	6	154	—	1	1	4	83	—
Nociplastic pain	Reserpine-induced fibromyalgia-like	1	1	4	—	44	1	1	3	—	48
Lumbar intervertebral disc degeneration	Surgical disruption of nucleus pulposus (L4/5, L5/6, and L6/S1)	1	1	1	—	39	1	1	1	—	39
Visceral inflammation	Dextran sulphate sodium–induced colitis	3	3	6	40	56	—	—	—	—	—
	Cerulein-induced pancreatitis	1	1	1	—	16	—	—	—	—	—
Mucositis	Fluorouracil- induced mucositis	1	1	4	72	—	—	—	—	—	—
	Irradiation-induced oral mucositis	1	1	1	—	16	—	—	—	—	—
Complex regional pain syndrome	Closed distal tibia fracture with casting	1	1	2	—	29	2	2	2	—	32
Dental injury	Surgically induced dental cavity	1	1	1	—	18	1	1	1	—	18
Cancer	Azoxymethane or dextran sulphate sodium–induced colitis or colitis-associated colorectal cancer	2	2	5	—	72	—	—	—	—	—
	Syngeneic orthotopic pancreatic tumour (6606PDA cancer cell line)	1	1	1	—	52	—	—	—	—	—
	Syngeneic breast cancer metastases to the bone (MRMT-1-Luc2 cancer cell line)	1	1	1	50	—	—	—	—	—	—
	Syngeneic breast cancer metastases to the bone (4T1-Luc2 cancer cell line)	1	1	1	—	33	—	—	—	—	—
Inflammation	Complete Freund's Adjuvant (intraplantar)	5	13	53	837	34	—	—	—	—	—
	Ultraviolet B and heat rekindling–induced inflammation	1	1	1	16	—	1	1	1	16	—
Migraine	Glyceryl trinitrate injection (intraperitoneal)	2	2	5	10	52	—	—	—	—	—
Procedure-associated pain	One-side sham embryo transfer (female) or 1-side sham vasectomy (male)	6	7	8	—	139	—	—	—	—	—
	Paclitaxel injection (intravenous or intraperitoneal)	2	2	4	73	—	2	2	4	66	—
Total				195	2528	704			35	494	174

The total number of studies and reports are not provided as summation will surpass the true total (45 studies and 54 reports) because of multiple disease models being investigated per study and reports.

*k*, independent cohort-level effect size.

**Table 4:** Summary of the drug classes used to assess the effect on burrowing and limb withdrawal behaviours in rodent disease model–associated persistent pain.

Drug class	Name	Burrowing experiments					Limb withdrawal evoked by monofilaments experiments				
		No. of studies	No. of reports	No. of *k*	No. of rats	No. of mice	No. of studies	No. of reports	No. of *k*	No. of rats	No. of mice
Bradykinin receptor antagonist	DALBK (B1 peptide antagonist)	1	1	1		11	1	1	1	—	16
	Icatibant (B2 peptide antagonist)	1	1	1		11	1	1	1	—	16
Antihyperglycaemic	Metformin	1	1	1		9	1	1	1	—	12
Dual amylin and calcitonin receptor agonist	KBP-042	1	1	1	13		1	1	1	13	—
Fatty acid amide hydrolase inhibitor	PF-04457845	1	1	3	33		—	—	—	—	—
GABA agonist	Diazepam	1	1	3	44		—	—	—	—	—
Gabapentinoid	Pregabalin	5	7	17	254	22	3	3	4	63	16
	Gabapentin	4	5	11	149	16	—	—	—	—	—
MicroRNA-21a-5p inhibitor	miRCURY LNA, Cat: #339203 YCO0070656, sequence: TCAGTCTGATAAGCT	1	1	1		16	—	—	—	—	—
Nerve growth factor antibody	Antinerve growth factor monoclonal antibody (Rinat)	1	1	1	16		—	—	—	—	—
	Tanezumab	1	1	1	23		—	—	—	—	—
Nonsteroidal anti-inflammatory drug	Ibuprofen	4	4	7	99		—	—	—	—	—
	Naproxen	3	3	6	77		1	1	1	13	—
	Celecoxib	2	2	6	77		—	—	—	—	—
	Carprofen	3	3	5		96	—	—	—	—	—
	Indomethacin	1	1	3	60		—	—	—	—	—
Opioid	Morphine	4	5	16	210		—	—	—	—	—
	Tramadol	3	3	7	78	16	—	—	—	—	—
	Buprenorphine	1	1	1		20	—	—	—	—	—
Sodium channel blocker	Carbamazepine	1	1	1	26		1	1	1	26	—
TRPV1 antagonist	SB366791	1	1	1	16		1	1	1	16	—
Unknown mechanism of action	Emu oil	2	2	4		58	—	—	—	—	—
	Docosahexaenoic acid	1	1	4	114		1	1	2	44	—
	Almond hull extract	1	1	2	20		—	—	—	—	—
	Almond blanched water	1	1	2	20		—	—	—	—	—
Combined therapy	Naproxen + KBP-042	1	1	1	22		1	1	1	13	—
	Tramadol + paracetamol	1	1	1		16	—	—	—	—	—
Total				108	1351	291			14	188	60

The total number of studies and reports are not provided as summation will surpass the true total (45 studies and 54 reports) because of multiple disease models being investigated per study and reports.

*k*, independent cohort-level effect sizes.

Of the 45 studies, 18 (40%) studies comprising a total of 668 rodents (419 in animal modelling and 249 in intervention experiments) reported conducting a monofilaments test as well as burrowing assessment. These studies included 16 different models encompassing 9 model types (Table 3). Trauma-induced neuropathy (51%, *k* = 18) was the most frequently assessed model type. Ten drug interventions (9 drug classes) were tested, where pregabalin was the most frequently assessed (29%; *k* = 4) (Table 4). Mouse experiments constituted 26% (*k* = 9), whereas rat experiments were 74% (*k* = 26). Moreover, 23% (*k* = 8) used female animals and 66% (*k* = 23) used male animals, whereas 11% (*k* = 4) did not report the sex.

### 3.3. Meta-analysis of burrowing outcomes

Species accounted for a significant proportion of heterogeneity (ITT data: animal modelling *Q* = 276.88, *df* 72, *P* < 0.0001; drug intervention *Q* = 224.56, *df* 106, *P* < 0.0001). Therefore, ITT animal modelling and intervention data of rats and mice were analysed separately. Further subgroup analyses were conducted to determine whether study design characteristics can influence effect sizes.

#### 3.3.1. Rats

##### 3.3.1.1. Burrowing behaviour was reduced by disease models associated with persistent pain

A total of 19 studies (28 reports), containing 51 cohort-level comparisons, 962 rats, and a N range from 6 to 50 with a median of 18, assessed the effects of 10 types of disease models associated with persistent pain on burrowing in 6 rat strains (Supplemental Digital Content 2, available at http://links.lww.com/PAIN/B603). Sprague–Dawley was the most reported strain (53%, *k* = 27). Male rats were used in 94% of experiments (*k* = 48), and female rats were used in 3% (*k* = 2). Three percent (*k* = 1) did not report the sex of the rats used. Experiments used different types of substrates: 59% (*k* = 30) used gravels, 35% used sand (*k* = 18), 4% used food pellets (*k* = 2), and 2% (*k* = 1) did not report the nature of substrates.

**Figure 2.**
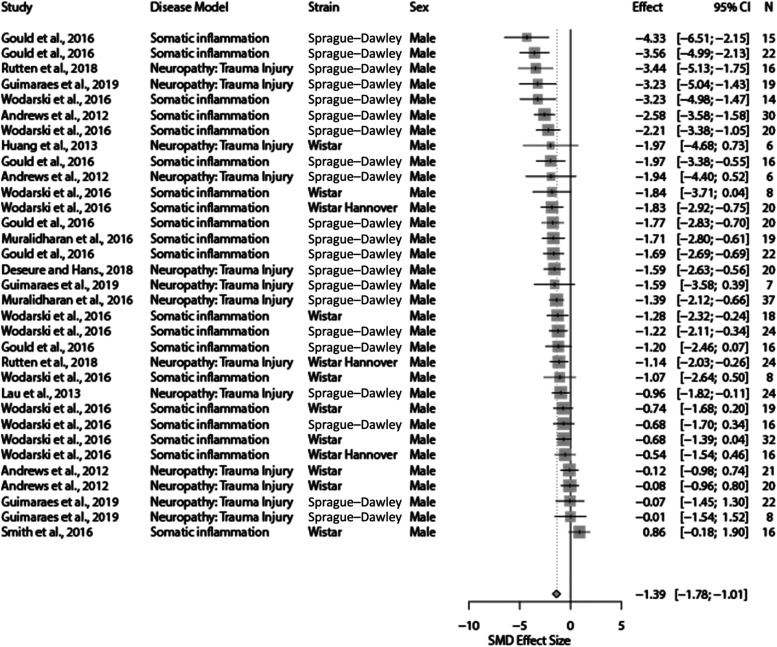
A summary forest plot of the 33 cohort-level comparisons which assessed the impact of modelling effects of somatic inflammation and trauma-induced neuropathy on burrowing in rats. For each comparison, an effect size was calculated using the Hedges' *g* SMD method. Effect sizes were pooled using the random effects model. The restricted maximum-likelihood method was used to estimate heterogeneity. The overall effect size is −1.39; *Q* = 90.84, *df* 32; *P* < 0.0001; *I*^2^ = 64.8%. The size of the square represents the weight, which reflects the contribution of each comparison with the pooled effect estimate. CI, confidence interval; N, number of animals; NR, not reported; SMD, standardised mean difference.

For meta-analysis, somatic inflammation and trauma-induced neuropathy were the only 2 eligible model types (*k* = 33 in total). Overall, the 2 disease models significantly reduced burrowing behaviour when compared with the control (SMD = −1.39 [95% CI −1.78 to −1.01]). Heterogeneity was moderate (*Q* = 90.84, *df* 32; *P* < 0.0001; *I*^2^ = 64.8%) (Fig. 2). Sensitivity analysis showed that removal of outliers did not affect the summary effect size significantly (SMD = −1.44 [95% CI −1.82 to −1.05]). In addition, removal of studies with high risk of bias did not significantly change the summary effect size (SMD = −1.34 [95% CI −1.71 to −0.96]). Details of the sensitivity tests are presented in the Supplemental Digital Content 3 (available at http://links.lww.com/PAIN/B603). All experiments measured the burrowing outcome as weight displaced.

###### 3.3.1.1.1. Effects of the animal model and study characteristics on the burrowing outcome

**Figure 3.**
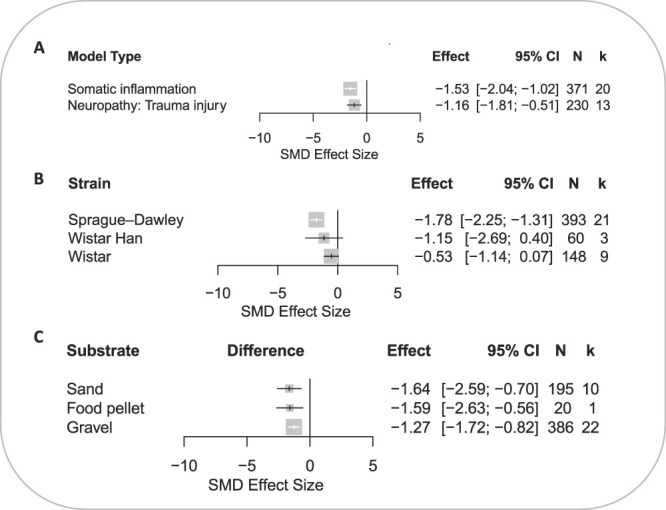
Forest plots of burrowing outcome in rats modelled with somatic inflammation and trauma-induced neuropathy: (A) model type, (B) rat strain, and (C) type of the burrowing substrate. The size of the square represents the weight. CI, confidence interval; *k*, number of cohort-level comparisons; N, number of animals; NR, not reported; SMD, standardised mean difference.

The model type did not account for a significant proportion of heterogeneity (*Q* = 0.92, *df* 1; *P* = 0.34) (Fig. 3A). Rats modelled with somatic inflammation had reduced burrowing outcome compared with rats with trauma-induced neuropathy (SMD = −1.53 vs −1.16).

The strain was accounted for a significant proportion of heterogeneity (*Q* = 13.07, *df* 2; *P* = 0.001) (Fig. 3B). Sprague–Dawley was the most reported strain with the largest significant reduction in burrowing behaviour (SMD = −1.78 [95% CI −2.25 to −1.31]). Analysis of studies reporting the use of Wistar and Wistar Hannover rats did not reveal a significant effect.

The type of substrate used in the burrowing assessment did not account for a significant proportion of heterogeneity (*Q* = 0.84 *df* 2; *P* = 0.66) (Fig. 3C). Of the 3 types of burrowing substrates that were reported, most experiments used gravel (67%, *k* = 33).

We could not ascertain the effect of sex on the burrowing outcome because all experiments used male rats.

**Figure 4.**
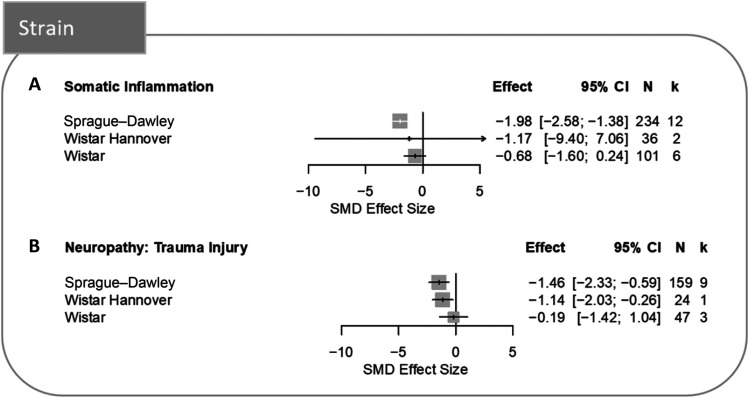
Forest plots of modelling effects of (A) somatic inflammation and (B) trauma-induced neuropathy on burrowing behaviour in different rat strains. The size of the square represents the weight. CI, confidence interval; *k*, number of cohort-level comparisons; N, number of animals; SMD, standardised mean difference.

Separate post hoc stratified meta-analyses were conducted according to the strain identified and revealed that somatic inflammation models only significantly reduced burrowing behaviour in Sprague–Dawley (Fig. 4A). However, trauma-induced neuropathy models significantly reduced burrowing behaviour in both Sprague–Dawley and Wistar Hannover rats (Fig. 4B).

##### 3.3.1.2. Burrowing deficits caused by disease models were attenuated by drug interventions

A total of 11 studies (12 reports), containing 89 cohort-level comparisons, 1351 rats, and a N range from 10 to 29 with a median of 14, assessed the effects of drug treatments from 11 drug classes on burrowing behaviour in 6 rat strains modelled with persistent pain (Supplemental Digital Content 4, available at http://links.lww.com/PAIN/B603). Gabapentinoid was the most reported drug class (28%, *k* = 25), and Sprague–Dawley was the most reported strain (54%, *k* = 48). Male rats were used in 81% of experiments (*k* = 72), and female animals were used in 8% (*k* = 7), whereas 11% (*k* = 10) did not report the sex of the rats used. Furthermore, experiments used 3 different types of substrates: 53% (*k* = 47) used sand, 46% (*k* = 41) used gravels, and 1% (*k* = 1) used food pellets.

**Figure 5.**
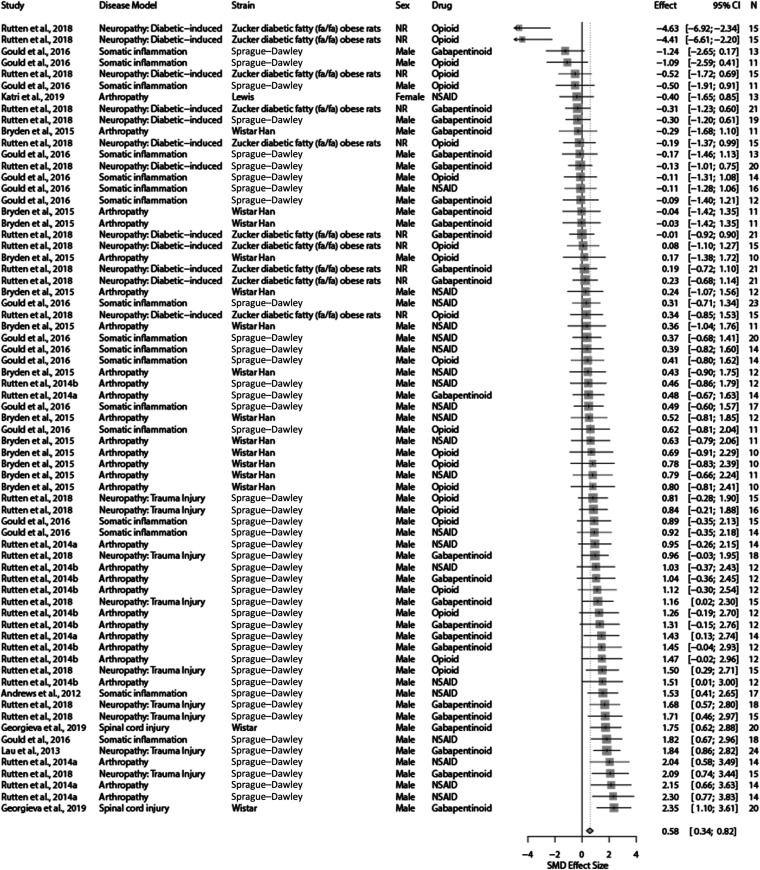
A summary forest plot of the 69 cohort-level comparisons of treatment effects of gabapentinoids, NSAIDs, and opioids on burrowing in rats modelled with persistent pain. For each comparison, an effect size was calculated using the Hedges' *g* SMD method. Effect sizes were pooled using the random effects model. The restricted maximum-likelihood method was used to estimate heterogeneity. The overall effect size is 0.58; *Q* = 147.15, *df* 68; *P* < 0.0001; *I*^2^ = 53.8%. The size of the square represents the weight, which reflects the contribution of each comparison with the pooled effect estimate. CI, confidence interval; N, number of animals; NR, not reported; NSAID, nonsteroidal anti-inflammatory drug; SMD, standardised mean difference.

For meta-analysis, enough cohort-level comparisons were reported only in drug intervention experiments using gabapentinoids, NSAIDs, and opioids (*k* = 69 in total). Gabapentinoids and NSAIDs significantly attenuated burrowing deficits caused by disease models compared with control, whereas opioids did not. The overall effect, for the 3 drug classes combined, was SMD = 0.58 [95% CI 0.34-0.82]. Heterogeneity was moderate (*Q* = 147.15, *df* 68; *P* < 0.0001; *I*^2^ = 53.8%) (Fig. 5). Sensitivity analysis showed that removal of outliers (studies with CIs that do not overlap with the CI of the summary effect size) did not affect the summary effect size significantly (SMD = 0.53 [95% CI 0.29-0.77]).^19^ In addition, removal of studies with high risk of bias did not significantly change the summary effect size (SMD = 0.49 [95% CI 0.21-0.77]). Details of the sensitivity tests are presented in the Supplemental Digital Content 5 file (available at http://links.lww.com/PAIN/B603). All experiments measured the burrowing outcome as weight displaced.

###### 3.3.1.2.1. Effects of animal model and study characteristics on the burrowing outcome

**Figure 6.**
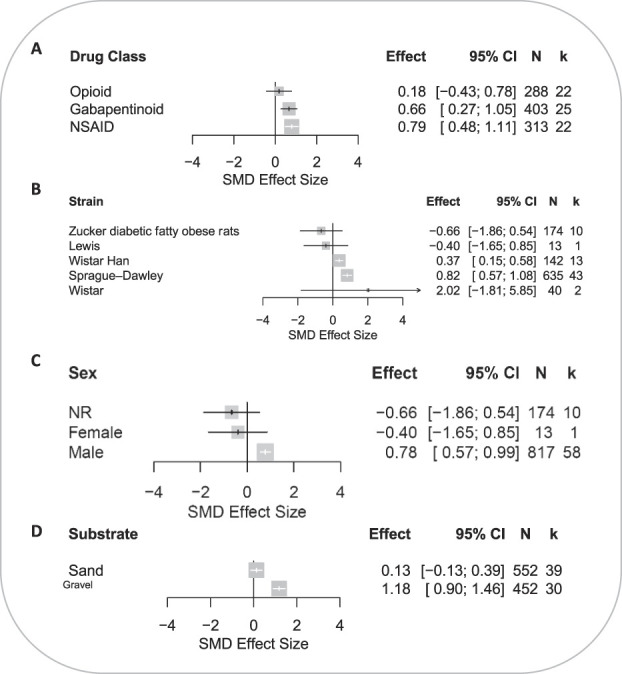
Forest plots of drug treatment effects on burrowing behaviour in rats modelled with persistent pain: (A) drug class, (B) rat strain, (C) sex, and (D) type of the burrowing substrate. The size of the square represents the weight. CI, confidence interval; *k*, number of cohort-level comparisons; N, number of animals; NR, not reported; SMD, standardised mean difference.

The drug class did not account for a significant proportion of the observed heterogeneity (*Q* = 39.08, *df* 2; *P* = 0.17) (Fig. 6A). Gabapentinoids and NSAIDs were associated with significant treatment effect; NSAIDs produced the largest significant attenuation in burrowing deficits compared with the control (SMD = 0.79 [95% CI 0.48-1.11]). Opioids did not attenuate burrowing deficits.

The strain accounted for a significant proportion of heterogeneity (*Q* = 39.08, *df* 4; *P* < 0.0001) (Fig. 6B). The most reported strain was Sprague–Dawley rats. Burrowing deficits of Wistar and Sprague–Dawley strains were significantly attenuated after drug treatments.

Sex accounted for a significant proportion of heterogeneity in rats (*Q* = 10.12, *df* 2; *P* = 0.006) (Fig. 6C). Burrowing deficits were significantly attenuated by drug treatments in male animals (SMD = 0.78 [95% CI 0.57-0.99]). However, it should be noted that the reporting of females is of a single cohort-level comparison and may hinder the generalisability of such findings.

The type of burrowing substrate accounted for a significant proportion of heterogeneity (*Q* = 31.79, *df* 1; *P* < 0.0001) (Fig. 6D). Burrowing assessments which used gravel as substrates were reported with significantly attenuated burrowing behaviour by drug treatments in rats modelled with persistent pain.

###### 3.3.1.2.2. Effect of drug class on burrowing outcomes

A post hoc stratified meta-analysis was conducted to assess the association of study characteristics and burrowing outcome in rats treated by the same drug class.

###### 3.3.1.2.3. Gabapentinoids

**Figure 7.**
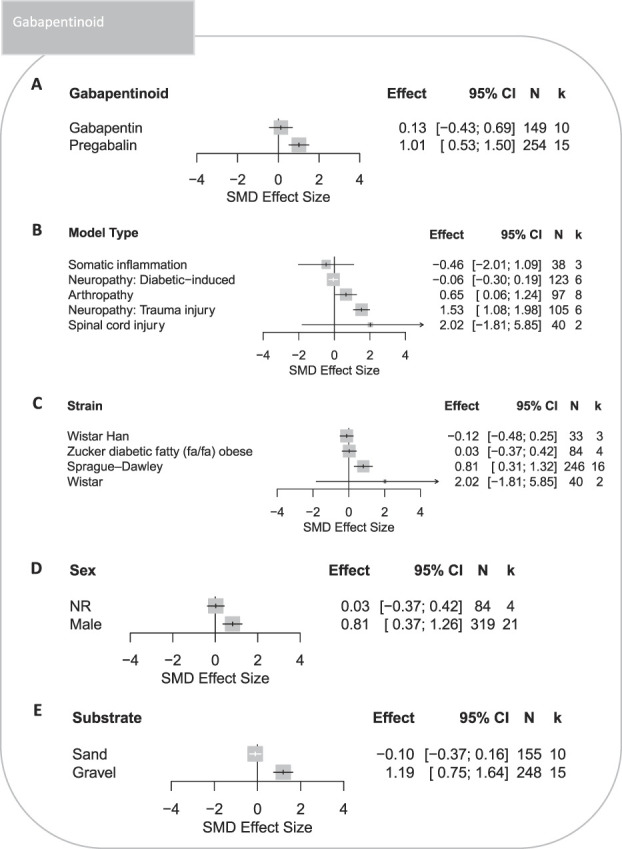
Forest plots of treatment effects of gabapentinoids on burrowing behaviour in rats modelled with persistent pain: (A) type of gabapentinoid, (B) model type, (C) strain, (D) sex, and (E) type of the burrowing substrate. The size of the square represents the weight. CI, confidence interval; *k*, number of cohort-level comparisons; N, number of animals; NR, not reported; SMD, standardised mean difference.

Pregabalin treatment significantly attenuated burrowing deficits in rats modelled with persistent pain (SMD = 1.01 [95% CI 0.53-1.50]), whereas treatment with gabapentin was ineffective (SMD = 0.13 [95% CI −0.43 to 0.69]) (Fig. 7A). Treatment effects of gabapentinoids were assessed in 5 model types, and only burrowing deficits associated with arthropathy and trauma-induced neuropathy were significantly attenuated (Fig. 7B). In addition, gabapentinoids were assessed in 4 rat strains; Sprague–Dawley was the most reported strain and also the only strain with a significant attenuation in burrowing deficits (Fig. 7C). Gabapentinoids significantly attenuated burrowing deficits of male rats (Fig. 7D); however, there were no data on the effect of gabapentinoids on female rats, so comparisons between different sexes could not be made. Experiments which used gravel substrates reported significant treatment effect (Fig. 7E).

###### 3.3.1.2.4. Nonsteroidal anti-inflammatory drugs

**Figure 8.**
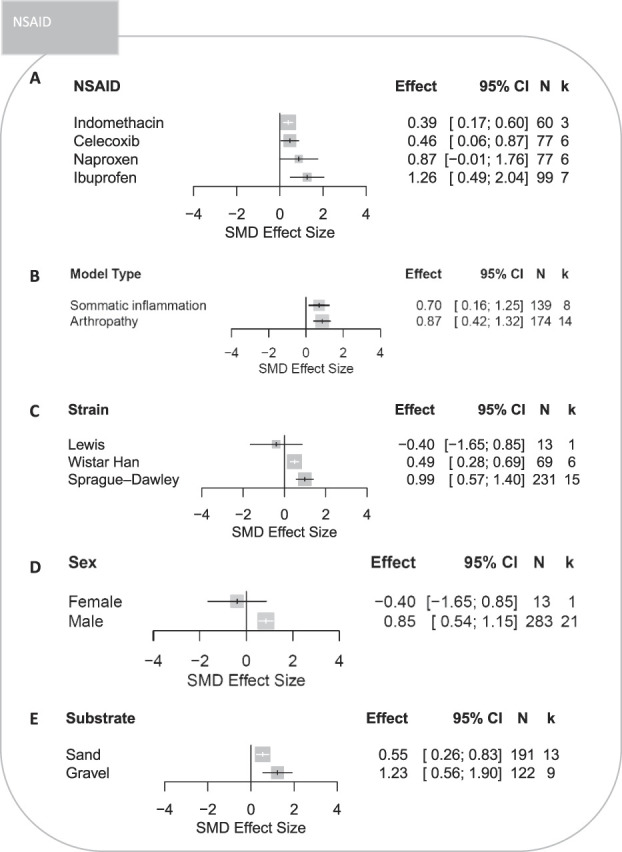
Forest plots of treatment effects of NSAIDs on burrowing behaviour in rats modelled with persistent pain: (A) type of NSAID, (B) model type, (C) strain, (D) sex, and (E) type of the burrowing substrate. The size of the square represents the weight. CI, confidence interval; *k*, number of cohort-level comparisons; N, number of animals; NR, not reported; NSAID, nonsteroidal anti-inflammatory drug; SMD, standardised mean difference.

Except naproxen, indomethacin, celecoxib, and ibuprofen significantly attenuated burrowing deficits in rats modelled with inflammation and arthropathy-induced persistent pain where ibuprofen showed the greatest efficacy (Figs. 8A and B). Significant treatment effects of NSAIDs were reported in Sprague–Dawley and Wistar Hannover strains (Fig. 8C). Burrowing deficits were significantly attenuated in male rats; however, the reporting of female rats is of a single cohort-level comparison (Fig. 8D). Greater treatment effects of NSAIDs were reported in experiments which used gravel substrates (Fig. 8E).

###### 3.3.1.2.5. Opioids

**Figure 9.**
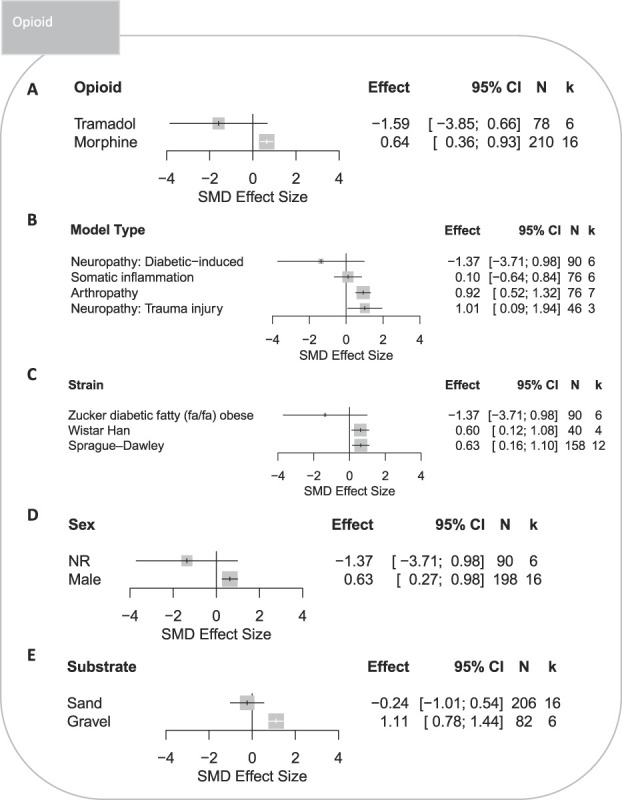
Forest plots of treatment effects of opioids on burrowing behaviour in rats modelled with persistent pain: (A) type of opioid, (B) model type, (C) strain, (D) sex, and (E) type of the burrowing substrate. The size of the square represents the weight. CI, confidence interval; *k*, number of cohort-level comparisons; N, number of animals; NR, not reported; SMD, standardised mean difference.

Tramadol did not significantly attenuate burrowing deficits. Morphine significantly attenuated burrowing deficits in rats (SMD = 0.64 [95% CI 0.36-0.93]) (Fig. 9A) modelled with arthropathy or trauma-induced neuropathy but was ineffective in models of somatic inflammation or diabetes-induced neuropathy (Fig. 9B). Opioids were assessed in 3 rat strains; Sprague–Dawley and Wistar Hannover strains were associated with significant attenuations in burrowing deficits (Fig. 9C). Opioids significantly attenuated burrowing deficits in male rats (Fig. 9D), but drug effect data of female rats were not available; therefore, comparisons between sexes could not be made. Experiments that used gravel substrates had a significant treatment effect (Fig. 9E).

#### 3.3.2. Mice

In this systematic review, we also assessed the effect of disease models associated with pain on burrowing behaviour of mice. However, a meta-analysis is restricted by the insufficient number of cohort-level comparisons; therefore, we present a descriptive summary of the mice burrowing data.

##### 3.3.2.1. Characteristics of modelling experiments in mice

In total, there were 12 studies (12 reports), comprising 22 cohort-level comparisons and 413 mice, that assessed the effects of 10 disease models associated with persistent pain on burrowing behaviour in 6 mouse strains (Supplemental Digital Content 6, available at http://links.lww.com/PAIN/B603). Trauma-induced neuropathy was the most assessed model type (23%, *k* = 5). C57BL/6 strain was used the most (45%, *k* = 10), and 32% (*k* = 7) of experiments did not report strain. Females were used in 55% of the experiments (*k* = 12), whereas males were used in 36% (*k* = 8), and 9% (*k* = 2) did not report the sex of the mice used. A total of 6 types of substrates were used; corncob beddings were used the most (54%, *k* = 12), and 9% of the experiments (*k* = 2) did not report the nature of substrates. Most experiments (95%, *k* = 21) measured the amount of substrate displaced.

##### 3.3.2.2. Characteristics of drug intervention experiments in mice

A total of 11 studies (11 reports), containing 18 cohort-level comparisons and 275 mice, assessed the effect of drug treatments from 7 drug classes on burrowing behaviour in 3 mouse strains modelled with persistent pain (Supplemental Digital Content 7, available at http://links.lww.com/PAIN/B603). Nonsteroidal anti-inflammatory drugs were the most assessed drug class (28%, *k* = 5), and procedure-associated pain was the most assessed model type (39%, *k* = 7). C57BL/6 strain was used the most (72%, *k* = 12), and 6% (*k* = 1) of experiments did not report strain. 66% of experiments (*k* = 12) used female animals, 22% (*k* = 4) used male animals, 6% (*k* = 1) used mixed sexes, and 6% (*k* = 1) did not report the sex of the mice used. 4 types of substrates were used; food pellets were used the most (61%, *k* = 11), and 11% (*k* = 2) did not report the nature of substrates. Most experiments (61%, *k* = 11) measured the weight displaced.

### 3.4. Risk of bias

**Figure 10.**
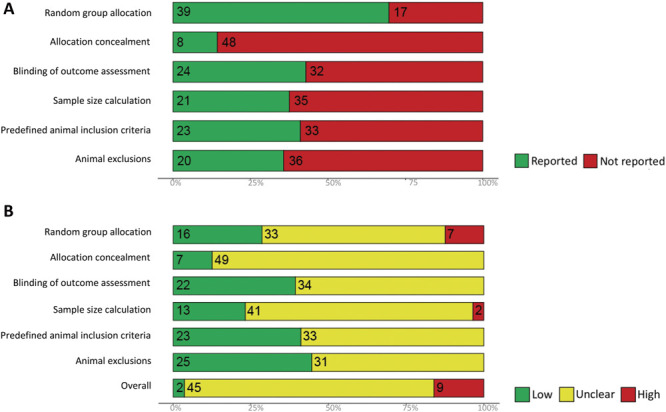
Summary plots showing the percentage of the 56 reports of the included studies that (A) reported the methodological quality criteria and (B) the corresponding risk of bias score given for each methodological quality criterion. Numbers shown within the bar plots indicate the number of reports. Reporting of a statement regarding potential conflict of interests and compliance with animal welfare regulations were extracted, but they were not part of the overall risk of bias.

The overall risk of bias of the 56 reports (from the 48 included studies for qualitative synthesis) is unclear. Only the reporting of random group allocation was high (70%). The reporting of other methodological quality criteria was low: 14% reported allocation concealment, 43% reported blinding of outcome assessment, 38% reported sample size calculation, 41% reported predefined animal inclusion criteria, and 36% reported animal exclusions (Fig. 10A). This contrasts with the high reporting of conflict of interest (86%, 48 reports) and of compliance of animal welfare regulations (98%, 55 reports). The specific methods and details used to mitigate bias were rarely reported; therefore, most are at an unclear risk of bias (Fig. 10B); however, 9 reports are at a high risk of bias: Authors of 7 reports explicitly stated that random group allocation was not performed for the purpose of matching basal burrowing activity in control and treatment groups, and authors of 2 reports stated sample size calculation was not performed. A “traffic light plot” presenting the risk of bias score for each report is available in Supplemental Digital Content 8 (available at http://links.lww.com/PAIN/B603).

#### 3.4.1. Impact of methodological quality criteria on burrowing effect sizes

**Figure 11.**
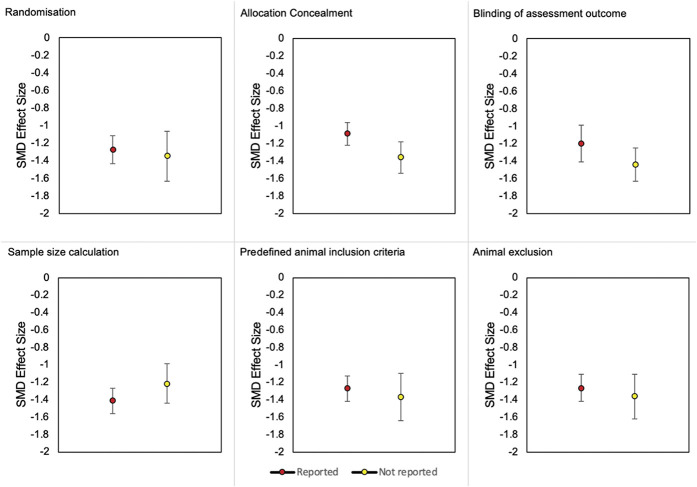
Burrowing effect sizes associated with the reporting of the 6 methodological quality criteria in modelling experiments of mice and rats. SMD, standardised mean difference.

To assess the impact of each criterion on burrowing effect sizes, ITT burrowing data of mice and rats were combined. In animal modelling experiments, reporting of the 6 methodological quality criteria did not account for a significant proportion of the observed heterogeneity (Fig. 11).

**Figure 12.**
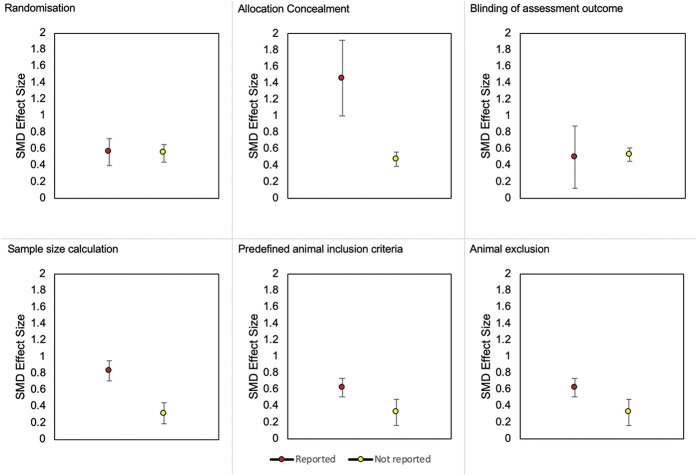
Burrowing effect sizes associated with the reporting of the 6 methodological quality criteria in interventions experiments of mice and rats. SMD, standardised mean difference.

In intervention experiments, reporting of allocation concealment and sample size calculation accounted for a significant proportion of the observed heterogeneity (Fig. 12). Larger effect sizes were observed in experiments that reported allocation concealment (SMD = 1.46 vs SMD = 0.48, *Q* = 6.75, *df* 1, *P* = 0.009) and sample size calculations (SMD = 0.83 vs SMD = 0.32, *Q* = 8.95, *df* 1, *P* = 0.003). It is noteworthy that the prevalence of reporting allocation concealment was low (*k* = 7 reported), which may limit our ability to accurately determine its influence on the burrowing outcome. The prevalence of reporting sample size calculation was similar between reported and not reported (*k* = 50 vs *k* = 57 comparisons).

### 3.5. Publication bias

#### 3.5.1. Animal modelling experiments

**Figure 13.**
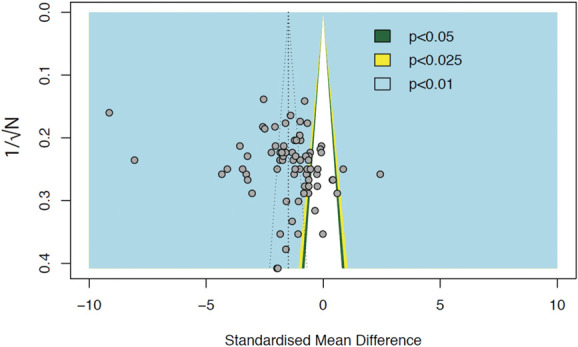
Assessment of publication bias in modelling experiments of rats and mice. Visual inspection of the funnel plot does not suggest asymmetry.

The overall effect size when combining modelling data of rats and mice (*k* = 73) is −1.49 (95% CI −1.88 to −1.10). Egger's regression test was insignificant (*P* = 0.05), suggesting no funnel plot asymmetry (Fig. 13), therefore, does not indicate publication bias. Most animal modelling experiments were reported with significantly reduced burrowing behaviour (plotted in the coloured backgrounds on the left-hand side). A few experiments were reported with insignificant effects (plotted in the central white background). Trim-and-fill analysis did not impute theoretically missing experiments, consistent with the absence of publication bias.

#### 3.5.2. Intervention experiments

**Figure 14.**
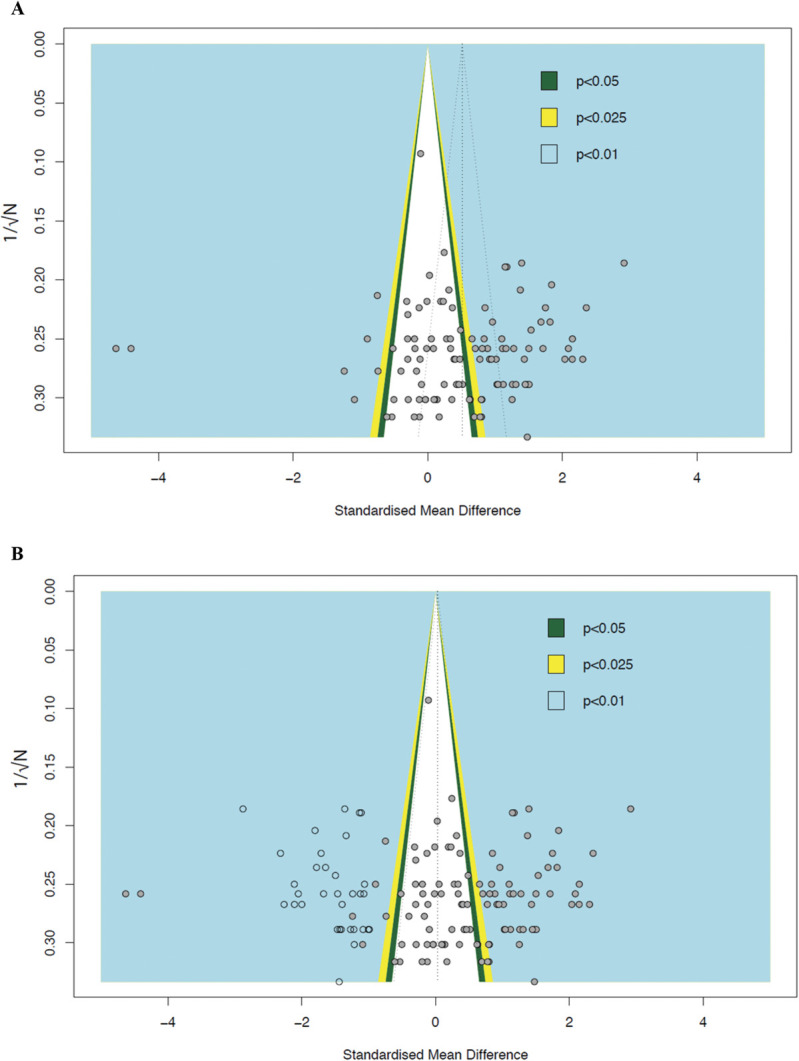
Assessment of publication bias in intervention experiments of rats and mice. (A) Visual inspection of the funnel plot suggests plot asymmetry, hence the presence of publication bias. (B) Trim-and-fill analysis imputed 33 theoretically missing experiments (unfilled circles). The vertical dashed line represents the overall effect size. Filled circles represent experiments from the published studies. The coloured backgrounds indicate the statistical significance of effect sizes of cohort-level comparisons.

The overall effect size of combined rats and mice intervention data (*k* = 107) is 0.51 [95% CI 0.31-0.72]. Egger's regression test was insignificant (*P* = 1.00), suggesting no funnel plot asymmetry (Fig. 14A). Half of the experiments were reported with significant treatment effects (plotted in the coloured backgrounds on the right hand-side). Trim-and-fill analysis, however, imputed 33 theoretically missing experiments, which suggests the presence of publication bias and the adjusted SMD is 0.03 [95% CI −0.19 to 0.25] (Fig. 14B).

### 3.6. Power analysis

The summary modelling effect of CFA on burrowing in rats is −1.62 [95% CI −2.07 to −1.18] (*k* = 19). Using the most conservative estimate of the CI, the total number of animals required to obtain 80% power with a significance level of 0.05 is 26, meaning the number of rats required for each group is 13.

### 3.7. Correlation of burrowing and limb withdrawal behavioural outcomes

**Figure 15.**
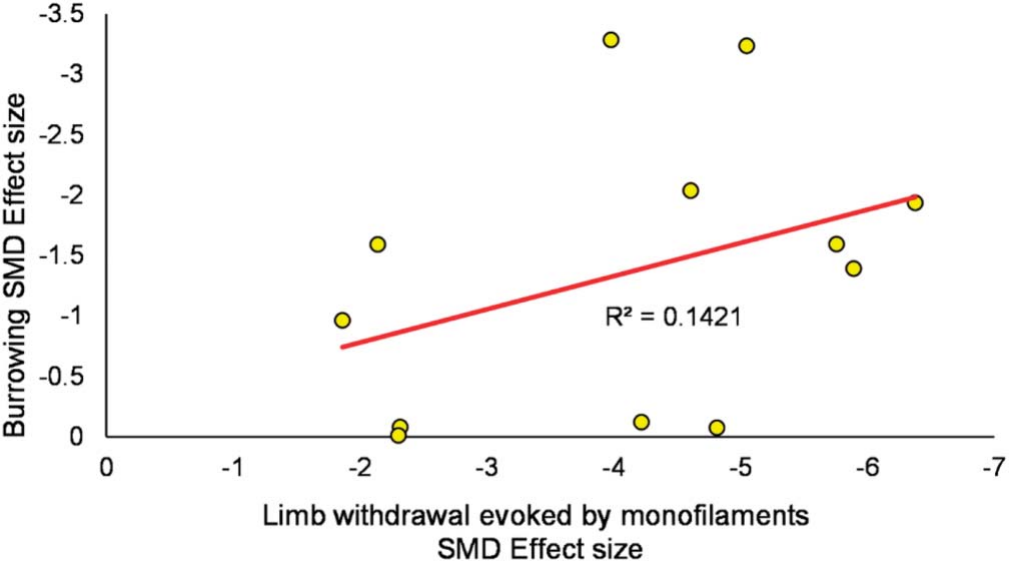
Correlation analysis between burrowing and limb withdrawal behaviours in trauma-induced neuropathy model. Results show that there is a poor correlation for this data set. A line was fitted using the least square method with subsequent *R*^2^ calculation. SMD, standardised mean difference.

There is a poor correlation between burrowing and limb withdrawal outcomes in animals modelled with trauma-induced neuropathy (*k* = 12; *R*^2^ = 0.1421) (Fig. 15).

### 3.8. Dose-response relationships

#### 3.8.1. Dose response in disease modelled animals

**Figure 16.**
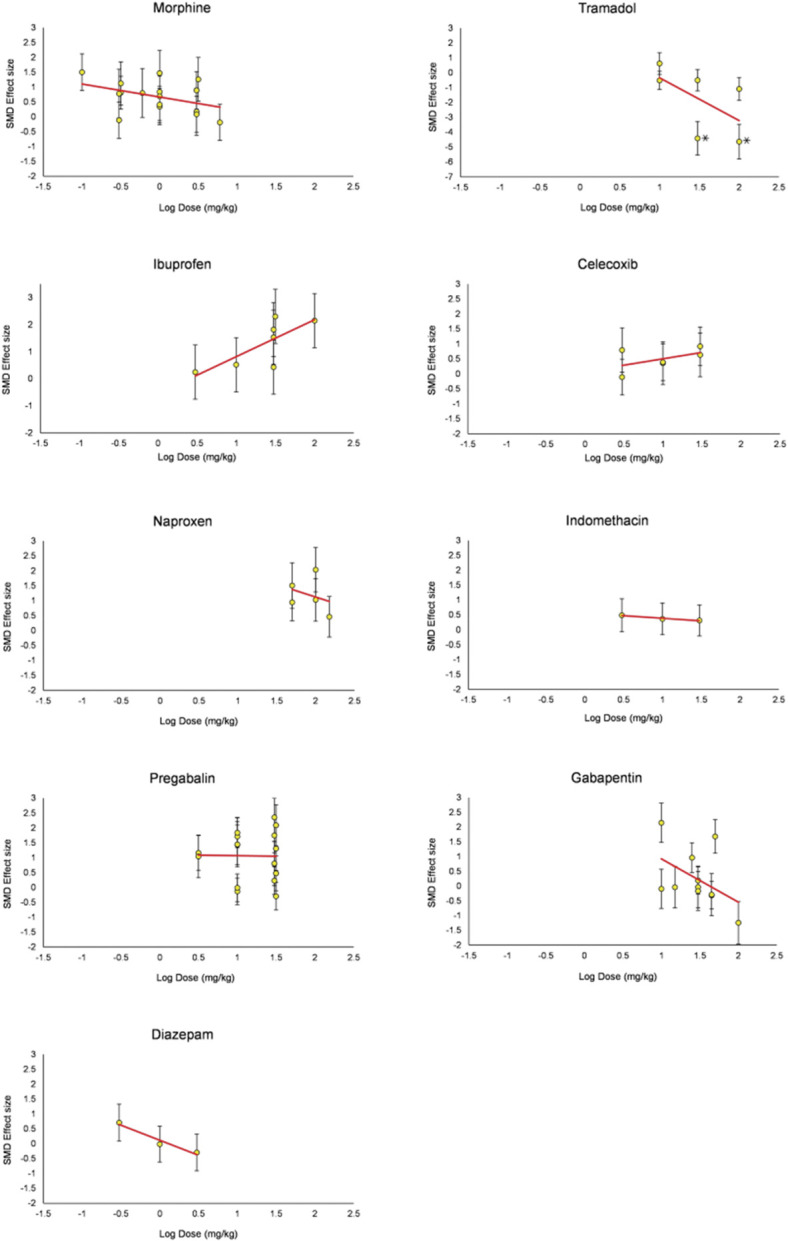
Dose-response curves for analgesic drugs administered in animals modelled with disease models associated with persistent pain. Only experiments that used single administration were used. *P value <0.05 for unpaired *t* test results of cohort-level comparisons. SMD, standardised mean difference.

Increasing doses of morphine, tramadol, gabapentin, and diazepam reduced burrowing. Contrastingly, increasing doses of ibuprofen and celecoxib increased burrowing. No change was observed for naproxen, indomethacin, and pregabalin (Fig. 16).

#### 3.8.2. Dose response in sham or naive animals

**Figure 17.**
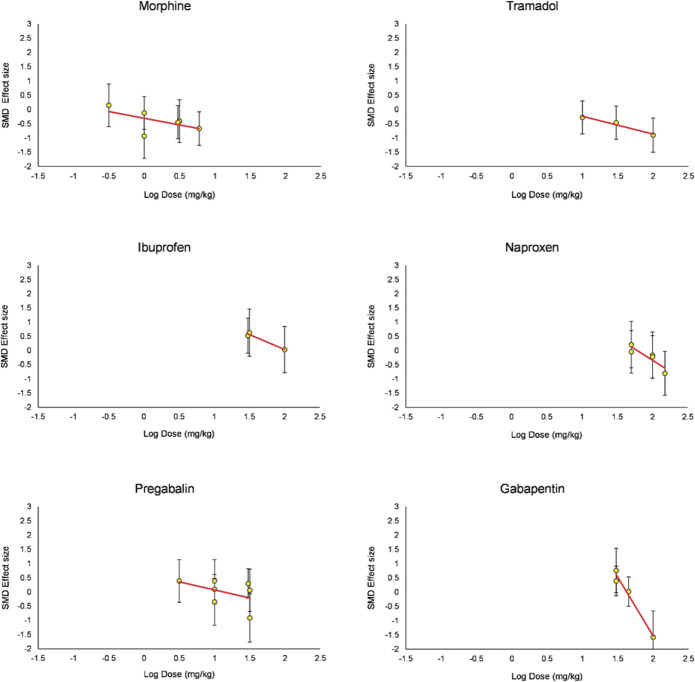
Dose-response curves for analgesic drugs administered in sham or naive animals. Only experiments that used single administration were used. SMD, standardised mean difference.

All pharmacological analgesics reduced burrowing at higher doses (Fig. 17); however, some (morphine, tramadol, naproxen, and gabapentin) reduced below “0” SMD effect size.

### 3.9. Drug effect on naive animals

**Figure 18.**
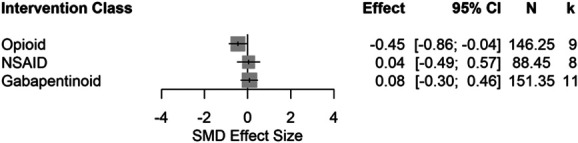
Forest plots of drug treatment effects on burrowing behaviour in naive rats. The size of the square represents the weight. CI, confidence interval; *k*, number of cohort-level comparisons; N, number of animals; NR, not reported; SMD, standardised mean difference.

Opioids significantly reduced burrowing behaviour in naive rats (SMD = −0.45 [95% CI −0.86 to −0.04]) (Fig. 18).

### 3.10. Others

#### 3.10.1. Reporting quality

All studies included in this systematic review were published after 2010; 26% stated reporting in accordance with the ARRIVE guidelines, only 2 provided the checklist, and the remaining 74% did not report in accordance with any reporting guidelines.

#### 3.10.2. Abstract spin

Of the 48 included studies, 1 study was identified to have spin in the abstract conclusion. The authors included interpretation that was not consistent with the study design or the results (Table 5).

**Table 5:** Evidence of spins in the abstract conclusion.

Study	Spin	Reason
Gould et al.^17^	Burrowing […] is suppressed in a model of inflammatory pain and differently reinstated by clinically efficacious analgesics that lack motor impairing side effects, but not an anxiolytic, suggesting that this assay is suitable for the assessment of analgesic efficacy of novel drugs.	Authors did not conduct motor tests to verify that these analgesics do not impair burrowing. Some analgesics in the study did not reinstate burrowing deficits, so burrowing assay may not be suitable for the assessment of novel analgesics.

#### 3.10.3. Reporting of other study characteristics

Animal suppliers for each mouse and rat strains that were used in experiments are listed in the Supplemental Digital Content 9 (available at http://links.lww.com/PAIN/B603). The N range and its median of each disease models used in mouse and rat experiments are listed in the Supplemental Digital Content 10 (available at http://links.lww.com/PAIN/B603). We also collected information regarding acclimatisation, animal husbandry, and experimental conditions for each report, and they are summarised in the Supplemental Digital Content 11 (available at http://links.lww.com/PAIN/B603).

#### 3.10.4. Methodological citing

Of the 45 studies for meta-analysis, 87% (39 studies) cited publications for the burrowing assessment protocol. A total of 18 studies, published between 2001 and 2018, were referenced (Supplemental Digital Content 12, available at http://links.lww.com/PAIN/B603). Most included studies assessed the weight displaced of the burrowing substrate. Of note, 20% (9 included studies) assessed burrowing using other metrics, such as burrowing duration and latency to burrow.

#### 3.10.5. Curated content

Of the 48 included studies, 38% (18 studies) confirmed that changes in burrowing behaviour were not influenced by motor perturbation. Of the 25 studies that assessed drug treatment effects on burrowing behaviour, 28% (7 studies) confirmed that changes in burrowing behaviour were not due to motor perturbations caused by drug treatment and 20% (5 studies) reported conducting pilot experiments to determine the analgesic doses that do not suppress the burrowing behaviour in naive animals.

## 4. Discussion

The purpose of this systematic review was to investigate whether burrowing behaviour represents an appropriate outcome measure to assess rodent disease models of injury or pathological-related persistent pain. This review also aimed to inform the impacts of animal characteristics on rodent burrowing behaviour. We expect that our summary of empirical evidence will assist researchers in making appropriate selection of animal models, outcome measures, and interventions for future experimental designs.

Our systematic review identified 48 studies. Of which, 45 studies were used in the meta-analysis, comprising the effects of 16 disease models associated with persistent pain and 27 classes of drug interventions on burrowing behaviour in 3232 rodents. Our analysis suggests that burrowing is an adequate pain-related ethologically relevant outcome measure in rats as decreased burrowing behaviour was associated with somatic inflammation and trauma-induced neuropathy. Furthermore, this generic behaviour was contextualised to pain as burrowing deficits in rats with experimental persistent pain were attenuated by gabapentinoid and NSAID drug classes. Burrowing is generally considered to be more ethologically relevant to rats than mice,^3^ although previous studies have shown that some laboratory mouse strains have exhibited burrowing behaviour.^10,11^ The magnitude of how mice burrowing was affected by disease models and drug interventions could not be determined because of limited data; hence, it remains unclear whether burrowing is also an appropriate pain-related outcome measure for use in mice. Subgroup analyses were conducted to gain useful insights into how rat model characteristics and drug classes influence the burrowing behaviour; however, some analyses were restricted because of limited data.

### 4.1. Rat burrowing was influenced by animal characteristics and burrowing substrates

Ten types of rat models associated with persistent pain were reported; we were unable to determine whether the magnitude of burrowing deficits was influenced by the type of model used because of limited data.

The largest effect of disease modelling and drug treatments on burrowing outcome was observed in Sprague–Dawley rats, but these effects were not observed in mostly other strains, except for the Wistar Hannover strain in drug intervention experiments. We are unable to ascertain differences in the burrowing outcome between strains because of the predominance of reports of the use of Sprague–Dawley rats. Other systematic reviews of rodent pain research also observed the predominant use of Sprague–Dawley rats.^8,50^ This raises the issue of homogeneity in the rat strain used for preclinical pain research. Hestehave et al.^23^ recently showed that the development of pain-related and anxio-depressive behaviours in response to peripheral nerve injury in rats is strain dependent. In the CFA model, they demonstrated that the efficacy of morphine at the same dosage varied between rat strains.^22^ Hence, researchers need to use animals with diverse genetic profiles to increase the translatability and generalisability of the results to the heterogeneous human patient populations.

Studies were predominantly conducted using male animals, which limits our ability to discern the influence of sex on the burrowing outcome. The lack of research on female animals also raises concerns about the generalisability of findings and their clinical relevance; importantly, women are more likely to be affected by some forms of chronic pain and experience greater pain intensity than men.^33^ It is crucial to use animal models that represent the clinical population so that the translatability of preclinical research can increase. We advocate for a sex balance in preclinical pain research, and several funding bodies such as the National Institutes of Health^7^ and Canadian Institute of Health Research^53^ require this.

The choice of the burrowing substrate in most rat studies was either gravel or sand, which aligns with the finding of Deacon et al.^10^ that rats burrow earth-like substrates well. Previous studies also found that rats do not readily burrow food pellets like mice^6,10,11^; however, we identified 1 study that used food pellets, and burrowing was significantly reduced in trauma-induced neuropathy rats. We could not conclude whether food pellets represent appropriate substrates for rats. We observed greater burrowing attenuation by drug treatments in rats which burrowed gravel as opposed to sand; however, the presence of other confounding factors (eg, model types, drug interventions, strain, and sex) limits our ability to determine the reason behind this observation.

The most frequently used burrowing metric was substrate weight displaced. This was the original outcome metric developed by Deacon et al.^11^ to assess rodent burrowing and was used in the first preclinical pain study assessing rodent burrowing behaviour by Andrews et al.^1^ Interestingly, a small number of studies used alternative metrics, eg, duration of burrowing and latency to burrow. These alternative metrics were first introduced by Jirkof et al.,^29^ which were measured in mice; however, the authors did not provide justification for choosing these measures. Researchers should carefully evaluate the appropriateness of using alternative metrics in drug efficacy studies, for example, half-lives of drugs relating to latency to first burrow. The correlation between types of burrowing metrics and effect sizes remains uncertain. It is early to decide what the most appropriate metrics are, but the most frequently reported is amount displaced, and this has face validity.

### 4.2. Efficacy of gabapentinoids, nonsteroidal anti-inflammatory drugs, and opioids in improving burrowing deficits in rats

Gabapentinoid and NSAID classes attenuated burrowing deficits in rats induced with pain-associated models; however, opioids did not. We could not ascertain how sex and strain influence the treatment effect of the 3 drug classes as experiments were predominantly conducted in male and Sprague–Dawley rats. The burrowing outcome was not influenced by the drug class, but the analysis did not take into account the disease model or other factors. There may not have been enough experimental data from the 3 drug classes to accurately determine the effect.

#### 4.2.1. Gabapentinoids

Pregabalin significantly attenuated burrowing deficits, whereas gabapentin did not. The gabapentin lack of overall efficacy could be due to its sedative effect at higher doses; however, this should be confirmed in a prospective experiment. Pregabalin significantly attenuated rat burrowing deficits caused by arthropathy and trauma-induced neuropathy, and gabapentin significantly attenuated rat burrowing deficits caused by trauma-induced neuropathy. Conversely, effects were not observed in somatic inflammation rats treated with gabapentin, spinal cord injury rats treated with pregabalin, and diabetic-induced neuropathy rats treated with both gabapentinoids. This varied treatment effect in inflammatory and neuropathy conditions may be due to underpowered analysis. This mixed efficacy of gabapentinoids for neuropathic pain has also been clinically observed; a Cochrane Systematic Review found that pregabalin was effective in attenuating shingles or diabetic-induced neuropathic pain but was not effective in attenuating HIV-induced neuropathic pain.^12,15,58^ We were unable to provide a more in-depth analysis to compare the efficacy of pregabalin and gabapentin in attenuating burrowing deficits associated within the same pathological conditions. Overall, our current analysis suggests pregabalin may be used as a positive control when assessing novel drug efficacy in rat models of arthropathy, spinal cord injury, and trauma-induced neuropathy; and gabapentin may be used as a positive control in novel drug efficacy studies conducted in rodent models of trauma-induced neuropathy.

#### 4.2.2. Nonsteroidal anti-inflammatory drugs

All NSAIDs except naproxen significantly attenuated burrowing deficits associated with somatic inflammation and arthropathy. A plausible explanation for the lack of significant effect from naproxen is that the dose range used by studies may not be appropriate. The dose response assessment revealed a reduction of burrowing in naive rats treated with higher doses of naproxen. This is interesting as NSAIDs are not normally associated with motor impairment. In general, the significant treatment efficacy of NSAIDs was expected as they are widely used to treat inflammatory conditions, such as rheumatoid arthritis.^25^ Overall, our analysis indicates indomethacin, celecoxib, and ibuprofen may be used as comparators in studies investigating the efficacy of novel drugs in improving burrowing deficits associated with somatic inflammation and arthropathy.

#### 4.2.3. Opioids

Only morphine significantly attenuated burrowing deficits. The dose range of tramadol was associated with worsening of the burrowing outcome in both naive and disease-modelled rats. Tramadol is pharmacologically less potent and efficacious than morphine,^16^ which may explain the lack of efficacy; however, this must be confirmed in a prospective experiment. Rats with arthropathy and trauma-induced neuropathy were only given morphine, which showed significant efficacy. Due to limited data, we could not separately compare the efficacy of morphine and tramadol within the same pathological conditions. Our analysis suggests that morphine and tramadol were not effective for somatic inflammation and diabetic-induced neuropathy; the efficacy of morphine in these conditions would need to be confirmed when more data become available. Overall, morphine may be used as a comparator for the assessment of novel drugs in rat models of arthropathy and trauma-induced neuropathy.

### 4.3. Dose-response relationships

Morphine, tramadol, gabapentin, and naproxen reduced burrowing at higher doses in naive and disease-modelled animals. However, the extent to which this is related to analgesia as opposed to adverse effects relating (eg, relating to motor impairment) remains unclear. Higher doses of ibuprofen attenuated burrowing deficits without reducing burrowing in naive animals. This suggests that burrowing behaviour may be affected differently according to the analgesic and other pharmacological effects of administered drugs.

The low number of studies investigated the influence of motor perturbation on rodent burrowing. In addition, fewer studies confirmed that changes in rodent burrowing were not caused by drug-associated motor perturbations, and pilot experiments were rarely reported to determine the analgesic doses that do not result in adverse motor effects. It is important to concurrently evaluate motor activities of animals to ascertain that the observed effects were not confounded by treatment-induced motor debilitation.

### 4.4. Correlation of burrowing and monofilament-evoked limb withdrawal outcomes

Several studies have reported good correlations between stimulus-evoked and spontaneous pain tests^9,41^; however, our analysis suggests a poor correlation between burrowing and monofilament-evoked limb withdrawal in animals with trauma injury. Pain is a multidimensional experience, and different behavioural tests capture different aspects of this experience. Stimulus-evoked and ethologically pain-related behavioural paradigms are conceptually and methodologically different from each other, which might explain the poor correlation observed in this data set.

### 4.5. Internal validity

Most studies have an unclear risk of bias. Risk of bias mitigation measures reported within the included studies may differ from what was conducted because of poor methods reporting. Seven reports transparently stated that randomisation was not performed for the purpose of matching basal burrowing activity in control and treatment groups. Although allocating based on the burrowing activity can decrease between-animal variability, the risk of selection bias persists. Two reports transparently stated that a sample size calculation was not performed which raises concerns about the reliability of their results. Nevertheless, the reporting of a sample size calculation is better in the burrowing literature compared with other pain preclinical systematic reviews.^8,14,50^ Animal exclusion should also be reported transparently because inappropriate exclusions could lead to attrition bias and inaccurate effect size estimates.

Larger effect sizes were associated with the reporting of sample size calculations in drug intervention experiments. The influence of allocation concealment on burrowing effect sizes remains unclear. The reporting of blinding was also infrequent which could be caused by the inability to perform blinding because of animals showing prominent symptoms such as oedema induced by inflammatory models. Another possibility is that, unlike the stimulus-evoked behaviours, burrowing can be measured objectively so its association with lower risk of subjective bias could lead researchers to incorrectly posit that blinding is not necessary. Blinding should always be performed to ensure subjective bias is mitigated.

Risk of bias could not be accurately assessed, and the internal validity of the included studies is uncertain because bias mitigation methods were rarely reported. Researchers should ensure experimental conduct is rigorous and reported in sufficient detail in accordance with an established reporting guideline (ie, ARRIVE^37^). Researchers can avoid spin by only reporting findings and interpretations that are supported by the evidence and are consistent with the study design.

### 4.6. Publication bias

Publication bias is the phenomenon by which studies reporting “positive” findings are more likely to be published than studies reporting data where the hypothesis was not proven. Publication bias is abundant in preclinical studies.^31,46,54^ Our analysis suggests that publication bias is only present in studies reporting drug treatment effects on burrowing deficits. Our finding could be limited by the statistical tests used and may be further limited by our data characteristics, small sample sizes, and continuous outcomes.^28^ Trim-and-fill analysis determines publication bias based on plot asymmetry; however, plot asymmetry can also be caused by other factors such as study quality and between-study heterogeneity.^51^ The supposed missing studies are mostly present in areas of significance, which suggests that asymmetry is probably because of factors other than reporting bias according to Peters et al.^39^ Hence, trim-and-fill analysis may incorrectly adjust studies that are not missing which led to the observed overestimation.

### 4.7. Limitations

We could only rely on the information reported in publications. For example, it is possible that methods used to mitigate the risk of bias were implemented but not reported; conversely, studies may have reported conducting these methodological quality measures when they were not. It is possible that the risk of bias assessment lacked power because of the low prevalence of reporting methodological quality measures, resulting in the inconsistent relationship between the reporting of methodological quality measures and effect sizes. There were 3 studies that met the inclusion criteria but could not be included in the meta-analysis because of not reporting variance. Given the small sample size of the studies that were excluded from the meta-analysis, it is unlikely that the overall conclusion would change if that missing information is later provided.

We could not compare different characteristics (ie, strain, sex, substrate, and drug intervention) within the same disease conditions because of limited data.

Information regarding other study characteristics, such as animal husbandry and experimental conditions, was not reported frequently or in sufficient detail to investigate their associations with the burrowing outcome.

We chose to extract behavioural data at the time point at which there was the largest difference between control and treatment animals. This enabled us to calculate treatment effects regardless of their duration, but this limited our ability to investigate different treatment timings. To address this limitation, we extracted the following information: The time between model induction and the first or last behavioural assessment, how long before or after the model was induced was the first dose administered, and how long after the treatment started was the first behavioural assessment. However, because of broad variation and a low number of cohort-level comparisons, we could not investigate this further.

Disease models were grouped according to their broad mechanistic pain classification although the underlying aetiology may vary. Similarly, drug interventions were grouped according to their mechanisms of action regardless of their other properties.

## 5. Conclusion

This systematic review and meta-analysis provides a comprehensive summary of studies that investigated the effect of disease models associated with persistent pain and drug interventions on rodent burrowing behaviour. Burrowing represents an adequate behavioural outcome to assess the impact of persistent pain in rats; its full validity should be confirmed when more data from different persistent pain-related disease model types become available. Based on our analysis, suggestions regarding the drugs which may be deployed as suitable positive controls in certain rat disease models were also made. Consideration should be given to species, strain, and sex when designing experiments. The use of protocols and reporting guidelines will improve the internal validity and assessment of reliability of results. Depending on the declared primary efficacy outcome in a registered protocol, researchers can use our meta-data for power analyses. There was no clear correlation between burrowing and limb withdrawal outcomes. Researchers should measure a portfolio of composite of stimulus-evoked and ethologically relevant behavioural outcomes to improve validity and maximise the information gained from preclinical pain research.

## Conflict of interest statement

J. Vollert received consultancy fees from Vertex Pharmaceuticals and Embody Orthopaedic, outside of the submitted work. A. S.C. Rice conflicts of interest occurring in last 24 months: (1) A. S.C. Rice undertakes consultancy and advisory board work for Imperial College Consultants; in the last 36 months, this has included remunerated work for Abide, Confo, Vertex, Pharmanovo, Lateral, Novartis, Mundipharma, Orion, Shanghai SIMR BiotechAsahi Kasei, and Toray & Theranexis; (2) A. S.C. Rice was the owner of share options in Spinifex Pharmaceuticals from which personal benefit accrued on the acquisition of Spinifex by Novartis in July 2015. The final payment was made in 2019. (3) A. S.C. Rice is named as an inventor on patents: (i) Rice ASC, Vandevoorde S, Lambert DM. Methods using N-(2-propenyl)hexadecanamide and related amides to relieve pain. WO 2005/079771; (ii) Okuse K, et al. Methods of treating pain by inhibition of vgf activity EP13702262.0/WO2013 110945; (4) Councillor International Association Study of Pain; (5) National Institute for Health Research (NIHR)—Chair of the Trial Steering Committee (TSC) for the OPTION-DM trial; (6) Advisor British National Formulary; (7) Member Joint Committee on Vaccine and Immunisation—varicella sub-committee; (8) Analgesic Clinical Trial Translation: Innovations, Opportunities, and Networks (ACTTION) steering committee member; (9) Non Freezing Cold Injury Independent Senior Advisory Committee (NISAC): Member; and (10) Medicines and Healthcare products Regulatory Agency (MHRA), Commission on Human Medicines—Neurology, Pain & Psychiatry Expert Advisory Group. The remaining authors have no conflicts of interest to declare.

## Appendix A. Supplemental digital content

Supplemental digital content associated with this article can be found online at http://links.lww.com/PAIN/B603.

## Supplementary Material

SUPPLEMENTARY MATERIAL
