# Supplementary material for: Systematic review and meta-analysis of studies in which burrowing behaviour was assessed in rodent models of disease-associated persistent pain
Source: Pain. 2022 Mar 29;163(11):2076–102. doi: 10.1097/j.pain.0000000000002632 (PMC9578533; doi:10.1097/j.pain.0000000000002632)
Supplement: SUPPLEMENTARY MATERIAL [file jop-163-02076-s001.pdf]

S1

The followings are separate search strategies for each database:

PubMed/Medline

No.	Component	Terms
1	Burrowing	burrow*.mp. [mp=title, abstract, original title, name of substance word, subject heading word, floating sub-heading word, keyword heading word, organism supplementary concept word, protocol supplementary concept word, rare disease supplementary concept word, unique identifier, synonyms]
AND	AND	AND
2	Pain	“pain”[Mesh] OR "hyperalgesia"[Mesh] OR pain[tiab] OR analgesia[tiab] OR analgesic[tiab] OR analgesics[tiab] OR allodynia[tiab] OR neuralgia[tiab] OR hypersensitivity[tiab] OR hyperalgesia[tiab] OR hyperalgesic[tiab] OR antinociception[tiab] OR anti-nociception[tiab] OR hypoalgesia[tiab] OR hypoalgesic[tiab] OR anti-hyperalgesia[tiab] OR antihyperalgesia[tiab] OR antihyperalgesic[tiab] anti-hyperalgesic[tiab] OR anti-allodynic[tiab] OR antiallodynic[tiab] OR anti-allodynia[tiab] OR antiallodynia[tiab] OR spinal cord injury[tiab] OR nerve injury[tiab] OR nerve injuries[tiab] OR nerve transection[tiab] OR nerve ligation[tiab] OR neuropathy[tiab] OR peripheral neuropathy[tiab] OR polyneuropathy[tiab] OR neuropathic[tiab] OR headache[tiab] OR headache-like[tiab] OR migraine[tiab] OR migraine like[tiab] OR arthritis[tiab] OR osteoarthritis[tiab] OR rheumatoid arthritis[tiab] OR colitis[tiab]
AND	AND	AND
3	Animal filters	(“animal experimentation”[MeSH Terms] OR “models, animal”[MeSH Terms] OR “invertebrates”[MeSH Terms] OR “Animals”[Mesh:noexp] OR “animal population groups”[MeSH Terms] OR “chordata”[MeSH Terms:noexp] OR “chordata, nonvertebrate”[MeSH Terms] OR “vertebrates”[MeSH Terms:noexp] OR “amphibians”[MeSH Terms] OR “birds”[MeSH Terms] OR “fishes”[MeSH Terms] OR “reptiles”[MeSH Terms] OR “mammals”[MeSH Terms:noexp] OR “primates”[MeSH Terms:noexp] OR “artiodactyla”[MeSH Terms] OR “carnivora”[MeSH Terms] OR “cetacea”[MeSH Terms] OR “chiroptera”[MeSH Terms] OR “elephants”[MeSH Terms] OR “hyraxes”[MeSH Terms] OR “insectivora”[MeSH Terms] OR “lagomorpha”[MeSH Terms] OR “marsupialia”[MeSH Terms] OR “monotremata”[MeSH Terms] OR “perissodactyla”[MeSH Terms] OR “rodentia”[MeSH Terms] OR “scandentia”[MeSH Terms] OR “sirenia”[MeSH Terms] OR “xenarthra”[MeSH Terms] OR “haplorhini”[MeSH Terms:noexp] OR “strepsirhini”[MeSH Terms] OR “platyrrhini”[MeSH Terms] OR “tarsii”[MeSH Terms] OR “catarrhini”[MeSH Terms:noexp] OR “cercopithecidae”[MeSH Terms] OR “hylobatidae”[MeSH Terms] OR “hominidae”[MeSH Terms:noexp] OR “gorilla gorilla”[MeSHTerms] OR “pan paniscus”[MeSH Terms] OR “pan troglodytes”[MeSH Terms] OR “pongo pygmaeus”[MeSH Terms]) OR ((animals[tiab] OR animal[tiab] OR mice[Tiab] OR mus[Tiab] OR mouse[Tiab] OR murine[Tiab] OR woodmouse[tiab] OR rats[Tiab] OR rat[Tiab] OR murinae[Tiab] OR muridae[Tiab] OR cottonrat[tiab] OR cottonrats[tiab] OR hamster[tiab] OR hamsters[tiab] OR cricetinae[tiab] OR rodentia[Tiab] OR rodent[Tiab] OR rodents[Tiab] OR pigs[Tiab] OR pig[Tiab] OR swine[tiab] OR swines[tiab] OR piglets[tiab] OR piglet[tiab] OR boar[tiab] OR boars[tiab] OR “sus scrofa”[tiab] OR ferrets[tiab] OR ferret[tiab] OR polecat[tiab] OR polecats[tiab] OR “mustela putorius”[tiab] OR “guinea pigs”[Tiab] OR “guinea pig”[Tiab] OR cavia[Tiab] OR callithrix[Tiab] OR marmoset[Tiab] OR marmosets[Tiab] OR cebuella[Tiab] OR hapale[Tiab] OR octodon[Tiab] OR chinchilla[Tiab] OR chinchillas[Tiab] OR gerbillinae[Tiab] OR gerbil[Tiab] OR gerbils[Tiab] OR jird[Tiab] OR jirds[Tiab] OR merione[Tiab] OR meriones[Tiab] OR rabbits[Tiab] OR rabbit[Tiab] OR hares[Tiab] OR hare[Tiab] OR diptera[Tiab]

		OR flies[Tiab] OR fly[Tiab] OR dipteral[Tiab] OR drosophila[Tiab] OR drosophilidae[Tiab] OR cats[Tiab] OR cat[Tiab] OR carus[Tiab] OR felis[Tiab] OR nematoda[Tiab] OR nematode[Tiab] OR nematodes[Tiab] OR sipunculida[Tiab]OR dogs[Tiab] OR dog[Tiab] OR canine[Tiab] OR canines[Tiab] OR canis[Tiab] OR sheep[Tiab]OR sheeps[Tiab] OR mouflon[Tiab] OR mouflons[Tiab] OR ovis[Tiab] OR goats[Tiab] OR goat[Tiab] OR capra[Tiab] OR capras[Tiab] OR rupicapra[Tiab] OR rupicapras[Tiab] OR chamois[Tiab] OR haplorhini[Tiab] OR monkey[Tiab] OR monkeys[Tiab] OR anthropoidea[Tiab] OR anthropoids[Tiab] OR saguinus[Tiab] OR tamarin[Tiab] OR tamarins[Tiab] OR leontopithecus[Tiab] OR hominidae[Tiab] OR ape[Tiab] OR apes[Tiab] OR "pan paniscus"[Tiab] OR bonobo[Tiab] OR bonobos[Tiab] OR "pan troglodytes"[Tiab] OR gibbon[Tiab] OR gibbons[Tiab]OR siamang[Tiab] OR siamangs[Tiab] OR nomascus[Tiab] OR symphalangus[Tiab] OR chimpanzee[Tiab] OR chimpanzees[Tiab] OR prosimian[Tiab] OR prosimians[Tiab] OR "bush baby"[Tiab] OR bush babies[Tiab] OR galagos[Tiab] OR galago[Tiab] OR pongidae[Tiab] OR gorilla[Tiab] OR gorillas[Tiab] OR "pongo pygmaeus"[Tiab] OR orangutan[Tiab] OR orangutans[Tiab] OR lemur[Tiab] OR lemurs[Tiab] OR lemuridae[Tiab] OR horse[Tiab] OR horses[Tiab] OR equus[Tiab] OR cow[Tiab] OR calf[Tiab] OR bull[Tiab] OR chicken[Tiab] OR chickens[Tiab] OR gallus[Tiab] OR quail[Tiab] OR bird[Tiab] OR birds[Tiab] OR quails[Tiab] OR poultry[Tiab] OR poultries[Tiab] OR fowl[Tiab] OR fowls[Tiab] OR reptile[Tiab] OR reptilia[Tiab] OR reptiles[Tiab] OR snakes[Tiab] OR snake[Tiab] OR lizard[Tiab] OR lizards[Tiab] OR alligator[Tiab] OR alligators[Tiab] OR crocodile[Tiab] OR crocodiles[Tiab] OR turtle[Tiab] OR turtles[Tiab] OR amphibian[Tiab] OR amphibians[Tiab] OR amphibia[Tiab] OR frog[Tiab] OR frogs[Tiab] OR bombina[Tiab] OR salientia[Tiab] OR toad[Tiab] OR toads[Tiab] OR "epidalea calamita"[Tiab] OR salamander[Tiab] OR salamanders[Tiab] OR eel[Tiab] OR eels[Tiab] OR fish[Tiab] OR fishes[Tiab] OR pisces[Tiab] OR catfish[Tiab] OR catfishes[Tiab] OR siluriformes[Tiab] OR arius[Tiab] OR heteropneustes[Tiab] OR sheatfish[Tiab] OR perch[Tiab] OR perches[Tiab] OR percidae[Tiab] OR perca[Tiab] OR trout[Tiab] OR trouts[Tiab] OR char[Tiab] OR chars[Tiab] OR salvelinus[Tiab] OR minnow[Tiab] OR cyprinidae[Tiab] OR carps[Tiab] OR carp[Tiab] OR zebrafish[Tiab] OR zebrafishes[Tiab] OR goldfish[Tiab] OR goldfishes[Tiab] OR guppy[Tiab] OR guppies[Tiab] OR chub[Tiab] OR chubs[Tiab] OR tinca[Tiab] OR barbels[Tiab] OR barbus[Tiab] OR pimephales[Tiab] OR promelas[Tiab] OR "poecilia reticulata"[Tiab] OR mullet[Tiab] OR mullets[Tiab] OR eel[Tiab] OR eels[Tiab] OR seahorse[Tiab] OR seahorses[Tiab] OR mugil curema[Tiab] OR atlantic cod[Tiab] OR shark[Tiab] OR sharks[Tiab]OR catshark[Tiab] OR anguilla[Tiab] OR salmonid[Tiab] OR salmonids[Tiab] OR whitefish[Tiab] OR whitefishes[Tiab] OR salmon[Tiab] OR salmons[Tiab] OR sole[Tiab] OR solea[Tiab] OR lamprey[Tiab] OR lampreys[Tiab] OR pumpkinseed[Tiab] OR sunfish[Tiab] OR sunfishes[Tiab] OR tilapia[Tiab] OR tilapias[Tiab] OR turbot[Tiab] OR turbots[Tiab] OR flatfish[Tiab] OR flatfishes[Tiab] OR sciuridae[Tiab] OR squirrel[Tiab] OR squirrels[Tiab] OR chipmunk[Tiab] OR chipmunks[Tiab] OR suslik[Tiab] OR susliks[Tiab] OR vole[Tiab] OR voles[Tiab] OR lemming[Tiab] OR lemmings[Tiab] OR muskrat[Tiab] OR muskrats[Tiab] OR lemmus[Tiab] OR otter[Tiab] OR otters[Tiab] OR marten[Tiab] OR martens[Tiab] OR martes[Tiab] OR weasel[Tiab] OR badger[Tiab] OR badgers[Tiab] OR ermine[Tiab] OR mink[Tiab] OR minks[Tiab] OR sable[Tiab] OR sables[Tiab] OR gulo[Tiab] OR gulos[Tiab] OR wolverine[Tiab] OR wolverines[Tiab] OR mustela[Tiab] OR llama[Tiab] OR llamas[Tiab] OR alpaca[Tiab] OR alpacas[Tiab] OR camelid[Tiab] OR camelids[Tiab] OR guanaco[Tiab] OR guanacos[Tiab] OR chiroptera[Tiab] OR chiropteras[Tiab] OR bat[Tiab] OR bats[Tiab] OR fox[Tiab] OR foxes[Tiab] OR iguana[Tiab] OR iguanas[Tiab] OR xenopus laevis[Tiab] OR parakeet[Tiab] OR parakeets[Tiab] OR parrot[Tiab] OR parrots[Tiab] OR donkey[Tiab] OR donkeys[Tiab] OR mule[Tiab] OR mules[Tiab] OR zebra[Tiab] OR zebras[Tiab] OR shrew[Tiab]OR shrews[Tiab] OR bison[Tiab] OR bisons[Tiab] OR buffalo[Tiab] OR buffaloes[Tiab] OR deer[Tiab] OR deers[Tiab] OR bear[Tiab] OR bears[Tiab] OR panda[Tiab] OR pandas[Tiab] OR"wild hog"[Tiab] OR "wild boar"[Tiab] OR fitchew[Tiab] OR fitch[Tiab] OR beaver[Tiab] OR beavers[Tiab] OR jerboa[Tiab] OR jerboas[Tiab]

		OR capybara[Tiab] OR capybaras[Tiab] OR canine [tiab] OR bovine [tiab] OR porcine [tiab] OR hog [tiab] OR hogs [tiab]) NOT medline[sb])

Embase (via Ovid)

No.	Component	Terms
1	Burrowing	burrow*.mp. [mp=title, abstract, original title, name of substance word, subject heading word, floating sub-heading word, keyword heading word, organism supplementary concept word, protocol supplementary concept word, rare disease supplementary concept word, unique identifier, synonyms]
AND	AND	AND
2	Pain	pain OR hyperalgesia OR pain OR analgesia OR analgesic OR analgesics OR allodynia OR neuralgia OR hypersensitivity OR hyperalgesia OR hyperalgesicOR antinociception OR anti-nociception OR hypoalgesia OR hypoalgesic OR anti-hyperalgesia OR antihyperalgesia OR antihyperalgesic OR anti-hyperalgesic OR anti-allodynic OR antiallodynic OR anti-allodynia OR antiallodynia OR spinal cord injury OR nerve injury OR nerve injuries OR nerve transection OR nerve ligation OR neuropathy OR peripheral neuropathy OR polyneuropathy OR neuropathic OR headache OR headache-like OR migraine OR migraine like OR arthritis OR osteoarthritis OR rheumatoid arthritis OR colitis
AND	AND	AND
3	Animal filters	exp animal experiment/ or exp animal model/ or exp experimental animal/ or exp transgenic animal/ or exp male animal/ or exp female animal/ or exp juvenile animal/ or animal/ or chordata/ or vertebrate/ or tetrapod/ or exp fish/ or amniote/ or exp amphibia/ or mammal/ or exp reptile/ or exp sauropsid/ or therian/ or exp monotremate/ or placental mammals/ or exp marsupial/ or Euarchontoglires/ or exp Afrotheria/ or exp Boreoeutheria/ or exp Laurasiatheria/ or exp Xenarthra/ or primate/ or exp Dermoptera/ or exp Glires/ or exp Scandentia/ or Haplorhini/ or exp prosimian/ or simian/ or exp tarsiiform/ or Catarrhini/ or exp Platyrrhini/ or ape/ or exp Cercopithecidae/ or hominid/ or exp hylobatidae/ or exp chimpanzee/ or exp gorilla/ or exp orang utan/ or (animal or animals or pisces or fish or fishes or catfish or catfishes or sheatfish or silurus or arius or heteropneustes or clarias or gariepinus or fathead minnow or fathead minnows or pimephales or promelas or cichlidae or trout or trouts or char or chars or salvelinus or salmo or oncorhynchus or guppy or guppies or millionfish or poecilia or goldfish or goldfishes or carassius or auratus or mullet or mullets or mugil or curema or shark or sharks or cod or cods or gadus or morhua or carp or carps or cyprinus or carpio or killifish or eel or eels or anguilla or zander or sander or lucioperca or stizostedion or turbot or turbots or psetta or flatfish or flatfishes or plaice or pleuronectes or platessa or tilapia or tilapias or oreochromis or sarotherodon or common sole or dover sole or solea or zebrafish or zebrafishes or danio or rerio or seabass or dicentrarchus or labrax or morone or lamprey or lampreys or petromyzon or pumpkinseed or pumpkinseeds or lepomis or gibbosus or herring or clupea or harengus or amphibia or amphibian or amphibians or anura or salientia or frog or frogs or rana or toad or toads or bufo or xenopus or laevis or bombina or epidalea or calamita or salamander or salamanders or newt or newts or triturus or reptilia or reptile or reptiles or bearded dragon or pogona or vitticeps or iguana or iguanas or lizard or lizards or anguis fragilis or turtle or turtles or snakes or snake or aves or bird or birds or quail or quails or coturnix or bobwhite or colinus or virginianus or poultry or poultries or fowl or fowls or chicken or chickens or gallus or zebra finch or taeniopygia or guttata or canary or canaries or serinus or canaria or parakeet or parakeets or grasskeet or parrot or parrots or psittacine or psittacines or shelduck or tadorna or goose or geese or branta or leucopsis or woodlark or lullula or flycatcher or ficedula or hypoleuca or dove or doves or geopelia or cuneata or duck or ducks or greylag or graylag or anser or harrier or circus pygargus or red knot or great knot or calidris or canutus or godwit or limosa or lapponica or meleagris or gallopavo or jackdaw or corvus or monedula or ruff or philomachus or pugnax or lapwing or peewit or plover or vanellus or swan or cygnus or columbianus or

		bewickii or gull or chroicocephalus or ridibundus or albifrons or great tit or parus or aythya or fuligula or streptopelia or risoria or spoonbill or platalea or leucorodia or blackbird or turdus or merula or blue tit or cyanistes or pigeon or pigeons or columba or pintail or anas or starling or sturnus or owl or athene noctua or pochard or ferina or cockatiel or nymphicus or hollandicus or skylark or alauda or tern or sterna or teal or crecca or oystercatcher or haematopus or ostralegus or shrew or shrews or sorex or araneus or crocidura or russula or european mole or talpa or chiroptera or bat or bats or eptesicus or serotinus or myotis or dasycneme or daubentonii or pipistrelle or pipistrellus or cat or cats or felis or catus or feline or dog or dogs or canis or canine or canines or otter or otters or lutra or badger or badgers or meles or fitchew or fitch or foumart or foulmart or ferrets or ferret or polecat or polecats or mustela or putorius or weasel or weasels or fox or foxes or vulpes or common seal or phoca or vitulina or grey seal or halichoerus or horse or horses or equus or equine or equidae or donkey or donkeys or mule or mules or pig or pigs or swine or swines or hog or hogs or boar or boars or porcine or piglet or piglets or sus or scrofa or llama or llamas or lama or glama or deer or deers or cervus or elaphus or cow or cows or bos taurus or bos indicus or bovine or bull or bulls or cattle or bison or bisons or sheep or sheeps or ovis aries or ovine or lamb or lambs or mouflon or mouflons or goat or goats or capra or caprine or chamois or rupicapra or leporidae or lagomorpha or lagomorph or rabbit or rabbits or oryctolagus or cuniculus or laprine or hares or lepus or rodentia or rodent or rodents or murinae or mouse or mice or mus or musculus or murine or woodmouse or apodemus or rat or rats or rattus or norvegicus or guinea pig or guinea pigs or cavia or porcellus or hamster or hamsters or mesocricetus or cricetulus or cricetus or gerbil or gerbils or jird or jirds or meriones or unguiculatus or jerboa or jerboas or jaculus or chinchilla or chinchillas or beaver or beavers or castor fiber or castor canadensis or sciuridae or squirrel or squirrels or sciurus or chipmunk or chipmunks or marmot or marmots or marmota or suslik or susliks or spermophilus or cynomys or cottonrat or cottonrats or sigmodon or vole or voles or microtus or myodes or glareolus or primate or primates or prosimian or prosimians or lemur or lemurs or lemuridae or loris or bush baby or bush babies or bushbaby or bushbabies or galago or galagos or anthropoidea or anthropoids or simian or simians or monkey or monkeys or marmoset or marmosets or callithrix or cebuella or tamarin or tamarins or saguinus or leontopithecus or squirrel monkey or squirrel monkeys or saimiri or night monkey or night monkeys or owl monkey or owl monkeys or douroucoulis or aotus or spider monkey or spider monkeys or ateles or baboon or baboons or papio or rhesus monkey or macaque or macaca or mulatta or cynomolgus or fascicularis or green monkey or green monkeys or chlorocebus or vervet or vervets or pygerythrus or hominoidea or ape or apes or hylobatidae or gibbon or gibbons or siamang or siamangs or nomascus or symphalangus or hominidae or orangutan or orangutans or pongo or chimpanzee or chimpanzees or pan troglodytes or bonobo or bonobos or pan paniscus or gorilla or gorillas or troglodytes).ti,ab.

Web of Science

No.	Component	Terms
1	Burrowing	ALL= burrow*
AND	AND	AND
2	Pain	ALL=(pain OR hyperalgesia OR pain OR analgesia OR analgesic OR analgesics OR allodynia OR neuralgia OR hypersensitivity OR hyperalgesia OR hyperalgesicOR antinociception OR anti-nociception OR hypoalgesia OR hypoalgesic OR anti-hyperalgesia OR antihyperalgesia OR antihyperalgesic OR anti-hyperalgesic OR anti-allodynic OR antiallodynic OR anti-allodynia OR antiallodynia OR spinal cord injury OR nerve injury OR nerve injuries OR nerve transection OR nerve ligation OR neuropathy OR peripheral neuropathy OR polyneuropathy OR neuropathic OR headache OR headache-like OR migraine OR migraine like OR arthritis OR osteoarthritis OR rheumatoid arthritis OR colitis)
AND	AND	AND
3	Animal filters	TS=((animal experimentation OR models, animal OR invertebrates OR Animals OR animal population groups OR chordata OR chordata, nonvertebrate ORvertebrates

		OR amphibians OR birds OR fishes OR reptiles OR mammals OR primates OR artiodactyla OR carnivora OR cetacea OR chiroptera OR elephants OR hyraxes OR insectivora OR lagomorpha OR marsupialia OR monotremata OR perissodactyla OR rodentia OR scandentia OR sirenia ORxenarthra OR haplorhini OR strepsirhini OR platyrrhini OR tarsii OR catarrhini OR cercopithecidae OR hylobatidae OR hominidae OR gorilla gorilla OR pan paniscus OR pan troglodytes OR pongo pygmaeus) OR (animals OR animal OR mice OR mus OR mouse OR murine OR woodmouse OR rats OR rat OR murinae OR muridae OR cottonrat OR cottonrats OR hamster OR hamsters OR cricetinae OR rodentia OR rodent OR rodents OR pigs OR pig OR swine OR swines OR piglets OR piglet OR boar OR boars OR sus scrofa OR ferrets OR ferret OR polecat OR polecats OR mustela putorius OR guinea pigs OR guinea pig OR cavia OR callithrix OR marmoset OR marmosets OR cebuella OR hapale OR octodon OR chinchilla OR chinchillas OR gerbillinae OR gerbil OR gerbils OR jird OR jirds OR merione OR meriones OR rabbits OR rabbit OR hares OR hare OR diptera OR flies OR fly OR dipteral OR drosophila OR drosophilidae OR cats OR cat OR carus OR felis OR nematoda OR nematode OR nematodes OR sipunculida OR dogs OR dog OR canine OR canines OR canis OR sheep OR sheeps OR mouflon OR mouflons OR ovis OR goats OR goat OR capra OR capras OR rupicapra OR rupicapras OR chamois OR haplorhini OR monkey OR monkeys OR anthropoidea OR anthropoids OR saguinus OR tamarin OR tamarins OR leontopithecus OR hominidae OR ape OR apes OR pan paniscus OR bonobo OR bonobos OR pan troglodytes OR gibbon OR gibbons OR siamang OR siamangs OR nomascus OR symphalangus OR chimpanzee OR chimpanzees OR prosimian OR prosimians OR bush baby OR bush babies OR galagos OR galago OR pongidae OR gorilla OR gorillas OR pongo pygmaeus OR orangutan OR orangutans OR lemur OR lemurs OR lemuridae OR horse OR horses OR equus OR cow OR calf OR bull OR chicken OR chickens OR gallus OR quail OR bird OR birds OR quails OR poultry OR poultries OR fowl OR fowls OR reptile OR reptilia OR reptiles OR snakes OR snake OR lizard OR lizards OR alligator OR alligators OR crocodile OR crocodiles OR turtle OR turtles OR amphibian OR amphibians OR amphibia OR frog OR frogs OR bombina OR salientia OR toad OR toads OR epidalea calamita OR salamander OR salamanders OR eel OR eels OR fish OR fishes OR pisces OR catfish OR catfishes OR siluriformes OR arius OR heteropneustes OR sheatfish OR perch OR perches OR percidae OR perca OR trout OR trouts OR char OR chars OR salvelinus OR minnow OR cyprinidae OR carps OR carp OR zebrafish OR zebrafishes OR goldfish OR goldfishes OR guppy OR guppies OR chub OR chubs OR tinca OR barbels OR barbus OR pimephales OR promelas OR poecilia reticulata OR mullet OR mullets OR eel OR eels OR seahorse OR seahorses OR mugil curema OR atlantic cod OR shark OR sharks OR catshark OR anguilla OR salmonid OR salmonids OR whitefish OR whitefishes OR salmon OR salmons OR sole OR solea OR lamprey OR lampreys OR pumpkinseed OR sunfish OR sunfishes OR tilapia OR tilapias OR turbot OR turbots OR flatfish OR flatfishes OR sciuridae OR squirrel OR squirrels OR chipmunk OR chipmunks OR suslik OR susliks OR vole OR voles OR lemming OR lemmings OR muskrat OR muskrats OR lemmus OR otter OR otters OR marten OR martens OR martes OR weasel OR badger OR badgers OR ermine OR mink OR minks OR sable OR sables OR gulo OR gulos OR wolverine OR wolverines OR mustela OR llama OR llamas OR alpaca OR alpacas OR camelid OR camelids OR guanaco OR guanacos OR chiroptera OR chiropteras OR bat OR bats OR fox OR foxes OR iguana OR iguanas OR xenopus laevis OR parakeet OR parakeets OR parrot OR parrots OR donkey OR donkeys OR mule OR mules OR zebra OR zebras OR shrew OR shrews OR bison OR bisons OR buffalo OR buffaloes OR deer OR deers OR bear OR bears OR panda OR pandas OR wild hog OR wild boar OR fitchew OR fitch OR beaver OR beavers OR jerboa OR jerboas<br>OR capybara OR capybaras))

Scopus

No.	Component	Terms
1	Burrowing	burrow*
AND	AND	AND
2	Pain	pain OR hyperalgesia OR pain OR analgesia OR analgesic OR analgesics OR allodynia OR neuralgia OR hypersensitivity OR hyperalgesia OR hyperalgesic antinociception OR anti-nociception OR hypoalgesia OR hypoalgesia OR antihyperalgesic OR antihyperalgesic OR antihyperalgesic OR anti-hyperalgesic OR anti allodynia OR antiallodynic OR anti-allodynia OR antiallodynia OR spinal cord injury OR nerve injury OR nerve injuries OR nerve transection OR nerve ligation OR neuropathy OR peripheral neuropathy OR polyneuropathy OR neuropathic OR headache OR headache-like OR migraine OR migraine like OR arthritis OR osteoarthritis OR rheumatoid arthritis OR colitis
AND	AND	AND
3	Animal filters	laboratory OR "Laboratory animal" OR "Laboratory animals" OR "Laboratory mouse" OR "Laboratory mice" OR "Laboratory rat" OR "Laboratory rats" OR murine OR murinae OR muridae OR mice OR mouse OR mus OR woodmouse OR rats OR rat OR cottonrat OR cottonrats OR rodent OR r odents OR rodentia OR model OR models OR "Animal model" OR "Animal models" OR "Rodent model" OR "Rodent models" OR "Murine model" OR "Murine models" OR "Rat model" OR "Rat models" OR "Rats model" OR "Mouse model" OR "Mouse models " OR "Mice model" OR in-vivo OR "In-vivo model"

Animal filter (reference)

1. Hooijmans, C. R., Tillema, A., Leenaars, M. & Ritskes-Hoitinga, M. Enhancing search efficiency by means of a search filter for finding all studies on animal experimentation in PubMed. *Lab. Anim.* **44,** 170–5 (2010).
2. de Vries RBM, H. C. T. A. L. M. R.-H. M. Updated version of Embase search filter for animal studies. (2014). doi:10.1177/0023677213494374

S2

Table. The number of cohort-level comparisons and animals for study design characteristics used in rat modelling experiments.

	No. of studies	No. of reports	No. of cohort-level comparisons	No. of animals
**Disease Model**				
Somatic inflammation	5	12	20	371
Neuropathy: Trauma injury	7	9	13	230
Arthropathy	3	3	9	156
Procedure-associated pain	2	2	3	57
Cancer	1	1	1	50
Mucositis	1	1	1	32
Neuropathy: Diabetic-induced	1	1	1	29
Neuropathy: Antiretroviral-induced	1	1	1	15
Neuropathy: Chemotherapy-induced	1	1	1	12
Migraine	1	1	1	10
**Rat Strain**				
Sprague Dawley	13	16	27	542
Wistar	5	6	11	175
Wistar Hannover	3	4	10	167
LEW/CrlCrlj	1	1	1	30
Zucker diabetic fatty obese	1	1	1	29
Lewis	1	1	1	19
**Sex**				
Male	17	26	48	904
Not reported	1	1	1	29
Female	2	2	2	29
**Substrate Type**				
Gravel	13	19	30	580
Sand	5	6	18	331

Food pellet	2	2	2	32
Not reported	1	1	1	19
**Burrowing Outcome**				
Amount displaced	19	28	51	962

S3

Burrowing Model Sensitivity

```
# meta-analysis
##
SMD
## Andrews et al., 2012; Neuropathy: Trauma Injury; Wistar; NR
-0.1207
## Andrews et al., 2012; Neuropathy: Trauma Injury; Wistar; NR
-0.0806
## Andrews et al., 2012; Somatic inflammation; Sprague Dawley; NR
-2.5770
## Andrews et al., 2012; Neuropathy: Trauma Injury; Sprague Dawley;
NR          -1.9370
## Deseure and Hans, 2018; Neuropathy: Trauma Injury; Sprague
Dawley; Male      -1.5933
## Gould et al., 2016; Somatic inflammation; Sprague Dawley; Male
-1.9651
## Gould et al., 2016; Somatic inflammation; Sprague Dawley; Male
-4.3292
## Gould et al., 2016; Somatic inflammation; Sprague Dawley; Male
-3.5585
## Gould et al., 2016; Somatic inflammation; Sprague Dawley; Male
-1.6867
## Gould et al., 2016; Somatic inflammation; Sprague Dawley; Male
-1.7664
## Gould et al., 2016; Somatic inflammation; Sprague Dawley; Male
-1.1980
## Guimaraes et al., 2019; Neuropathy: Trauma Injury; Sprague
Dawley; Male      -1.5926
## Guimaraes et al., 2019; Neuropathy: Trauma Injury; Sprague
Dawley; Male      -3.2349
## Guimaraes et al., 2019; Neuropathy: Trauma Injury; Sprague
Dawley; Male      -0.0117
## Guimaraes et al., 2019; Neuropathy: Trauma Injury; Sprague
Dawley; Male      -0.0735
## Huang et al., 2013; Neuropathy: Trauma Injury; Wistar; Male
-1.9736
## Lau et al., 2013; Neuropathy: Trauma Injury; Sprague Dawley; Male
-0.9625
## Muralidharan et al., 2016; Neuropathy: Trauma Injury; Sprague
Dawley; Male -1.3928
## Muralidharan et al., 2016; Somatic inflammation; Sprague Dawley;
Male        -1.7072
## Rutten et al., 2018; Neuropathy: Trauma Injury; Sprague Dawley;
Male         -3.4403
```

```
## Rutten et al., 2018; Neuropathy: Trauma Injury; Wistar Hannover;
Male      -1.1409
## Smith et al., 2016; Somatic inflammation; Wistar; Male
0.8574
## Wodarski et al., 2016; Somatic inflammation; Sprague Dawley; Male
-3.2276
## Wodarski et al., 2016; Somatic inflammation; Sprague Dawley; Male
-2.2121
## Wodarski et al., 2016; Somatic inflammation; Sprague Dawley; Male
-0.6797
## Wodarski et al., 2016; Somatic inflammation; Wistar; Male
-0.6756
## Wodarski et al., 2016; Somatic inflammation; Wistar; Male
-1.2798
## Wodarski et al., 2016; Somatic inflammation; Wistar; Male
-0.7437
## Wodarski et al., 2016; Somatic inflammation; Wistar; Male
-1.8361
## Wodarski et al., 2016; Somatic inflammation; Wistar; Male
-1.0730
## Wodarski et al., 2016; Somatic inflammation; Wistar Hannover;
Male         -0.5395
## Wodarski et al., 2016; Somatic inflammation; Sprague Dawley; Male
-1.2205
## Wodarski et al., 2016; Somatic inflammation; Wistar Hannover;
Male         -1.8348
##
95%-CI
## Andrews et al., 2012; Neuropathy: Trauma Injury; Wistar; NR
[-0.9780;  0.7367]
## Andrews et al., 2012; Neuropathy: Trauma Injury; Wistar; NR
[-0.9620;  0.8008]
## Andrews et al., 2012; Somatic inflammation; Sprague Dawley; NR
[-3.5778; -1.5762]
## Andrews et al., 2012; Neuropathy: Trauma Injury; Sprague Dawley;
NR        [-4.3986;  0.5246]
## Deseure and Hans, 2018; Neuropathy: Trauma Injury; Sprague
Dawley; Male    [-2.6286; -0.5580]
## Gould et al., 2016; Somatic inflammation; Sprague Dawley; Male
[-3.3794; -0.5507]
## Gould et al., 2016; Somatic inflammation; Sprague Dawley; Male
[-6.5093; -2.1491]
## Gould et al., 2016; Somatic inflammation; Sprague Dawley; Male
[-4.9886; -2.1284]
## Gould et al., 2016; Somatic inflammation; Sprague Dawley; Male
[-2.6872; -0.6862]
```

```
## Gould et al., 2016; Somatic inflammation; Sprague Dawley; Male
[-2.8348; -0.6980]
## Gould et al., 2016; Somatic inflammation; Sprague Dawley; Male
[-2.4641;  0.0680]
## Guimaraes et al., 2019; Neuropathy: Trauma Injury; Sprague
Dawley; Male    [-3.5794;  0.3942]
## Guimaraes et al., 2019; Neuropathy: Trauma Injury; Sprague
Dawley; Male    [-5.0366; -1.4332]
## Guimaraes et al., 2019; Neuropathy: Trauma Injury; Sprague
Dawley; Male    [-1.5439;  1.5205]
## Guimaraes et al., 2019; Neuropathy: Trauma Injury; Sprague
Dawley; Male    [-1.4519;  1.3049]
## Huang et al., 2013; Neuropathy: Trauma Injury; Wistar; Male
[-4.6798;  0.7327]
## Lau et al., 2013; Neuropathy: Trauma Injury; Sprague Dawley; Male
[-1.8163; -0.1087]
## Muralidharan et al., 2016; Neuropathy: Trauma Injury; Sprague
Dawley; Male [-2.1247; -0.6609]
## Muralidharan et al., 2016; Somatic inflammation; Sprague Dawley;
Male      [-2.8031; -0.6112]
## Rutten et al., 2018; Neuropathy: Trauma Injury; Sprague Dawley;
Male       [-5.1316; -1.7490]
## Rutten et al., 2018; Neuropathy: Trauma Injury; Wistar Hannover;
Male      [-2.0259; -0.2559]
## Smith et al., 2016; Somatic inflammation; Wistar; Male
[-0.1806;  1.8954]
## Wodarski et al., 2016; Somatic inflammation; Sprague Dawley; Male
[-4.9845; -1.4708]
## Wodarski et al., 2016; Somatic inflammation; Sprague Dawley; Male
[-3.3755; -1.0487]
## Wodarski et al., 2016; Somatic inflammation; Sprague Dawley; Male
[-1.6965;  0.3371]
## Wodarski et al., 2016; Somatic inflammation; Wistar; Male
[-1.3908;  0.0395]
## Wodarski et al., 2016; Somatic inflammation; Wistar; Male
[-2.3178; -0.2418]
## Wodarski et al., 2016; Somatic inflammation; Wistar; Male
[-1.6826;  0.1952]
## Wodarski et al., 2016; Somatic inflammation; Wistar; Male
[-3.7111;  0.0389]
## Wodarski et al., 2016; Somatic inflammation; Wistar; Male
[-2.6432;  0.4972]
## Wodarski et al., 2016; Somatic inflammation; Wistar Hannover;
Male          [-1.5429;  0.4638]
## Wodarski et al., 2016; Somatic inflammation; Sprague Dawley; Male
[-2.1054; -0.3357]
```

```
## Wodarski et al., 2016; Somatic inflammation; Wistar Hannover; 
Male          [-2.9169; -0.7527]
## 
%W(random)
## Andrews et al., 2012; Neuropathy: Trauma Injury; Wistar; NR 
3.8
## Andrews et al., 2012; Neuropathy: Trauma Injury; Wistar; NR 
3.8
## Andrews et al., 2012; Somatic inflammation; Sprague Dawley; NR 
3.5
## Andrews et al., 2012; Neuropathy: Trauma Injury; Sprague Dawley; 
NR                 1.5
## Deseure and Hans, 2018; Neuropathy: Trauma Injury; Sprague 
Dawley; Male           3.5
## Gould et al., 2016; Somatic inflammation; Sprague Dawley; Male 
2.8
## Gould et al., 2016; Somatic inflammation; Sprague Dawley; Male 
1.7
## Gould et al., 2016; Somatic inflammation; Sprague Dawley; Male 
2.7
## Gould et al., 2016; Somatic inflammation; Sprague Dawley; Male 
3.5
## Gould et al., 2016; Somatic inflammation; Sprague Dawley; Male 
3.4
## Gould et al., 2016; Somatic inflammation; Sprague Dawley; Male 
3.0
## Guimaraes et al., 2019; Neuropathy: Trauma Injury; Sprague 
Dawley; Male           1.9
## Guimaraes et al., 2019; Neuropathy: Trauma Injury; Sprague 
Dawley; Male           2.2
## Guimaraes et al., 2019; Neuropathy: Trauma Injury; Sprague 
Dawley; Male           2.6
## Guimaraes et al., 2019; Neuropathy: Trauma Injury; Sprague 
Dawley; Male           2.8
## Huang et al., 2013; Neuropathy: Trauma Injury; Wistar; Male 
1.3
## Lau et al., 2013; Neuropathy: Trauma Injury; Sprague Dawley; Male 
3.8
## Muralidharan et al., 2016; Neuropathy: Trauma Injury; Sprague 
Dawley; Male        4.0
## Muralidharan et al., 2016; Somatic inflammation; Sprague Dawley; 
Male             3.3
## Rutten et al., 2018; Neuropathy: Trauma Injury; Sprague Dawley; 
Male              2.3
## Rutten et al., 2018; Neuropathy: Trauma Injury; Wistar Hannover; 
Male             3.7
```

```
## Smith et al., 2016; Somatic inflammation; Wistar; Male
3.5
## Wodarski et al., 2016; Somatic inflammation; Sprague Dawley; Male
2.2
## Wodarski et al., 2016; Somatic inflammation; Sprague Dawley; Male
3.2
## Wodarski et al., 2016; Somatic inflammation; Sprague Dawley; Male
3.5
## Wodarski et al., 2016; Somatic inflammation; Wistar; Male
4.1
## Wodarski et al., 2016; Somatic inflammation; Wistar; Male
3.5
## Wodarski et al., 2016; Somatic inflammation; Wistar; Male
3.6
## Wodarski et al., 2016; Somatic inflammation; Wistar; Male
2.1
## Wodarski et al., 2016; Somatic inflammation; Wistar; Male
2.5
## Wodarski et al., 2016; Somatic inflammation; Wistar Hannover;
Male                3.5
## Wodarski et al., 2016; Somatic inflammation; Sprague Dawley; Male
3.8
## Wodarski et al., 2016; Somatic inflammation; Wistar Hannover;
Male                3.4
##
## Number of studies combined: k = 33
##
##                           SMD             95%-CI     t  p-value
## Random effects model -1.3932 [-1.7759; -1.0104] -7.41 < 0.0001
## Prediction interval          [-3.1182;  0.3318]
##
## Quantifying heterogeneity:
##  tau^2 = 0.6801 [0.3245; 1.7126]; tau = 0.8247 [0.5697; 1.3087]
##  I^2 = 64.8% [49.0%; 75.6%]; H = 1.68 [1.40; 2.03]
##
## Test of heterogeneity:
##      Q d.f.  p-value
##  90.84   32 < 0.0001
##
## Details on meta-analytical method:
## - Inverse variance method
## - Restricted maximum-likelihood estimator for tau^2
## - Q-profile method for confidence interval of tau^2 and tau
## - Hartung-Knapp adjustment for random effects model
```

```
# detecting outliers
##                                                         Author
lowerci
## 1 Andrews et al., 2012; Neuropathy: Trauma Injury; Wistar; NR -
0.9780000
## 2 Andrews et al., 2012; Neuropathy: Trauma Injury; Wistar; NR -
0.9619781
## 3      Smith et al., 2016; Somatic inflammation; Wistar; Male -
0.1806308
##     upperci
## 1 0.7366532
## 2 0.8007802
## 3 1.8953616
```

```
# meta-analysis with outliers removal
```

```
##
SMD
## Andrews et al., 2012; Neuropathy: Trauma Injury; Wistar; NR
-0.0806
## Andrews et al., 2012; Somatic inflammation; Sprague Dawley; NR
-2.5770
## Andrews et al., 2012; Neuropathy: Trauma Injury; Sprague Dawley;
NR        -1.9370
## Deseure and Hans, 2018; Neuropathy: Trauma Injury; Sprague
Dawley; Male    -1.5933
## Gould et al., 2016; Somatic inflammation; Sprague Dawley; Male
-1.9651
## Gould et al., 2016; Somatic inflammation; Sprague Dawley; Male
-4.3292
## Gould et al., 2016; Somatic inflammation; Sprague Dawley; Male
-3.5585
## Gould et al., 2016; Somatic inflammation; Sprague Dawley; Male
-1.6867
## Gould et al., 2016; Somatic inflammation; Sprague Dawley; Male
-1.7664
## Gould et al., 2016; Somatic inflammation; Sprague Dawley; Male
-1.1980
## Guimaraes et al., 2019; Neuropathy: Trauma Injury; Sprague
Dawley; Male    -1.5926
## Guimaraes et al., 2019; Neuropathy: Trauma Injury; Sprague
Dawley; Male    -3.2349
## Guimaraes et al., 2019; Neuropathy: Trauma Injury; Sprague
Dawley; Male    -0.0117
## Guimaraes et al., 2019; Neuropathy: Trauma Injury; Sprague
Dawley; Male    -0.0735
## Huang et al., 2013; Neuropathy: Trauma Injury; Wistar; Male
-1.9736
## Lau et al., 2013; Neuropathy: Trauma Injury; Sprague Dawley; Male
-0.9625
## Muralidharan et al., 2016; Neuropathy: Trauma Injury; Sprague
Dawley; Male -1.3928
## Muralidharan et al., 2016; Somatic inflammation; Sprague Dawley;
Male      -1.7072
## Rutten et al., 2018; Neuropathy: Trauma Injury; Sprague Dawley;
Male       -3.4403
## Rutten et al., 2018; Neuropathy: Trauma Injury; Wistar Hannover;
Male      -1.1409
## Smith et al., 2016; Somatic inflammation; Wistar; Male
0.8574
## Wodarski et al., 2016; Somatic inflammation; Sprague Dawley; Male
-3.2276
```

```
## Wodarski et al., 2016; Somatic inflammation; Sprague Dawley; Male
-2.2121
## Wodarski et al., 2016; Somatic inflammation; Sprague Dawley; Male
-0.6797
## Wodarski et al., 2016; Somatic inflammation; Wistar; Male
-0.6756
## Wodarski et al., 2016; Somatic inflammation; Wistar; Male
-1.2798
## Wodarski et al., 2016; Somatic inflammation; Wistar; Male
-0.7437
## Wodarski et al., 2016; Somatic inflammation; Wistar; Male
-1.8361
## Wodarski et al., 2016; Somatic inflammation; Wistar; Male
-1.0730
## Wodarski et al., 2016; Somatic inflammation; Wistar Hannover;
Male         -0.5395
## Wodarski et al., 2016; Somatic inflammation; Sprague Dawley; Male
-1.2205
## Wodarski et al., 2016; Somatic inflammation; Wistar Hannover;
Male         -1.8348
##
95%-CI
## Andrews et al., 2012; Neuropathy: Trauma Injury; Wistar; NR
[-0.9620;  0.8008]
## Andrews et al., 2012; Somatic inflammation; Sprague Dawley; NR
[-3.5778; -1.5762]
## Andrews et al., 2012; Neuropathy: Trauma Injury; Sprague Dawley;
NR        [-4.3986;  0.5246]
## Deseure and Hans, 2018; Neuropathy: Trauma Injury; Sprague
Dawley; Male    [-2.6286; -0.5580]
## Gould et al., 2016; Somatic inflammation; Sprague Dawley; Male
[-3.3794; -0.5507]
## Gould et al., 2016; Somatic inflammation; Sprague Dawley; Male
[-6.5093; -2.1491]
## Gould et al., 2016; Somatic inflammation; Sprague Dawley; Male
[-4.9886; -2.1284]
## Gould et al., 2016; Somatic inflammation; Sprague Dawley; Male
[-2.6872; -0.6862]
## Gould et al., 2016; Somatic inflammation; Sprague Dawley; Male
[-2.8348; -0.6980]
## Gould et al., 2016; Somatic inflammation; Sprague Dawley; Male
[-2.4641;  0.0680]
## Guimaraes et al., 2019; Neuropathy: Trauma Injury; Sprague
Dawley; Male    [-3.5794;  0.3942]
## Guimaraes et al., 2019; Neuropathy: Trauma Injury; Sprague
Dawley; Male    [-5.0366; -1.4332]
```

```
## Guimaraes et al., 2019; Neuropathy: Trauma Injury; Sprague
Dawley; Male    [-1.5439;  1.5205]
## Guimaraes et al., 2019; Neuropathy: Trauma Injury; Sprague
Dawley; Male    [-1.4519;  1.3049]
## Huang et al., 2013; Neuropathy: Trauma Injury; Wistar; Male
[-4.6798;  0.7327]
## Lau et al., 2013; Neuropathy: Trauma Injury; Sprague Dawley; Male
[-1.8163; -0.1087]
## Muralidharan et al., 2016; Neuropathy: Trauma Injury; Sprague
Dawley; Male [-2.1247; -0.6609]
## Muralidharan et al., 2016; Somatic inflammation; Sprague Dawley;
Male      [-2.8031; -0.6112]
## Rutten et al., 2018; Neuropathy: Trauma Injury; Sprague Dawley;
Male       [-5.1316; -1.7490]
## Rutten et al., 2018; Neuropathy: Trauma Injury; Wistar Hannover;
Male      [-2.0259; -0.2559]
## Smith et al., 2016; Somatic inflammation; Wistar; Male
[-0.1806;  1.8954]
## Wodarski et al., 2016; Somatic inflammation; Sprague Dawley; Male
[-4.9845; -1.4708]
## Wodarski et al., 2016; Somatic inflammation; Sprague Dawley; Male
[-3.3755; -1.0487]
## Wodarski et al., 2016; Somatic inflammation; Sprague Dawley; Male
[-1.6965;  0.3371]
## Wodarski et al., 2016; Somatic inflammation; Wistar; Male
[-1.3908;  0.0395]
## Wodarski et al., 2016; Somatic inflammation; Wistar; Male
[-2.3178; -0.2418]
## Wodarski et al., 2016; Somatic inflammation; Wistar; Male
[-1.6826;  0.1952]
## Wodarski et al., 2016; Somatic inflammation; Wistar; Male
[-3.7111;  0.0389]
## Wodarski et al., 2016; Somatic inflammation; Wistar; Male
[-2.6432;  0.4972]
## Wodarski et al., 2016; Somatic inflammation; Wistar Hannover;
Male         [-1.5429;  0.4638]
## Wodarski et al., 2016; Somatic inflammation; Sprague Dawley; Male
[-2.1054; -0.3357]
## Wodarski et al., 2016; Somatic inflammation; Wistar Hannover;
Male         [-2.9169; -0.7527]
##
%W(random)
## Andrews et al., 2012; Neuropathy: Trauma Injury; Wistar; NR
3.9
## Andrews et al., 2012; Somatic inflammation; Sprague Dawley; NR
3.7
```

```
## Andrews et al., 2012; Neuropathy: Trauma Injury; Sprague Dawley;
NR                  1.5
## Deseure and Hans, 2018; Neuropathy: Trauma Injury; Sprague
Dawley; Male              3.6
## Gould et al., 2016; Somatic inflammation; Sprague Dawley; Male
2.9
## Gould et al., 2016; Somatic inflammation; Sprague Dawley; Male
1.8
## Gould et al., 2016; Somatic inflammation; Sprague Dawley; Male
2.8
## Gould et al., 2016; Somatic inflammation; Sprague Dawley; Male
3.7
## Gould et al., 2016; Somatic inflammation; Sprague Dawley; Male
3.5
## Gould et al., 2016; Somatic inflammation; Sprague Dawley; Male
3.1
## Guimaraes et al., 2019; Neuropathy: Trauma Injury; Sprague
Dawley; Male              2.0
## Guimaraes et al., 2019; Neuropathy: Trauma Injury; Sprague
Dawley; Male              2.2
## Guimaraes et al., 2019; Neuropathy: Trauma Injury; Sprague
Dawley; Male              2.7
## Guimaraes et al., 2019; Neuropathy: Trauma Injury; Sprague
Dawley; Male              2.9
## Huang et al., 2013; Neuropathy: Trauma Injury; Wistar; Male
1.3
## Lau et al., 2013; Neuropathy: Trauma Injury; Sprague Dawley; Male
4.0
## Muralidharan et al., 2016; Neuropathy: Trauma Injury; Sprague
Dawley; Male           4.2
## Muralidharan et al., 2016; Somatic inflammation; Sprague Dawley;
Male                3.5
## Rutten et al., 2018; Neuropathy: Trauma Injury; Sprague Dawley;
Male                 2.4
## Rutten et al., 2018; Neuropathy: Trauma Injury; Wistar Hannover;
Male                3.9
## Smith et al., 2016; Somatic inflammation; Wistar; Male
3.6
## Wodarski et al., 2016; Somatic inflammation; Sprague Dawley; Male
2.3
## Wodarski et al., 2016; Somatic inflammation; Sprague Dawley; Male
3.3
## Wodarski et al., 2016; Somatic inflammation; Sprague Dawley; Male
3.6
## Wodarski et al., 2016; Somatic inflammation; Wistar; Male
4.3
```

```
## Wodarski et al., 2016; Somatic inflammation; Wistar; Male
3.6
## Wodarski et al., 2016; Somatic inflammation; Wistar; Male
3.8
## Wodarski et al., 2016; Somatic inflammation; Wistar; Male
2.1
## Wodarski et al., 2016; Somatic inflammation; Wistar; Male
2.6
## Wodarski et al., 2016; Somatic inflammation; Wistar Hannover;
Male                    3.7
## Wodarski et al., 2016; Somatic inflammation; Sprague Dawley; Male
3.9
## Wodarski et al., 2016; Somatic inflammation; Wistar Hannover;
Male                    3.5
##
## Number of studies combined: k = 32
##
##                           SMD            95%-CI     t  p-value
## Random effects model -1.4400 [-1.8248; -1.0552] -7.63 < 0.0001
## Prediction interval          [-3.1272;  0.2471]
##
## Quantifying heterogeneity:
##  tau^2 = 0.6469 [0.2964; 1.6846]; tau = 0.8043 [0.5444; 1.2979]
##  I^2 = 63.2% [46.2%; 74.8%]; H = 1.65 [1.36; 1.99]
##
## Test of heterogeneity:
##      Q d.f.  p-value
##  84.15   31 < 0.0001
##
## Details on meta-analytical method:
## - Inverse variance method
## - Restricted maximum-likelihood estimator for tau^2
## - Q-profile method for confidence interval of tau^2 and tau
## - Hartung-Knapp adjustment for random effects model
```

```
# meta-analysis with removal of studies with high risk of bias
```

```
##
SMD
## Andrews et al., 2012; Neuropathy: Trauma Injury; Wistar; NR
-0.1207
## Andrews et al., 2012; Neuropathy: Trauma Injury; Wistar; NR
-0.0806
## Andrews et al., 2012; Somatic inflammation; Sprague Dawley; NR
-2.5770
## Andrews et al., 2012; Neuropathy: Trauma Injury; Sprague Dawley;
NR         -1.9370
## Deseure and Hans, 2018; Neuropathy: Trauma Injury; Sprague
Dawley; Male    -1.5933
## Gould et al., 2016; Somatic inflammation; Sprague Dawley; Male
-1.9651
## Gould et al., 2016; Somatic inflammation; Sprague Dawley; Male
-4.3292
## Gould et al., 2016; Somatic inflammation; Sprague Dawley; Male
-3.5585
## Gould et al., 2016; Somatic inflammation; Sprague Dawley; Male
-1.6867
## Gould et al., 2016; Somatic inflammation; Sprague Dawley; Male
-1.7664
## Gould et al., 2016; Somatic inflammation; Sprague Dawley; Male
-1.1980
## Guimaraes et al., 2019; Neuropathy: Trauma Injury; Sprague
Dawley; Male    -1.5926
## Guimaraes et al., 2019; Neuropathy: Trauma Injury; Sprague
Dawley; Male    -3.2349
## Guimaraes et al., 2019; Neuropathy: Trauma Injury; Sprague
Dawley; Male    -0.0117
## Guimaraes et al., 2019; Neuropathy: Trauma Injury; Sprague
Dawley; Male    -0.0735
## Huang et al., 2013; Neuropathy: Trauma Injury; Wistar; Male
-1.9736
## Lau et al., 2013; Neuropathy: Trauma Injury; Sprague Dawley; Male
-0.9625
## Muralidharan et al., 2016; Neuropathy: Trauma Injury; Sprague
Dawley; Male -1.3928
## Muralidharan et al., 2016; Somatic inflammation; Sprague Dawley;
Male       -1.7072
## Rutten et al., 2018; Neuropathy: Trauma Injury; Wistar Hannover;
Male       -1.1409
## Smith et al., 2016; Somatic inflammation; Wistar; Male
0.8574
## Wodarski et al., 2016; Somatic inflammation; Sprague Dawley; Male
-3.2276
```

```
## Wodarski et al., 2016; Somatic inflammation; Sprague Dawley; Male
-2.2121
## Wodarski et al., 2016; Somatic inflammation; Sprague Dawley; Male
-0.6797
## Wodarski et al., 2016; Somatic inflammation; Wistar; Male
-0.6756
## Wodarski et al., 2016; Somatic inflammation; Wistar; Male
-1.2798
## Wodarski et al., 2016; Somatic inflammation; Wistar; Male
-0.7437
## Wodarski et al., 2016; Somatic inflammation; Wistar; Male
-1.8361
## Wodarski et al., 2016; Somatic inflammation; Wistar; Male
-1.0730
## Wodarski et al., 2016; Somatic inflammation; Wistar Hannover;
Male         -0.5395
## Wodarski et al., 2016; Somatic inflammation; Sprague Dawley; Male
-1.2205
## Wodarski et al., 2016; Somatic inflammation; Wistar Hannover;
Male         -1.8348
##
95%-CI
## Andrews et al., 2012; Neuropathy: Trauma Injury; Wistar; NR
[-0.9780;  0.7367]
## Andrews et al., 2012; Neuropathy: Trauma Injury; Wistar; NR
[-0.9620;  0.8008]
## Andrews et al., 2012; Somatic inflammation; Sprague Dawley; NR
[-3.5778; -1.5762]
## Andrews et al., 2012; Neuropathy: Trauma Injury; Sprague Dawley;
NR        [-4.3986;  0.5246]
## Deseure and Hans, 2018; Neuropathy: Trauma Injury; Sprague
Dawley; Male    [-2.6286; -0.5580]
## Gould et al., 2016; Somatic inflammation; Sprague Dawley; Male
[-3.3794; -0.5507]
## Gould et al., 2016; Somatic inflammation; Sprague Dawley; Male
[-6.5093; -2.1491]
## Gould et al., 2016; Somatic inflammation; Sprague Dawley; Male
[-4.9886; -2.1284]
## Gould et al., 2016; Somatic inflammation; Sprague Dawley; Male
[-2.6872; -0.6862]
## Gould et al., 2016; Somatic inflammation; Sprague Dawley; Male
[-2.8348; -0.6980]
## Gould et al., 2016; Somatic inflammation; Sprague Dawley; Male
[-2.4641;  0.0680]
## Guimaraes et al., 2019; Neuropathy: Trauma Injury; Sprague
Dawley; Male    [-3.5794;  0.3942]
```

```
## Guimaraes et al., 2019; Neuropathy: Trauma Injury; Sprague
Dawley; Male    [-5.0366; -1.4332]
## Guimaraes et al., 2019; Neuropathy: Trauma Injury; Sprague
Dawley; Male    [-1.5439;  1.5205]
## Guimaraes et al., 2019; Neuropathy: Trauma Injury; Sprague
Dawley; Male    [-1.4519;  1.3049]
## Huang et al., 2013; Neuropathy: Trauma Injury; Wistar; Male
[-4.6798;  0.7327]
## Lau et al., 2013; Neuropathy: Trauma Injury; Sprague Dawley; Male
[-1.8163; -0.1087]
## Muralidharan et al., 2016; Neuropathy: Trauma Injury; Sprague
Dawley; Male [-2.1247; -0.6609]
## Muralidharan et al., 2016; Somatic inflammation; Sprague Dawley;
Male      [-2.8031; -0.6112]
## Rutten et al., 2018; Neuropathy: Trauma Injury; Wistar Hannover;
Male      [-2.0259; -0.2559]
## Smith et al., 2016; Somatic inflammation; Wistar; Male
[-0.1806;  1.8954]
## Wodarski et al., 2016; Somatic inflammation; Sprague Dawley; Male
[-4.9845; -1.4708]
## Wodarski et al., 2016; Somatic inflammation; Sprague Dawley; Male
[-3.3755; -1.0487]
## Wodarski et al., 2016; Somatic inflammation; Sprague Dawley; Male
[-1.6965;  0.3371]
## Wodarski et al., 2016; Somatic inflammation; Wistar; Male
[-1.3908;  0.0395]
## Wodarski et al., 2016; Somatic inflammation; Wistar; Male
[-2.3178; -0.2418]
## Wodarski et al., 2016; Somatic inflammation; Wistar; Male
[-1.6826;  0.1952]
## Wodarski et al., 2016; Somatic inflammation; Wistar; Male
[-3.7111;  0.0389]
## Wodarski et al., 2016; Somatic inflammation; Wistar; Male
[-2.6432;  0.4972]
## Wodarski et al., 2016; Somatic inflammation; Wistar Hannover;
Male         [-1.5429;  0.4638]
## Wodarski et al., 2016; Somatic inflammation; Sprague Dawley; Male
[-2.1054; -0.3357]
## Wodarski et al., 2016; Somatic inflammation; Wistar Hannover;
Male         [-2.9169; -0.7527]
##
%W(random)
## Andrews et al., 2012; Neuropathy: Trauma Injury; Wistar; NR
3.9
## Andrews et al., 2012; Neuropathy: Trauma Injury; Wistar; NR
3.9
```

```
## Andrews et al., 2012; Somatic inflammation; Sprague Dawley; NR
3.6
## Andrews et al., 2012; Neuropathy: Trauma Injury; Sprague Dawley;
NR                 1.4
## Deseure and Hans, 2018; Neuropathy: Trauma Injury; Sprague
Dawley; Male              3.6
## Gould et al., 2016; Somatic inflammation; Sprague Dawley; Male
2.8
## Gould et al., 2016; Somatic inflammation; Sprague Dawley; Male
1.7
## Gould et al., 2016; Somatic inflammation; Sprague Dawley; Male
2.8
## Gould et al., 2016; Somatic inflammation; Sprague Dawley; Male
3.6
## Gould et al., 2016; Somatic inflammation; Sprague Dawley; Male
3.5
## Gould et al., 2016; Somatic inflammation; Sprague Dawley; Male
3.1
## Guimaraes et al., 2019; Neuropathy: Trauma Injury; Sprague
Dawley; Male              1.9
## Guimaraes et al., 2019; Neuropathy: Trauma Injury; Sprague
Dawley; Male              2.2
## Guimaraes et al., 2019; Neuropathy: Trauma Injury; Sprague
Dawley; Male              2.6
## Guimaraes et al., 2019; Neuropathy: Trauma Injury; Sprague
Dawley; Male              2.9
## Huang et al., 2013; Neuropathy: Trauma Injury; Wistar; Male
1.3
## Lau et al., 2013; Neuropathy: Trauma Injury; Sprague Dawley; Male
4.0
## Muralidharan et al., 2016; Neuropathy: Trauma Injury; Sprague
Dawley; Male          4.2
## Muralidharan et al., 2016; Somatic inflammation; Sprague Dawley;
Male                 3.4
## Rutten et al., 2018; Neuropathy: Trauma Injury; Wistar Hannover;
Male                 3.9
## Smith et al., 2016; Somatic inflammation; Wistar; Male
3.5
## Wodarski et al., 2016; Somatic inflammation; Sprague Dawley; Male
2.2
## Wodarski et al., 2016; Somatic inflammation; Sprague Dawley; Male
3.3
## Wodarski et al., 2016; Somatic inflammation; Sprague Dawley; Male
3.6
## Wodarski et al., 2016; Somatic inflammation; Wistar; Male
4.3
```

```
## Wodarski et al., 2016; Somatic inflammation; Wistar; Male
3.5
## Wodarski et al., 2016; Somatic inflammation; Wistar; Male
3.8
## Wodarski et al., 2016; Somatic inflammation; Wistar; Male
2.1
## Wodarski et al., 2016; Somatic inflammation; Wistar; Male
2.5
## Wodarski et al., 2016; Somatic inflammation; Wistar Hannover;
Male                3.6
## Wodarski et al., 2016; Somatic inflammation; Sprague Dawley; Male
3.9
## Wodarski et al., 2016; Somatic inflammation; Wistar Hannover;
Male                3.5
##
## Number of studies combined: k = 32
##
##                          SMD            95%-CI     t  p-value
## Random effects model -1.3372 [-1.7117; -0.9627] -7.28 < 0.0001
## Prediction interval          [-2.9751;  0.3006]
##
## Quantifying heterogeneity:
##  tau^2 = 0.6094 [0.2785; 1.5975]; tau = 0.7807 [0.5278; 1.2639]
##  I^2 = 63.2% [46.2%; 74.8%]; H = 1.65 [1.36; 1.99]
##
## Test of heterogeneity:
##      Q d.f.  p-value
##  84.14   31 < 0.0001
##
## Details on meta-analytical method:
## - Inverse variance method
## - Restricted maximum-likelihood estimator for tau^2
## - Q-profile method for confidence interval of tau^2 and tau
## - Hartung-Knapp adjustment for random effects model
```

```
# meta-analysis based on “excluding studies that reported burrowing as a primary outcome measure from those reporting it as a secondary outcome measure”
```

The sensitivity test based on “excluding studies that reported burrowing as a primary outcome measure from those reporting it as a secondary outcome measure” was not possible as only five studies declared such information.

```
# influence analysis
```

```
## Leave-One-Out Analysis (Sorted by I2)
##  ------------------------------------
##
Effect
## Omitting Smith et al., 2016; Somatic inflammation; Wistar; Male
-1.451
## Omitting Gould et al., 2016; Somatic inflammation; Sprague
Dawley; Male.2           -1.319
## Omitting Gould et al., 2016; Somatic inflammation; Sprague
Dawley; Male.1           -1.334
## Omitting Andrews et al., 2012; Somatic inflammation; Sprague
Dawley; NR               -1.345
## Omitting Andrews et al., 2012; Neuropathy: Trauma Injury; Wistar;
NR.1                -1.440
## Omitting Andrews et al., 2012; Neuropathy: Trauma Injury; Wistar;
NR                  -1.440
## Omitting Rutten et al., 2018; Neuropathy: Trauma Injury; Sprague
Dawley; Male       -1.337
## Omitting Guimaraes et al., 2019; Neuropathy: Trauma Injury;
Sprague Dawley; Male.1  -1.348
## Omitting Wodarski et al., 2016; Somatic inflammation; Sprague
Dawley; Male          -1.346
## Omitting Guimaraes et al., 2019; Neuropathy: Trauma Injury;
Sprague Dawley; Male.3  -1.431
## Omitting Wodarski et al., 2016; Somatic inflammation; Sprague
Dawley; Male.1        -1.367
## Omitting Guimaraes et al., 2019; Neuropathy: Trauma Injury;
Sprague Dawley; Male.2  -1.429
## Omitting Wodarski et al., 2016; Somatic inflammation; Wistar;
Male                  -1.426
## Omitting Wodarski et al., 2016; Somatic inflammation; Wistar
Hannover; Male         -1.426
## Omitting Wodarski et al., 2016; Somatic inflammation; Sprague
Dawley; Male.2        -1.422
## Omitting Wodarski et al., 2016; Somatic inflammation; Wistar
Hannover; Male.1       -1.381
## Omitting Gould et al., 2016; Somatic inflammation; Sprague
Dawley; Male             -1.379
## Omitting Gould et al., 2016; Somatic inflammation; Sprague
Dawley; Male.3           -1.387
## Omitting Gould et al., 2016; Somatic inflammation; Sprague
Dawley; Male.4           -1.384
## Omitting Wodarski et al., 2016; Somatic inflammation; Wistar;
Male.2                -1.421
## Omitting Muralidharan et al., 2016; Somatic inflammation; Sprague
Dawley; Male      -1.386
```

```
## Omitting Andrews et al., 2012; Neuropathy: Trauma Injury; Sprague
Dawley; NR        -1.387
## Omitting Deseure and Hans, 2018; Neuropathy: Trauma Injury;
Sprague Dawley; Male    -1.390
## Omitting Lau et al., 2013; Neuropathy: Trauma Injury; Sprague
Dawley; Male           -1.415
## Omitting Wodarski et al., 2016; Somatic inflammation; Wistar;
Male.3                 -1.386
## Omitting Guimaraes et al., 2019; Neuropathy: Trauma Injury;
Sprague Dawley; Male    -1.392
## Omitting Huang et al., 2013; Neuropathy: Trauma Injury; Wistar;
Male                   -1.387
## Omitting Muralidharan et al., 2016; Neuropathy: Trauma Injury;
Sprague Dawley; Male -1.399
## Omitting Gould et al., 2016; Somatic inflammation; Sprague
Dawley; Male.5            -1.403
## Omitting Rutten et al., 2018; Neuropathy: Trauma Injury; Wistar
Hannover; Male       -1.408
## Omitting Wodarski et al., 2016; Somatic inflammation; Wistar;
Male.1                 -1.402
## Omitting Wodarski et al., 2016; Somatic inflammation; Wistar;
Male.4                 -1.405
## Omitting Wodarski et al., 2016; Somatic inflammation; Sprague
Dawley; Male.3         -1.405
##
LLCI
## Omitting Smith et al., 2016; Somatic inflammation; Wistar; Male
-1.810
## Omitting Gould et al., 2016; Somatic inflammation; Sprague
Dawley; Male.2            -1.687
## Omitting Gould et al., 2016; Somatic inflammation; Sprague
Dawley; Male.1            -1.698
## Omitting Andrews et al., 2012; Somatic inflammation; Sprague
Dawley; NR                -1.732
## Omitting Andrews et al., 2012; Neuropathy: Trauma Injury; Wistar;
NR.1              -1.825
## Omitting Andrews et al., 2012; Neuropathy: Trauma Injury; Wistar;
NR                -1.825
## Omitting Rutten et al., 2018; Neuropathy: Trauma Injury; Sprague
Dawley; Male        -1.712
## Omitting Guimaraes et al., 2019; Neuropathy: Trauma Injury;
Sprague Dawley; Male.1  -1.727
## Omitting Wodarski et al., 2016; Somatic inflammation; Sprague
Dawley; Male           -1.726
## Omitting Guimaraes et al., 2019; Neuropathy: Trauma Injury;
Sprague Dawley; Male.3  -1.816
```

```
## Omitting Wodarski et al., 2016; Somatic inflammation; Sprague Dawley; Male.1         -1.759
## Omitting Guimaraes et al., 2019; Neuropathy: Trauma Injury; Sprague Dawley; Male.2  -1.814
## Omitting Wodarski et al., 2016; Somatic inflammation; Wistar; Male                  -1.820
## Omitting Wodarski et al., 2016; Somatic inflammation; Wistar Hannover; Male          -1.818
## Omitting Wodarski et al., 2016; Somatic inflammation; Sprague Dawley; Male.2         -1.816
## Omitting Wodarski et al., 2016; Somatic inflammation; Wistar Hannover; Male.1        -1.777
## Omitting Gould et al., 2016; Somatic inflammation; Sprague Dawley; Male               -1.773
## Omitting Gould et al., 2016; Somatic inflammation; Sprague Dawley; Male.3             -1.783
## Omitting Gould et al., 2016; Somatic inflammation; Sprague Dawley; Male.4             -1.780
## Omitting Wodarski et al., 2016; Somatic inflammation; Wistar; Male.2                -1.815
## Omitting Muralidharan et al., 2016; Somatic inflammation; Sprague Dawley; Male      -1.782
## Omitting Andrews et al., 2012; Neuropathy: Trauma Injury; Sprague Dawley; NR        -1.778
## Omitting Deseure and Hans, 2018; Neuropathy: Trauma Injury; Sprague Dawley; Male    -1.787
## Omitting Lau et al., 2013; Neuropathy: Trauma Injury; Sprague Dawley; Male           -1.811
## Omitting Wodarski et al., 2016; Somatic inflammation; Wistar; Male.3                -1.779
## Omitting Guimaraes et al., 2019; Neuropathy: Trauma Injury; Sprague Dawley; Male    -1.785
## Omitting Huang et al., 2013; Neuropathy: Trauma Injury; Wistar; Male                -1.778
## Omitting Muralidharan et al., 2016; Neuropathy: Trauma Injury; Sprague Dawley; Male -1.797
## Omitting Gould et al., 2016; Somatic inflammation; Sprague Dawley; Male.5             -1.800
## Omitting Rutten et al., 2018; Neuropathy: Trauma Injury; Wistar Hannover; Male      -1.805
## Omitting Wodarski et al., 2016; Somatic inflammation; Wistar; Male.1                -1.799
## Omitting Wodarski et al., 2016; Somatic inflammation; Wistar; Male.4                -1.800
## Omitting Wodarski et al., 2016; Somatic inflammation; Sprague Dawley; Male.3         -1.803
```

```
##
ULCI
## Omitting Smith et al., 2016; Somatic inflammation; Wistar; Male
-1.093
## Omitting Gould et al., 2016; Somatic inflammation; Sprague
Dawley; Male.2           -0.951
## Omitting Gould et al., 2016; Somatic inflammation; Sprague
Dawley; Male.1           -0.970
## Omitting Andrews et al., 2012; Somatic inflammation; Sprague
Dawley; NR             -0.959
## Omitting Andrews et al., 2012; Neuropathy: Trauma Injury; Wistar;
NR.1             -1.056
## Omitting Andrews et al., 2012; Neuropathy: Trauma Injury; Wistar;
NR               -1.055
## Omitting Rutten et al., 2018; Neuropathy: Trauma Injury; Sprague
Dawley; Male       -0.963
## Omitting Guimaraes et al., 2019; Neuropathy: Trauma Injury;
Sprague Dawley; Male.1  -0.969
## Omitting Wodarski et al., 2016; Somatic inflammation; Sprague
Dawley; Male          -0.967
## Omitting Guimaraes et al., 2019; Neuropathy: Trauma Injury;
Sprague Dawley; Male.3  -1.045
## Omitting Wodarski et al., 2016; Somatic inflammation; Sprague
Dawley; Male.1        -0.975
## Omitting Guimaraes et al., 2019; Neuropathy: Trauma Injury;
Sprague Dawley; Male.2  -1.043
## Omitting Wodarski et al., 2016; Somatic inflammation; Wistar;
Male                  -1.032
## Omitting Wodarski et al., 2016; Somatic inflammation; Wistar
Hannover; Male          -1.034
## Omitting Wodarski et al., 2016; Somatic inflammation; Sprague
Dawley; Male.2        -1.028
## Omitting Wodarski et al., 2016; Somatic inflammation; Wistar
Hannover; Male.1        -0.986
## Omitting Gould et al., 2016; Somatic inflammation; Sprague
Dawley; Male             -0.986
## Omitting Gould et al., 2016; Somatic inflammation; Sprague
Dawley; Male.3           -0.990
## Omitting Gould et al., 2016; Somatic inflammation; Sprague
Dawley; Male.4           -0.988
## Omitting Wodarski et al., 2016; Somatic inflammation; Wistar;
Male.2                -1.027
## Omitting Muralidharan et al., 2016; Somatic inflammation; Sprague
Dawley; Male      -0.990
## Omitting Andrews et al., 2012; Neuropathy: Trauma Injury; Sprague
Dawley; NR        -0.995
```

```
## Omitting Deseure and Hans, 2018; Neuropathy: Trauma Injury;
Sprague Dawley; Male    -0.994
## Omitting Lau et al., 2013; Neuropathy: Trauma Injury; Sprague
Dawley; Male           -1.018
## Omitting Wodarski et al., 2016; Somatic inflammation; Wistar;
Male.3                 -0.993
## Omitting Guimaraes et al., 2019; Neuropathy: Trauma Injury;
Sprague Dawley; Male    -0.998
## Omitting Huang et al., 2013; Neuropathy: Trauma Injury; Wistar;
Male                   -0.996
## Omitting Muralidharan et al., 2016; Neuropathy: Trauma Injury;
Sprague Dawley; Male -1.000
## Omitting Gould et al., 2016; Somatic inflammation; Sprague
Dawley; Male.5            -1.007
## Omitting Rutten et al., 2018; Neuropathy: Trauma Injury; Wistar
Hannover; Male      -1.011
## Omitting Wodarski et al., 2016; Somatic inflammation; Wistar;
Male.1                 -1.005
## Omitting Wodarski et al., 2016; Somatic inflammation; Wistar;
Male.4                 -1.010
## Omitting Wodarski et al., 2016; Somatic inflammation; Sprague
Dawley; Male.3         -1.007
##
I2
## Omitting Smith et al., 2016; Somatic inflammation; Wistar; Male
0.586
## Omitting Gould et al., 2016; Somatic inflammation; Sprague
Dawley; Male.2            0.614
## Omitting Gould et al., 2016; Somatic inflammation; Sprague
Dawley; Male.1            0.626
## Omitting Andrews et al., 2012; Somatic inflammation; Sprague
Dawley; NR              0.629
## Omitting Andrews et al., 2012; Neuropathy: Trauma Injury; Wistar;
NR.1                 0.631
## Omitting Andrews et al., 2012; Neuropathy: Trauma Injury; Wistar;
NR                   0.632
## Omitting Rutten et al., 2018; Neuropathy: Trauma Injury; Sprague
Dawley; Male        0.632
## Omitting Guimaraes et al., 2019; Neuropathy: Trauma Injury;
Sprague Dawley; Male.1  0.639
## Omitting Wodarski et al., 2016; Somatic inflammation; Sprague
Dawley; Male           0.639
## Omitting Guimaraes et al., 2019; Neuropathy: Trauma Injury;
Sprague Dawley; Male.3  0.648
## Omitting Wodarski et al., 2016; Somatic inflammation; Sprague
Dawley; Male.1         0.648
```

```
## Omitting Guimaraes et al., 2019; Neuropathy: Trauma Injury;
Sprague Dawley; Male.2  0.649
## Omitting Wodarski et al., 2016; Somatic inflammation; Wistar;
Male                  0.649
## Omitting Wodarski et al., 2016; Somatic inflammation; Wistar
Hannover; Male          0.652
## Omitting Wodarski et al., 2016; Somatic inflammation; Sprague
Dawley; Male.2        0.654
## Omitting Wodarski et al., 2016; Somatic inflammation; Wistar
Hannover; Male.1        0.654
## Omitting Gould et al., 2016; Somatic inflammation; Sprague
Dawley; Male                0.655
## Omitting Gould et al., 2016; Somatic inflammation; Sprague
Dawley; Male.3              0.655
## Omitting Gould et al., 2016; Somatic inflammation; Sprague
Dawley; Male.4              0.655
## Omitting Wodarski et al., 2016; Somatic inflammation; Wistar;
Male.2                0.655
## Omitting Muralidharan et al., 2016; Somatic inflammation; Sprague
Dawley; Male      0.656
## Omitting Andrews et al., 2012; Neuropathy: Trauma Injury; Sprague
Dawley; NR        0.657
## Omitting Deseure and Hans, 2018; Neuropathy: Trauma Injury;
Sprague Dawley; Male    0.657
## Omitting Lau et al., 2013; Neuropathy: Trauma Injury; Sprague
Dawley; Male          0.657
## Omitting Wodarski et al., 2016; Somatic inflammation; Wistar;
Male.3                0.657
## Omitting Guimaraes et al., 2019; Neuropathy: Trauma Injury;
Sprague Dawley; Male    0.658
## Omitting Huang et al., 2013; Neuropathy: Trauma Injury; Wistar;
Male                0.658
## Omitting Muralidharan et al., 2016; Neuropathy: Trauma Injury;
Sprague Dawley; Male 0.658
## Omitting Gould et al., 2016; Somatic inflammation; Sprague
Dawley; Male.5              0.659
## Omitting Rutten et al., 2018; Neuropathy: Trauma Injury; Wistar
Hannover; Male      0.659
## Omitting Wodarski et al., 2016; Somatic inflammation; Wistar;
Male.1                0.659
## Omitting Wodarski et al., 2016; Somatic inflammation; Wistar;
Male.4                0.659
## Omitting Wodarski et al., 2016; Somatic inflammation; Sprague
Dawley; Male.3        0.659
##
##
```

```
## Influence Diagnostics
##  -------------------
##
rstudent
## Omitting Andrews et al., 2012; Neuropathy: Trauma Injury; Wistar;
NR                    1.413
## Omitting Andrews et al., 2012; Neuropathy: Trauma Injury; Wistar;
NR.1                  1.451
## Omitting Andrews et al., 2012; Somatic inflammation; Sprague
Dawley; NR                 -1.274
## Omitting Andrews et al., 2012; Neuropathy: Trauma Injury; Sprague
Dawley; NR           -0.362
## Omitting Deseure and Hans, 2018; Neuropathy: Trauma Injury;
Sprague Dawley; Male      -0.199
## Omitting Gould et al., 2016; Somatic inflammation; Sprague
Dawley; Male                -0.523
## Omitting Gould et al., 2016; Somatic inflammation; Sprague
Dawley; Male.1              -2.195
## Omitting Gould et al., 2016; Somatic inflammation; Sprague
Dawley; Male.2              -2.113
## Omitting Gould et al., 2016; Somatic inflammation; Sprague
Dawley; Male.3              -0.298
## Omitting Gould et al., 2016; Somatic inflammation; Sprague
Dawley; Male.4              -0.374
## Omitting Gould et al., 2016; Somatic inflammation; Sprague
Dawley; Male.5               0.190
## Omitting Guimaraes et al., 2019; Neuropathy: Trauma Injury;
Sprague Dawley; Male      -0.151
## Omitting Guimaraes et al., 2019; Neuropathy: Trauma Injury;
Sprague Dawley; Male.1    -1.534
## Omitting Guimaraes et al., 2019; Neuropathy: Trauma Injury;
Sprague Dawley; Male.2     1.234
## Omitting Guimaraes et al., 2019; Neuropathy: Trauma Injury;
Sprague Dawley; Male.3     1.239
## Omitting Huang et al., 2013; Neuropathy: Trauma Injury; Wistar;
Male                  -0.362
## Omitting Lau et al., 2013; Neuropathy: Trauma Injury; Sprague
Dawley; Male             0.464
## Omitting Muralidharan et al., 2016; Neuropathy: Trauma Injury;
Sprague Dawley; Male    0.006
## Omitting Muralidharan et al., 2016; Somatic inflammation; Sprague
Dawley; Male        -0.311
## Omitting Rutten et al., 2018; Neuropathy: Trauma Injury; Sprague
Dawley; Male          -1.787
## Omitting Rutten et al., 2018; Neuropathy: Trauma Injury; Wistar
Hannover; Male          0.272
```

```
## Omitting Smith et al., 2016; Somatic inflammation; Wistar; Male 
2.567
## Omitting Wodarski et al., 2016; Somatic inflammation; Sprague 
Dawley; Male            -1.552
## Omitting Wodarski et al., 2016; Somatic inflammation; Sprague 
Dawley; Male.1          -0.816
## Omitting Wodarski et al., 2016; Somatic inflammation; Sprague 
Dawley; Male.2           0.738
## Omitting Wodarski et al., 2016; Somatic inflammation; Wistar; 
Male                     0.803
## Omitting Wodarski et al., 2016; Somatic inflammation; Wistar; 
Male.1                   0.120
## Omitting Wodarski et al., 2016; Somatic inflammation; Wistar; 
Male.2                   0.686
## Omitting Wodarski et al., 2016; Somatic inflammation; Wistar; 
Male.3                  -0.350
## Omitting Wodarski et al., 2016; Somatic inflammation; Wistar; 
Male.4                   0.282
## Omitting Wodarski et al., 2016; Somatic inflammation; Wistar 
Hannover; Male           0.889
## Omitting Wodarski et al., 2016; Somatic inflammation; Sprague 
Dawley; Male.3           0.187
## Omitting Wodarski et al., 2016; Somatic inflammation; Wistar 
Hannover; Male.1        -0.443
##
dffits
## Omitting Andrews et al., 2012; Neuropathy: Trauma Injury; Wistar; 
NR                 0.262
## Omitting Andrews et al., 2012; Neuropathy: Trauma Injury; Wistar; 
NR.1               0.266
## Omitting Andrews et al., 2012; Somatic inflammation; Sprague 
Dawley; NR             -0.268
## Omitting Andrews et al., 2012; Neuropathy: Trauma Injury; Sprague 
Dawley; NR        -0.036
## Omitting Deseure and Hans, 2018; Neuropathy: Trauma Injury; 
Sprague Dawley; Male    -0.015
## Omitting Gould et al., 2016; Somatic inflammation; Sprague 
Dawley; Male             -0.076
## Omitting Gould et al., 2016; Somatic inflammation; Sprague 
Dawley; Male.1           -0.334
## Omitting Gould et al., 2016; Somatic inflammation; Sprague 
Dawley; Male.2           -0.429
## Omitting Gould et al., 2016; Somatic inflammation; Sprague 
Dawley; Male.3           -0.035
## Omitting Gould et al., 2016; Somatic inflammation; Sprague 
Dawley; Male.4           -0.051
```

```
## Omitting Gould et al., 2016; Somatic inflammation; Sprague
Dawley; Male.5             0.056
## Omitting Guimaraes et al., 2019; Neuropathy: Trauma Injury;
Sprague Dawley; Male    -0.008
## Omitting Guimaraes et al., 2019; Neuropathy: Trauma Injury;
Sprague Dawley; Male.1  -0.253
## Omitting Guimaraes et al., 2019; Neuropathy: Trauma Injury;
Sprague Dawley; Male.2   0.196
## Omitting Guimaraes et al., 2019; Neuropathy: Trauma Injury;
Sprague Dawley; Male.3   0.206
## Omitting Huang et al., 2013; Neuropathy: Trauma Injury; Wistar;
Male                    -0.034
## Omitting Lau et al., 2013; Neuropathy: Trauma Injury; Sprague
Dawley; Male            0.115
## Omitting Muralidharan et al., 2016; Neuropathy: Trauma Injury;
Sprague Dawley; Male  0.030
## Omitting Muralidharan et al., 2016; Somatic inflammation; Sprague
Dawley; Male      -0.038
## Omitting Rutten et al., 2018; Neuropathy: Trauma Injury; Sprague
Dawley; Male       -0.315
## Omitting Rutten et al., 2018; Neuropathy: Trauma Injury; Wistar
Hannover; Male       0.079
## Omitting Smith et al., 2016; Somatic inflammation; Wistar; Male
0.355
## Omitting Wodarski et al., 2016; Somatic inflammation; Sprague
Dawley; Male           -0.261
## Omitting Wodarski et al., 2016; Somatic inflammation; Sprague
Dawley; Male.1         -0.145
## Omitting Wodarski et al., 2016; Somatic inflammation; Sprague
Dawley; Male.2          0.155
## Omitting Wodarski et al., 2016; Somatic inflammation; Wistar;
Male                    0.179
## Omitting Wodarski et al., 2016; Somatic inflammation; Wistar;
Male.1                  0.048
## Omitting Wodarski et al., 2016; Somatic inflammation; Wistar;
Male.2                  0.150
## Omitting Wodarski et al., 2016; Somatic inflammation; Wistar;
Male.3                 -0.039
## Omitting Wodarski et al., 2016; Somatic inflammation; Wistar;
Male.4                  0.064
## Omitting Wodarski et al., 2016; Somatic inflammation; Wistar
Hannover; Male           0.179
## Omitting Wodarski et al., 2016; Somatic inflammation; Sprague
Dawley; Male.3          0.063
## Omitting Wodarski et al., 2016; Somatic inflammation; Wistar
Hannover; Male.1        -0.065
```

```
##
cook.d
## Omitting Andrews et al., 2012; Neuropathy: Trauma Injury; Wistar;
NR                  0.066
## Omitting Andrews et al., 2012; Neuropathy: Trauma Injury; Wistar;
NR.1                0.068
## Omitting Andrews et al., 2012; Somatic inflammation; Sprague
Dawley; NR               0.069
## Omitting Andrews et al., 2012; Neuropathy: Trauma Injury; Sprague
Dawley; NR          0.001
## Omitting Deseure and Hans, 2018; Neuropathy: Trauma Injury;
Sprague Dawley; Male     0.000
## Omitting Gould et al., 2016; Somatic inflammation; Sprague
Dawley; Male                0.006
## Omitting Gould et al., 2016; Somatic inflammation; Sprague
Dawley; Male.1              0.107
## Omitting Gould et al., 2016; Somatic inflammation; Sprague
Dawley; Male.2              0.166
## Omitting Gould et al., 2016; Somatic inflammation; Sprague
Dawley; Male.3              0.001
## Omitting Gould et al., 2016; Somatic inflammation; Sprague
Dawley; Male.4              0.003
## Omitting Gould et al., 2016; Somatic inflammation; Sprague
Dawley; Male.5              0.003
## Omitting Guimaraes et al., 2019; Neuropathy: Trauma Injury;
Sprague Dawley; Male     0.000
## Omitting Guimaraes et al., 2019; Neuropathy: Trauma Injury;
Sprague Dawley; Male.1   0.062
## Omitting Guimaraes et al., 2019; Neuropathy: Trauma Injury;
Sprague Dawley; Male.2   0.038
## Omitting Guimaraes et al., 2019; Neuropathy: Trauma Injury;
Sprague Dawley; Male.3   0.042
## Omitting Huang et al., 2013; Neuropathy: Trauma Injury; Wistar;
Male                 0.001
## Omitting Lau et al., 2013; Neuropathy: Trauma Injury; Sprague
Dawley; Male           0.014
## Omitting Muralidharan et al., 2016; Neuropathy: Trauma Injury;
Sprague Dawley; Male  0.001
## Omitting Muralidharan et al., 2016; Somatic inflammation; Sprague
Dawley; Male       0.001
## Omitting Rutten et al., 2018; Neuropathy: Trauma Injury; Sprague
Dawley; Male        0.094
## Omitting Rutten et al., 2018; Neuropathy: Trauma Injury; Wistar
Hannover; Male       0.007
## Omitting Smith et al., 2016; Somatic inflammation; Wistar; Male
0.102
```

```
## Omitting Wodarski et al., 2016; Somatic inflammation; Sprague
Dawley; Male           0.066
## Omitting Wodarski et al., 2016; Somatic inflammation; Sprague
Dawley; Male.1         0.021
## Omitting Wodarski et al., 2016; Somatic inflammation; Sprague
Dawley; Male.2         0.025
## Omitting Wodarski et al., 2016; Somatic inflammation; Wistar;
Male                   0.033
## Omitting Wodarski et al., 2016; Somatic inflammation; Wistar;
Male.1                 0.002
## Omitting Wodarski et al., 2016; Somatic inflammation; Wistar;
Male.2                 0.023
## Omitting Wodarski et al., 2016; Somatic inflammation; Wistar;
Male.3                 0.002
## Omitting Wodarski et al., 2016; Somatic inflammation; Wistar;
Male.4                 0.004
## Omitting Wodarski et al., 2016; Somatic inflammation; Wistar
Hannover; Male          0.033
## Omitting Wodarski et al., 2016; Somatic inflammation; Sprague
Dawley; Male.3         0.004
## Omitting Wodarski et al., 2016; Somatic inflammation; Wistar
Hannover; Male.1        0.004
##
cov.r
## Omitting Andrews et al., 2012; Neuropathy: Trauma Injury; Wistar;
NR                1.006
## Omitting Andrews et al., 2012; Neuropathy: Trauma Injury; Wistar;
NR.1              1.002
## Omitting Andrews et al., 2012; Somatic inflammation; Sprague
Dawley; NR             0.998
## Omitting Andrews et al., 2012; Neuropathy: Trauma Injury; Sprague
Dawley; NR        1.029
## Omitting Deseure and Hans, 2018; Neuropathy: Trauma Injury;
Sprague Dawley; Male    1.077
## Omitting Gould et al., 2016; Somatic inflammation; Sprague
Dawley; Male             1.050
## Omitting Gould et al., 2016; Somatic inflammation; Sprague
Dawley; Male.1           0.932
## Omitting Gould et al., 2016; Somatic inflammation; Sprague
Dawley; Male.2           0.907
## Omitting Gould et al., 2016; Somatic inflammation; Sprague
Dawley; Male.3           1.075
## Omitting Gould et al., 2016; Somatic inflammation; Sprague
Dawley; Male.4           1.069
## Omitting Gould et al., 2016; Somatic inflammation; Sprague
Dawley; Male.5           1.071
```

```
## Omitting Guimaraes et al., 2019; Neuropathy: Trauma Injury;
Sprague Dawley; Male    1.043
## Omitting Guimaraes et al., 2019; Neuropathy: Trauma Injury;
Sprague Dawley; Male.1  0.977
## Omitting Guimaraes et al., 2019; Neuropathy: Trauma Injury;
Sprague Dawley; Male.2  1.020
## Omitting Guimaraes et al., 2019; Neuropathy: Trauma Injury;
Sprague Dawley; Male.3  1.021
## Omitting Huang et al., 2013; Neuropathy: Trauma Injury; Wistar;
Male                1.025
## Omitting Lau et al., 2013; Neuropathy: Trauma Injury; Sprague
Dawley; Male          1.082
## Omitting Muralidharan et al., 2016; Neuropathy: Trauma Injury;
Sprague Dawley; Male 1.094
## Omitting Muralidharan et al., 2016; Somatic inflammation; Sprague
Dawley; Male      1.070
## Omitting Rutten et al., 2018; Neuropathy: Trauma Injury; Sprague
Dawley; Male       0.953
## Omitting Rutten et al., 2018; Neuropathy: Trauma Injury; Wistar
Hannover; Male      1.086
## Omitting Smith et al., 2016; Somatic inflammation; Wistar; Male
0.854
## Omitting Wodarski et al., 2016; Somatic inflammation; Sprague
Dawley; Male          0.975
## Omitting Wodarski et al., 2016; Somatic inflammation; Sprague
Dawley; Male.1        1.039
## Omitting Wodarski et al., 2016; Somatic inflammation; Sprague
Dawley; Male.2        1.063
## Omitting Wodarski et al., 2016; Somatic inflammation; Wistar;
Male                  1.068
## Omitting Wodarski et al., 2016; Somatic inflammation; Wistar;
Male.1                1.081
## Omitting Wodarski et al., 2016; Somatic inflammation; Wistar;
Male.2                1.069
## Omitting Wodarski et al., 2016; Somatic inflammation; Wistar;
Male.3                1.042
## Omitting Wodarski et al., 2016; Somatic inflammation; Wistar;
Male.4                1.058
## Omitting Wodarski et al., 2016; Somatic inflammation; Wistar
Hannover; Male         1.054
## Omitting Wodarski et al., 2016; Somatic inflammation; Sprague
Dawley; Male.3        1.087
## Omitting Wodarski et al., 2016; Somatic inflammation; Wistar
Hannover; Male.1       1.065
##
QE.del
```

```
## Omitting Andrews et al., 2012; Neuropathy: Trauma Injury; Wistar;
NR                 84.152
## Omitting Andrews et al., 2012; Neuropathy: Trauma Injury; Wistar;
NR.1               84.063
## Omitting Andrews et al., 2012; Somatic inflammation; Sprague
Dawley; NR              83.523
## Omitting Andrews et al., 2012; Neuropathy: Trauma Injury; Sprague
Dawley; NR         90.510
## Omitting Deseure and Hans, 2018; Neuropathy: Trauma Injury;
Sprague Dawley; Male    90.325
## Omitting Gould et al., 2016; Somatic inflammation; Sprague
Dawley; Male              89.756
## Omitting Gould et al., 2016; Somatic inflammation; Sprague
Dawley; Male.1            82.972
## Omitting Gould et al., 2016; Somatic inflammation; Sprague
Dawley; Male.2            80.392
## Omitting Gould et al., 2016; Somatic inflammation; Sprague
Dawley; Male.3            89.976
## Omitting Gould et al., 2016; Somatic inflammation; Sprague
Dawley; Male.4            89.806
## Omitting Gould et al., 2016; Somatic inflammation; Sprague
Dawley; Male.5            90.835
## Omitting Guimaraes et al., 2019; Neuropathy: Trauma Injury;
Sprague Dawley; Male    90.702
## Omitting Guimaraes et al., 2019; Neuropathy: Trauma Injury;
Sprague Dawley; Male.1  85.986
## Omitting Guimaraes et al., 2019; Neuropathy: Trauma Injury;
Sprague Dawley; Male.2  88.399
## Omitting Guimaraes et al., 2019; Neuropathy: Trauma Injury;
Sprague Dawley; Male.3  88.114
## Omitting Huang et al., 2013; Neuropathy: Trauma Injury; Wistar;
Male                    90.539
## Omitting Lau et al., 2013; Neuropathy: Trauma Injury; Sprague
Dawley; Male           90.462
## Omitting Muralidharan et al., 2016; Neuropathy: Trauma Injury;
Sprague Dawley; Male 90.612
## Omitting Muralidharan et al., 2016; Somatic inflammation; Sprague
Dawley; Male       90.060
## Omitting Rutten et al., 2018; Neuropathy: Trauma Injury; Sprague
Dawley; Male        84.142
## Omitting Rutten et al., 2018; Neuropathy: Trauma Injury; Wistar
Hannover; Male       90.802
## Omitting Smith et al., 2016; Somatic inflammation; Wistar; Male
74.859
## Omitting Wodarski et al., 2016; Somatic inflammation; Sprague
Dawley; Male           85.769
```

```
## Omitting Wodarski et al., 2016; Somatic inflammation; Sprague
Dawley; Male.1         87.976
## Omitting Wodarski et al., 2016; Somatic inflammation; Sprague
Dawley; Male.2         89.701
## Omitting Wodarski et al., 2016; Somatic inflammation; Wistar;
Male                   88.414
## Omitting Wodarski et al., 2016; Somatic inflammation; Wistar;
Male.1                 90.824
## Omitting Wodarski et al., 2016; Somatic inflammation; Wistar;
Male.2                 89.794
## Omitting Wodarski et al., 2016; Somatic inflammation; Wistar;
Male.3                 90.420
## Omitting Wodarski et al., 2016; Somatic inflammation; Wistar;
Male.4                 90.801
## Omitting Wodarski et al., 2016; Somatic inflammation; Wistar
Hannover; Male          88.989
## Omitting Wodarski et al., 2016; Somatic inflammation; Sprague
Dawley; Male.3         90.836
## Omitting Wodarski et al., 2016; Somatic inflammation; Wistar
Hannover; Male.1        89.564
##
hat
## Omitting Andrews et al., 2012; Neuropathy: Trauma Injury; Wistar;
NR                 0.038
## Omitting Andrews et al., 2012; Neuropathy: Trauma Injury; Wistar;
NR.1               0.038
## Omitting Andrews et al., 2012; Somatic inflammation; Sprague
Dawley; NR              0.035
## Omitting Andrews et al., 2012; Neuropathy: Trauma Injury; Sprague
Dawley; NR         0.015
## Omitting Deseure and Hans, 2018; Neuropathy: Trauma Injury;
Sprague Dawley; Male    0.035
## Omitting Gould et al., 2016; Somatic inflammation; Sprague
Dawley; Male              0.028
## Omitting Gould et al., 2016; Somatic inflammation; Sprague
Dawley; Male.1            0.017
## Omitting Gould et al., 2016; Somatic inflammation; Sprague
Dawley; Male.2            0.027
## Omitting Gould et al., 2016; Somatic inflammation; Sprague
Dawley; Male.3            0.035
## Omitting Gould et al., 2016; Somatic inflammation; Sprague
Dawley; Male.4            0.034
## Omitting Gould et al., 2016; Somatic inflammation; Sprague
Dawley; Male.5            0.030
## Omitting Guimaraes et al., 2019; Neuropathy: Trauma Injury;
Sprague Dawley; Male    0.019
```

```
## Omitting Guimaraes et al., 2019; Neuropathy: Trauma Injury;
Sprague Dawley; Male.1  0.022
## Omitting Guimaraes et al., 2019; Neuropathy: Trauma Injury;
Sprague Dawley; Male.2  0.026
## Omitting Guimaraes et al., 2019; Neuropathy: Trauma Injury;
Sprague Dawley; Male.3  0.028
## Omitting Huang et al., 2013; Neuropathy: Trauma Injury; Wistar;
Male                0.013
## Omitting Lau et al., 2013; Neuropathy: Trauma Injury; Sprague
Dawley; Male            0.038
## Omitting Muralidharan et al., 2016; Neuropathy: Trauma Injury;
Sprague Dawley; Male 0.040
## Omitting Muralidharan et al., 2016; Somatic inflammation; Sprague
Dawley; Male      0.033
## Omitting Rutten et al., 2018; Neuropathy: Trauma Injury; Sprague
Dawley; Male       0.023
## Omitting Rutten et al., 2018; Neuropathy: Trauma Injury; Wistar
Hannover; Male      0.037
## Omitting Smith et al., 2016; Somatic inflammation; Wistar; Male
0.035
## Omitting Wodarski et al., 2016; Somatic inflammation; Sprague
Dawley; Male            0.022
## Omitting Wodarski et al., 2016; Somatic inflammation; Sprague
Dawley; Male.1          0.032
## Omitting Wodarski et al., 2016; Somatic inflammation; Sprague
Dawley; Male.2          0.035
## Omitting Wodarski et al., 2016; Somatic inflammation; Wistar;
Male                    0.041
## Omitting Wodarski et al., 2016; Somatic inflammation; Wistar;
Male.1                  0.035
## Omitting Wodarski et al., 2016; Somatic inflammation; Wistar;
Male.2                  0.036
## Omitting Wodarski et al., 2016; Somatic inflammation; Wistar;
Male.3                  0.021
## Omitting Wodarski et al., 2016; Somatic inflammation; Wistar;
Male.4                  0.025
## Omitting Wodarski et al., 2016; Somatic inflammation; Wistar
Hannover; Male           0.035
## Omitting Wodarski et al., 2016; Somatic inflammation; Sprague
Dawley; Male.3          0.038
## Omitting Wodarski et al., 2016; Somatic inflammation; Wistar
Hannover; Male.1         0.034
##
weight
## Omitting Andrews et al., 2012; Neuropathy: Trauma Injury; Wistar;
NR                 3.804
```

```
## Omitting Andrews et al., 2012; Neuropathy: Trauma Injury; Wistar;
NR.1               3.757
## Omitting Andrews et al., 2012; Somatic inflammation; Sprague
Dawley; NR               3.523
## Omitting Andrews et al., 2012; Neuropathy: Trauma Injury; Sprague
Dawley; NR         1.468
## Omitting Deseure and Hans, 2018; Neuropathy: Trauma Injury;
Sprague Dawley; Male     3.456
## Omitting Gould et al., 2016; Somatic inflammation; Sprague
Dawley; Male               2.760
## Omitting Gould et al., 2016; Somatic inflammation; Sprague
Dawley; Male.1             1.729
## Omitting Gould et al., 2016; Somatic inflammation; Sprague
Dawley; Male.2             2.734
## Omitting Gould et al., 2016; Somatic inflammation; Sprague
Dawley; Male.3             3.524
## Omitting Gould et al., 2016; Somatic inflammation; Sprague
Dawley; Male.4             3.392
## Omitting Gould et al., 2016; Somatic inflammation; Sprague
Dawley; Male.5             3.021
## Omitting Guimaraes et al., 2019; Neuropathy: Trauma Injury;
Sprague Dawley; Male     1.941
## Omitting Guimaraes et al., 2019; Neuropathy: Trauma Injury;
Sprague Dawley; Male.1   2.173
## Omitting Guimaraes et al., 2019; Neuropathy: Trauma Injury;
Sprague Dawley; Male.2   2.567
## Omitting Guimaraes et al., 2019; Neuropathy: Trauma Injury;
Sprague Dawley; Male.3   2.822
## Omitting Huang et al., 2013; Neuropathy: Trauma Injury; Wistar;
Male                 1.281
## Omitting Lau et al., 2013; Neuropathy: Trauma Injury; Sprague
Dawley; Male           3.811
## Omitting Muralidharan et al., 2016; Neuropathy: Trauma Injury;
Sprague Dawley; Male  4.045
## Omitting Muralidharan et al., 2016; Somatic inflammation; Sprague
Dawley; Male       3.339
## Omitting Rutten et al., 2018; Neuropathy: Trauma Injury; Sprague
Dawley; Male        2.326
## Omitting Rutten et al., 2018; Neuropathy: Trauma Injury; Wistar
Hannover; Male       3.750
## Omitting Smith et al., 2016; Somatic inflammation; Wistar; Male
3.451
## Omitting Wodarski et al., 2016; Somatic inflammation; Sprague
Dawley; Male           2.234
## Omitting Wodarski et al., 2016; Somatic inflammation; Sprague
Dawley; Male.1         3.211
```

```
## Omitting Wodarski et al., 2016; Somatic inflammation; Sprague
Dawley; Male.2         3.492
## Omitting Wodarski et al., 2016; Somatic inflammation; Wistar;
Male                   4.076
## Omitting Wodarski et al., 2016; Somatic inflammation; Wistar;
Male.1                 3.451
## Omitting Wodarski et al., 2016; Somatic inflammation; Wistar;
Male.2                 3.644
## Omitting Wodarski et al., 2016; Somatic inflammation; Wistar;
Male.3                 2.078
## Omitting Wodarski et al., 2016; Somatic inflammation; Wistar;
Male.4                 2.508
## Omitting Wodarski et al., 2016; Somatic inflammation; Wistar
Hannover; Male          3.518
## Omitting Wodarski et al., 2016; Somatic inflammation; Sprague
Dawley; Male.3         3.750
## Omitting Wodarski et al., 2016; Somatic inflammation; Wistar
Hannover; Male.1        3.365
##
infl
## Omitting Andrews et al., 2012; Neuropathy: Trauma Injury; Wistar;
NR
## Omitting Andrews et al., 2012; Neuropathy: Trauma Injury; Wistar;
NR.1
## Omitting Andrews et al., 2012; Somatic inflammation; Sprague
Dawley; NR
## Omitting Andrews et al., 2012; Neuropathy: Trauma Injury; Sprague
Dawley; NR
## Omitting Deseure and Hans, 2018; Neuropathy: Trauma Injury;
Sprague Dawley; Male
## Omitting Gould et al., 2016; Somatic inflammation; Sprague
Dawley; Male
## Omitting Gould et al., 2016; Somatic inflammation; Sprague
Dawley; Male.1
## Omitting Gould et al., 2016; Somatic inflammation; Sprague
Dawley; Male.2
## Omitting Gould et al., 2016; Somatic inflammation; Sprague
Dawley; Male.3
## Omitting Gould et al., 2016; Somatic inflammation; Sprague
Dawley; Male.4
## Omitting Gould et al., 2016; Somatic inflammation; Sprague
Dawley; Male.5
## Omitting Guimaraes et al., 2019; Neuropathy: Trauma Injury;
Sprague Dawley; Male
## Omitting Guimaraes et al., 2019; Neuropathy: Trauma Injury;
Sprague Dawley; Male.1
```

```
## Omitting Guimaraes et al., 2019; Neuropathy: Trauma Injury;
Sprague Dawley; Male.2
## Omitting Guimaraes et al., 2019; Neuropathy: Trauma Injury;
Sprague Dawley; Male.3
## Omitting Huang et al., 2013; Neuropathy: Trauma Injury; Wistar;
Male
## Omitting Lau et al., 2013; Neuropathy: Trauma Injury; Sprague
Dawley; Male
## Omitting Muralidharan et al., 2016; Neuropathy: Trauma Injury;
Sprague Dawley; Male
## Omitting Muralidharan et al., 2016; Somatic inflammation; Sprague
Dawley; Male
## Omitting Rutten et al., 2018; Neuropathy: Trauma Injury; Sprague
Dawley; Male
## Omitting Rutten et al., 2018; Neuropathy: Trauma Injury; Wistar
Hannover; Male
## Omitting Smith et al., 2016; Somatic inflammation; Wistar; Male
## Omitting Wodarski et al., 2016; Somatic inflammation; Sprague
Dawley; Male
## Omitting Wodarski et al., 2016; Somatic inflammation; Sprague
Dawley; Male.1
## Omitting Wodarski et al., 2016; Somatic inflammation; Sprague
Dawley; Male.2
## Omitting Wodarski et al., 2016; Somatic inflammation; Wistar;
Male
## Omitting Wodarski et al., 2016; Somatic inflammation; Wistar;
Male.1
## Omitting Wodarski et al., 2016; Somatic inflammation; Wistar;
Male.2
## Omitting Wodarski et al., 2016; Somatic inflammation; Wistar;
Male.3
## Omitting Wodarski et al., 2016; Somatic inflammation; Wistar;
Male.4
## Omitting Wodarski et al., 2016; Somatic inflammation; Wistar
Hannover; Male
## Omitting Wodarski et al., 2016; Somatic inflammation; Sprague
Dawley; Male.3
## Omitting Wodarski et al., 2016; Somatic inflammation; Wistar
Hannover; Male.1
##
##
## Baujat Diagnostics (sorted by Heterogeneity Contribution)
## -------------------------------------------------------
##
HetContrib
## Omitting Smith et al., 2016; Somatic inflammation; Wistar; Male
```

```
15.419
## Omitting Gould et al., 2016; Somatic inflammation; Sprague Dawley; Male.2                10.252
## Omitting Gould et al., 2016; Somatic inflammation; Sprague Dawley; Male.1                 7.802
## Omitting Andrews et al., 2012; Somatic inflammation; Sprague Dawley; NR                 7.039
## Omitting Rutten et al., 2018; Neuropathy: Trauma Injury; Sprague Dawley; Male            6.607
## Omitting Andrews et al., 2012; Neuropathy: Trauma Injury; Wistar; NR.1                   6.445
## Omitting Andrews et al., 2012; Neuropathy: Trauma Injury; Wistar; NR                     6.342
## Omitting Wodarski et al., 2016; Somatic inflammation; Sprague Dawley; Male              5.005
## Omitting Guimaraes et al., 2019; Neuropathy: Trauma Injury; Sprague Dawley; Male.1       4.794
## Omitting Wodarski et al., 2016; Somatic inflammation; Sprague Dawley; Male.1            2.781
## Omitting Guimaraes et al., 2019; Neuropathy: Trauma Injury; Sprague Dawley; Male.3       2.668
## Omitting Guimaraes et al., 2019; Neuropathy: Trauma Injury; Sprague Dawley; Male.2       2.398
## Omitting Wodarski et al., 2016; Somatic inflammation; Wistar; Male                      2.244
## Omitting Wodarski et al., 2016; Somatic inflammation; Wistar Hannover; Male              1.778
## Omitting Wodarski et al., 2016; Somatic inflammation; Wistar Hannover; Male.1            1.231
## Omitting Wodarski et al., 2016; Somatic inflammation; Sprague Dawley; Male.2            1.094
## Omitting Gould et al., 2016; Somatic inflammation; Sprague Dawley; Male                    1.060
## Omitting Wodarski et al., 2016; Somatic inflammation; Wistar; Male.2                    0.998
## Omitting Gould et al., 2016; Somatic inflammation; Sprague Dawley; Male.4                  0.997
## Omitting Gould et al., 2016; Somatic inflammation; Sprague Dawley; Male.3                  0.828
## Omitting Muralidharan et al., 2016; Somatic inflammation; Sprague Dawley; Male            0.752
## Omitting Deseure and Hans, 2018; Neuropathy: Trauma Injury; Sprague Dawley; Male         0.493
## Omitting Wodarski et al., 2016; Somatic inflammation; Wistar; Male.3                    0.412
## Omitting Lau et al., 2013; Neuropathy: Trauma Injury; Sprague
```

```
Dawley; Male               0.355
## Omitting Andrews et al., 2012; Neuropathy: Trauma Injury; Sprague
Dawley; NR              0.324
## Omitting Huang et al., 2013; Neuropathy: Trauma Injury; Wistar;
Male                        0.296
## Omitting Muralidharan et al., 2016; Neuropathy: Trauma Injury;
Sprague Dawley; Male      0.209
## Omitting Guimaraes et al., 2019; Neuropathy: Trauma Injury;
Sprague Dawley; Male         0.133
## Omitting Wodarski et al., 2016; Somatic inflammation; Wistar;
Male.4                       0.035
## Omitting Rutten et al., 2018; Neuropathy: Trauma Injury; Wistar
Hannover; Male             0.032
## Omitting Wodarski et al., 2016; Somatic inflammation; Wistar;
Male.1                       0.012
## Omitting Gould et al., 2016; Somatic inflammation; Sprague
Dawley; Male.5                  0.001
## Omitting Wodarski et al., 2016; Somatic inflammation; Sprague
Dawley; Male.3               0.000
##
InfluenceEffectSize
## Omitting Smith et al., 2016; Somatic inflammation; Wistar; Male
0.558
## Omitting Gould et al., 2016; Somatic inflammation; Sprague
Dawley; Male.2                           0.192
## Omitting Gould et al., 2016; Somatic inflammation; Sprague
Dawley; Male.1                           0.062
## Omitting Andrews et al., 2012; Somatic inflammation; Sprague
Dawley; NR                             0.275
## Omitting Rutten et al., 2018; Neuropathy: Trauma Injury; Sprague
Dawley; Male                         0.088
## Omitting Andrews et al., 2012; Neuropathy: Trauma Injury; Wistar;
NR.1                                0.328
## Omitting Andrews et al., 2012; Neuropathy: Trauma Injury; Wistar;
NR                                  0.342
## Omitting Wodarski et al., 2016; Somatic inflammation; Sprague
Dawley; Male                            0.062
## Omitting Guimaraes et al., 2019; Neuropathy: Trauma Injury;
Sprague Dawley; Male.1                   0.056
## Omitting Wodarski et al., 2016; Somatic inflammation; Sprague
Dawley; Male.1                          0.080
## Omitting Guimaraes et al., 2019; Neuropathy: Trauma Injury;
Sprague Dawley; Male.3                   0.054
## Omitting Guimaraes et al., 2019; Neuropathy: Trauma Injury;
Sprague Dawley; Male.2                   0.039
## Omitting Wodarski et al., 2016; Somatic inflammation; Wistar;
```

```
Male                                    0.178
## Omitting Wodarski et al., 2016; Somatic inflammation; Wistar
Hannover; Male                           0.069
## Omitting Wodarski et al., 2016; Somatic inflammation; Wistar
Hannover; Male.1                         0.041
## Omitting Wodarski et al., 2016; Somatic inflammation; Sprague
Dawley; Male.2                          0.041
## Omitting Gould et al., 2016; Somatic inflammation; Sprague
Dawley; Male                               0.020
## Omitting Wodarski et al., 2016; Somatic inflammation; Wistar;
Male.2                                  0.044
## Omitting Gould et al., 2016; Somatic inflammation; Sprague
Dawley; Male.4                             0.034
## Omitting Gould et al., 2016; Somatic inflammation; Sprague
Dawley; Male.3                             0.032
## Omitting Muralidharan et al., 2016; Somatic inflammation; Sprague
Dawley; Male                        0.024
## Omitting Deseure and Hans, 2018; Neuropathy: Trauma Injury;
Sprague Dawley; Male                      0.018
## Omitting Wodarski et al., 2016; Somatic inflammation; Wistar;
Male.3                                  0.004
## Omitting Lau et al., 2013; Neuropathy: Trauma Injury; Sprague
Dawley; Male                            0.019
## Omitting Andrews et al., 2012; Neuropathy: Trauma Injury; Sprague
Dawley; NR                          0.002
## Omitting Huang et al., 2013; Neuropathy: Trauma Injury; Wistar;
Male                                  0.002
## Omitting Muralidharan et al., 2016; Neuropathy: Trauma Injury;
Sprague Dawley; Male                    0.016
## Omitting Guimaraes et al., 2019; Neuropathy: Trauma Injury;
Sprague Dawley; Male                      0.001
## Omitting Wodarski et al., 2016; Somatic inflammation; Wistar;
Male.4                                  0.001
## Omitting Rutten et al., 2018; Neuropathy: Trauma Injury; Wistar
Hannover; Male                        0.002
## Omitting Wodarski et al., 2016; Somatic inflammation; Wistar;
Male.1                                  0.000
## Omitting Gould et al., 2016; Somatic inflammation; Sprague
Dawley; Male.5                             0.000
## Omitting Wodarski et al., 2016; Somatic inflammation; Sprague
Dawley; Male.3                          0.000
```

S4

Table. The number of cohort-level comparisons and animals for study design characteristics used in rat intervention experiments.

	No. of studies	No. of reports	No. of cohort-level comparisons	No. of animals
**Disease Model**				
Arthropathy	3	4	35	431
Somatic inflammation	2	2	21	320
Neuropathy: Diabetic-induced	1	2	12	213
Neuropathy: Trauma injury	2	2	10	177
Spinal cord injury	1	1	6	154
Mucositis	1	1	4	40
Procedure associated pain	1	1	1	16
**Drug Class**				
Gabapentinoid	6	8	25	403
NSAID	5	6	22	313
Opioid	4	5	22	288
Unknown mechanism of action	2	2	8	154
GABA agonist	1	1	3	44
Fatty acid amide hydrolase inhibitor	1	1	3	33
Nerve growth factor antibody	2	2	2	39
Dual amylin and calcitonin receptor agonist	1	1	1	13
Sodium channel blocker	1	1	1	26
TRPV1 antagonist	1	1	1	16
Combined therapy	1	1	1	22
**Rat Strain**				
Sprague Dawley	5	6	48	728
Wistar Hannover	1	1	17	191
Zucker diabetic fatty obese	1	1	10	174

Wistar	2	2	7	170
DA/Arc	1	1	4	40
Lewis	1	1	3	48
**Sex**				
Male	8	9	72	1089
Not reported	1	1	10	174
Female	2	2	7	88
**Substrate Type**				
Sand	3	3	47	668
Gravel	7	8	41	667
Food pellet	1	1	1	16
**Burrowing Outcome**				
Amount displaced	11	12	89	1351

S5

Burrowing Intervention Sensitivity

```
# meta-analysis
##
SMD
## Andrews et al., 2012; Ibuprofen; Somatic inflammation
1.5341
## Bryden et al., 2015; Celecoxib; Arthropathy
0.3582
## Bryden et al., 2015; Celecoxib; Arthropathy
0.7936
## Bryden et al., 2015; Celecoxib; Arthropathy
0.6324
## Bryden et al., 2015; Gabapentin ; Arthropathy                    -
0.2898
## Bryden et al., 2015; Gabapentin; Arthropathy                     -
0.0327
## Bryden et al., 2015; Gabapentin; Arthropathy                     -
0.0367
## Bryden et al., 2015; Ibuprofen; Arthropathy
0.5166
## Bryden et al., 2015; Ibuprofen; Arthropathy
0.4280
## Bryden et al., 2015; Ibuprofen; Arthropathy
0.2447
## Bryden et al., 2015; Morphine; Arthropathy
0.6919
## Bryden et al., 2015; Morphine; Arthropathy
0.1699
## Bryden et al., 2015; Morphine; Arthropathy
0.7987
## Bryden et al., 2015; Morphine; Arthropathy
0.7781
## Georgieva et al., 2019; Pregabalin; Spinal cord injury
2.3549
## Georgieva et al., 2019; Pregabalin; Spinal cord injury
1.7483
## Gould et al., 2016; Celecoxib; Somatic inflammation
0.9191
## Gould et al., 2016; Celecoxib; Somatic inflammation
0.3905
## Gould et al., 2016; Celecoxib; Somatic inflammation              -
0.1064
## Gould et al., 2016; Gabapentin; Somatic inflammation             -
0.0927
```

```
## Gould et al., 2016; Gabapentin; Somatic inflammation          -
1.2407
## Gould et al., 2016; Gabapentin; Somatic inflammation          -
0.1670
## Gould et al., 2016; Ibuprofen; Somatic inflammation
1.8166
## Gould et al., 2016; Indomethacin; Somatic inflammation
0.4873
## Gould et al., 2016; Indomethacin; Somatic inflammation
0.3141
## Gould et al., 2016; Indomethacin; Somatic inflammation
0.3659
## Gould et al., 2016; Morphine; Somatic inflammation
0.4095
## Gould et al., 2016; Morphine; Somatic inflammation
0.8877
## Gould et al., 2016; Morphine; Somatic inflammation            -
0.1140
## Gould et al., 2016; Tramadol; Somatic inflammation
0.6187
## Gould et al., 2016; Tramadol; Somatic inflammation            -
0.5030
## Gould et al., 2016; Tramadol; Somatic inflammation            -
1.0887
## Katri et al., 2019; Naproxen; Arthropathy                     -
0.3977
## Lau et al., 2013; Pregabalin; Neuropathy: Trauma Injury
1.8393
## Rutten et al., 2014; Ibuprofen; Arthropathy
2.1467
## Rutten et al., 2014; Ibuprofen; Arthropathy
2.3016
## Rutten et al., 2014; Morphine; Arthropathy
1.4692
## Rutten et al., 2014; Morphine; Arthropathy
1.2586
## Rutten et al., 2014; Morphine; Arthropathy
1.1209
## Rutten et al., 2014; Naproxen; Arthropathy
2.0364
## Rutten et al., 2014; Naproxen; Arthropathy
0.9457
## Rutten et al., 2014; Naproxen; Arthropathy
0.4620
## Rutten et al., 2014; Naproxen; Arthropathy
1.0277
```

```
## Rutten et al., 2014; Naproxen; Arthropathy
1.5052
## Rutten et al., 2014; Pregabalin; Arthropathy
1.4343
## Rutten et al., 2014; Pregabalin; Arthropathy
0.4772
## Rutten et al., 2014; Pregabalin; Arthropathy
1.0427
## Rutten et al., 2014; Pregabalin; Arthropathy
1.4475
## Rutten et al., 2014; Pregabalin; Arthropathy
1.3090
## Rutten et al., 2018; Gabapentin; Neuropathy: Trauma Injury
0.9613
## Rutten et al., 2018; Gabapentin; Neuropathy: Trauma Injury
1.6818
## Rutten et al., 2018; Gabapentin; Neuropathy: Diabetic-induced -
0.3123
## Rutten et al., 2018; Gabapentin; Neuropathy: Diabetic-induced
0.1925
## Rutten et al., 2018; Morphine; Neuropathy: Trauma Injury
1.5008
## Rutten et al., 2018; Morphine; Neuropathy: Trauma Injury
0.8370
## Rutten et al., 2018; Morphine; Neuropathy: Trauma Injury
0.8095
## Rutten et al., 2018; Morphine; Neuropathy: Diabetic-induced
0.3395
## Rutten et al., 2018; Morphine; Neuropathy: Diabetic-induced   -
0.1899
## Rutten et al., 2018; Morphine; Neuropathy: Diabetic-induced
0.0847
## Rutten et al., 2018; Pregabalin; Neuropathy: Trauma Injury
1.7115
## Rutten et al., 2018; Pregabalin; Neuropathy: Trauma Injury
2.0890
## Rutten et al., 2018; Pregabalin; Neuropathy: Trauma Injury
1.1622
## Rutten et al., 2018; Pregabalin; Neuropathy: Diabetic-induced
0.2304
## Rutten et al., 2018; Pregabalin; Neuropathy: Diabetic-induced -
0.0117
## Rutten et al., 2018; Pregabalin; Neuropathy: Diabetic-induced -
0.1285
## Rutten et al., 2018; Pregabalin; Neuropathy: Diabetic-induced -
0.2959
```

```
## Rutten et al., 2018; Tramadol; Neuropathy: Diabetic-induced   -
4.4069
## Rutten et al., 2018; Tramadol; Neuropathy: Diabetic-induced   -
0.5167
## Rutten et al., 2018; Tramadol; Neuropathy: Diabetic-induced   -
4.6307
##
95%-CI
## Andrews et al., 2012; Ibuprofen; Somatic inflammation         [
0.4147;  2.6536]
## Bryden et al., 2015; Celecoxib; Arthropathy                   [-
1.0409;  1.7572]
## Bryden et al., 2015; Celecoxib; Arthropathy                   [-
0.6557;  2.2429]
## Bryden et al., 2015; Celecoxib; Arthropathy                   [-
0.7941;  2.0589]
## Bryden et al., 2015; Gabapentin ; Arthropathy                 [-
1.6843;  1.1047]
## Bryden et al., 2015; Gabapentin; Arthropathy                  [-
1.4187;  1.3534]
## Bryden et al., 2015; Gabapentin; Arthropathy                  [-
1.4227;  1.3494]
## Bryden et al., 2015; Ibuprofen; Arthropathy                   [-
0.8141;  1.8474]
## Bryden et al., 2015; Ibuprofen; Arthropathy                   [-
0.8953;  1.7512]
## Bryden et al., 2015; Ibuprofen; Arthropathy                   [-
1.0674;  1.5568]
## Bryden et al., 2015; Morphine; Arthropathy                    [-
0.9058;  2.2896]
## Bryden et al., 2015; Morphine; Arthropathy                    [-
1.3826;  1.7223]
## Bryden et al., 2015; Morphine; Arthropathy                    [-
0.8147;  2.4121]
## Bryden et al., 2015; Morphine; Arthropathy                    [-
0.8321;  2.3883]
## Georgieva et al., 2019; Pregabalin; Spinal cord injury        [
1.0988;  3.6110]
## Georgieva et al., 2019; Pregabalin; Spinal cord injury        [
0.6168;  2.8797]
## Gould et al., 2016; Celecoxib; Somatic inflammation           [-
0.3453;  2.1834]
## Gould et al., 2016; Celecoxib; Somatic inflammation           [-
0.8186;  1.5996]
## Gould et al., 2016; Celecoxib; Somatic inflammation           [-
1.2767;  1.0640]
```

```
## Gould et al., 2016; Gabapentin; Somatic inflammation          [-
1.4001;  1.2147]
## Gould et al., 2016; Gabapentin; Somatic inflammation          [-
2.6518;  0.1703]
## Gould et al., 2016; Gabapentin; Somatic inflammation          [-
1.4595;  1.1255]
## Gould et al., 2016; Ibuprofen; Somatic inflammation           [
0.6745;  2.9588]
## Gould et al., 2016; Indomethacin; Somatic inflammation        [-
0.5986;  1.5731]
## Gould et al., 2016; Indomethacin; Somatic inflammation        [-
0.7090;  1.3371]
## Gould et al., 2016; Indomethacin; Somatic inflammation        [-
0.6809;  1.4126]
## Gould et al., 2016; Morphine; Somatic inflammation            [-
0.8008;  1.6198]
## Gould et al., 2016; Morphine; Somatic inflammation            [-
0.3525;  2.1278]
## Gould et al., 2016; Morphine; Somatic inflammation            [-
1.3116;  1.0837]
## Gould et al., 2016; Tramadol; Somatic inflammation            [-
0.8061;  2.0435]
## Gould et al., 2016; Tramadol; Somatic inflammation            [-
1.9147;  0.9087]
## Gould et al., 2016; Tramadol; Somatic inflammation            [-
2.5918;  0.4143]
## Katri et al., 2019; Naproxen; Arthropathy                     [-
1.6503;  0.8548]
## Lau et al., 2013; Pregabalin; Neuropathy: Trauma Injury       [
0.8574;  2.8212]
## Rutten et al., 2014; Ibuprofen; Arthropathy                   [
0.6613;  3.6320]
## Rutten et al., 2014; Ibuprofen; Arthropathy                   [
0.7703;  3.8329]
## Rutten et al., 2014; Morphine; Arthropathy                    [-
0.0213;  2.9598]
## Rutten et al., 2014; Morphine; Arthropathy                    [-
0.1853;  2.7025]
## Rutten et al., 2014; Morphine; Arthropathy                    [-
0.2957;  2.5375]
## Rutten et al., 2014; Naproxen; Arthropathy                    [
0.5825;  3.4902]
## Rutten et al., 2014; Naproxen; Arthropathy                    [-
0.2627;  2.1540]
## Rutten et al., 2014; Naproxen; Arthropathy                    [-
0.8639;  1.7880]
```

```
## Rutten et al., 2014; Naproxen; Arthropathy                      [-
0.3719;  2.4274]
## Rutten et al., 2014; Naproxen; Arthropathy                      [
0.0061;  3.0043]
## Rutten et al., 2014; Pregabalin; Arthropathy                    [
0.1328;  2.7357]
## Rutten et al., 2014; Pregabalin; Arthropathy                    [-
0.6745;  1.6288]
## Rutten et al., 2014; Pregabalin; Arthropathy                    [-
0.3596;  2.4450]
## Rutten et al., 2014; Pregabalin; Arthropathy                    [-
0.0379;  2.9330]
## Rutten et al., 2014; Pregabalin; Arthropathy                    [-
0.1455;  2.7636]
## Rutten et al., 2018; Gabapentin; Neuropathy: Trauma Injury      [-
0.0286;  1.9512]
## Rutten et al., 2018; Gabapentin; Neuropathy: Trauma Injury      [
0.5682;  2.7953]
## Rutten et al., 2018; Gabapentin; Neuropathy: Diabetic-induced [-
1.2256;  0.6011]
## Rutten et al., 2018; Gabapentin; Neuropathy: Diabetic-induced [-
0.7171;  1.1020]
## Rutten et al., 2018; Morphine; Neuropathy: Trauma Injury        [
0.2933;  2.7084]
## Rutten et al., 2018; Morphine; Neuropathy: Trauma Injury        [-
0.2057;  1.8797]
## Rutten et al., 2018; Morphine; Neuropathy: Trauma Injury        [-
0.2772;  1.8962]
## Rutten et al., 2018; Morphine; Neuropathy: Diabetic-induced     [-
0.8511;  1.5301]
## Rutten et al., 2018; Morphine; Neuropathy: Diabetic-induced     [-
1.3745;  0.9947]
## Rutten et al., 2018; Morphine; Neuropathy: Diabetic-induced     [-
1.0977;  1.2672]
## Rutten et al., 2018; Pregabalin; Neuropathy: Trauma Injury      [
0.4562;  2.9668]
## Rutten et al., 2018; Pregabalin; Neuropathy: Trauma Injury      [
0.7381;  3.4399]
## Rutten et al., 2018; Pregabalin; Neuropathy: Trauma Injury      [
0.0213;  2.3031]
## Rutten et al., 2018; Pregabalin; Neuropathy: Diabetic-induced [-
0.6802;  1.1410]
## Rutten et al., 2018; Pregabalin; Neuropathy: Diabetic-induced [-
0.9190;  0.8956]
## Rutten et al., 2018; Pregabalin; Neuropathy: Diabetic-induced [-
1.0061;  0.7492]
```

```
## Rutten et al., 2018; Pregabalin; Neuropathy: Diabetic-induced [-
1.2026;  0.6108]
## Rutten et al., 2018; Tramadol; Neuropathy: Diabetic-induced   [-
6.6147; -2.1991]
## Rutten et al., 2018; Tramadol; Neuropathy: Diabetic-induced   [-
1.7186;  0.6853]
## Rutten et al., 2018; Tramadol; Neuropathy: Diabetic-induced   [-
6.9191; -2.3424]
##
%W(random)
## Andrews et al., 2012; Ibuprofen; Somatic inflammation
1.6
## Bryden et al., 2015; Celecoxib; Arthropathy
1.3
## Bryden et al., 2015; Celecoxib; Arthropathy
1.3
## Bryden et al., 2015; Celecoxib; Arthropathy
1.3
## Bryden et al., 2015; Gabapentin ; Arthropathy
1.3
## Bryden et al., 2015; Gabapentin; Arthropathy
1.3
## Bryden et al., 2015; Gabapentin; Arthropathy
1.3
## Bryden et al., 2015; Ibuprofen; Arthropathy
1.4
## Bryden et al., 2015; Ibuprofen; Arthropathy
1.4
## Bryden et al., 2015; Ibuprofen; Arthropathy
1.4
## Bryden et al., 2015; Morphine; Arthropathy
1.1
## Bryden et al., 2015; Morphine; Arthropathy
1.2
## Bryden et al., 2015; Morphine; Arthropathy
1.1
## Bryden et al., 2015; Morphine; Arthropathy
1.1
## Georgieva et al., 2019; Pregabalin; Spinal cord injury
1.5
## Georgieva et al., 2019; Pregabalin; Spinal cord injury
1.6
## Gould et al., 2016; Celecoxib; Somatic inflammation
1.5
## Gould et al., 2016; Celecoxib; Somatic inflammation
1.5
```

```
## Gould et al., 2016; Celecoxib; Somatic inflammation
1.6
## Gould et al., 2016; Gabapentin; Somatic inflammation
1.4
## Gould et al., 2016; Gabapentin; Somatic inflammation
1.3
## Gould et al., 2016; Gabapentin; Somatic inflammation
1.4
## Gould et al., 2016; Ibuprofen; Somatic inflammation
1.6
## Gould et al., 2016; Indomethacin; Somatic inflammation
1.7
## Gould et al., 2016; Indomethacin; Somatic inflammation
1.8
## Gould et al., 2016; Indomethacin; Somatic inflammation
1.7
## Gould et al., 2016; Morphine; Somatic inflammation
1.5
## Gould et al., 2016; Morphine; Somatic inflammation
1.5
## Gould et al., 2016; Morphine; Somatic inflammation
1.5
## Gould et al., 2016; Tramadol; Somatic inflammation
1.3
## Gould et al., 2016; Tramadol; Somatic inflammation
1.3
## Gould et al., 2016; Tramadol; Somatic inflammation
1.2
## Katri et al., 2019; Naproxen; Arthropathy
1.5
## Lau et al., 2013; Pregabalin; Neuropathy: Trauma Injury
1.8
## Rutten et al., 2014; Ibuprofen; Arthropathy
1.2
## Rutten et al., 2014; Ibuprofen; Arthropathy
1.2
## Rutten et al., 2014; Morphine; Arthropathy
1.2
## Rutten et al., 2014; Morphine; Arthropathy
1.3
## Rutten et al., 2014; Morphine; Arthropathy
1.3
## Rutten et al., 2014; Naproxen; Arthropathy
1.3
## Rutten et al., 2014; Naproxen; Arthropathy
1.5
```

```
## Rutten et al., 2014; Naproxen; Arthropathy
1.4
## Rutten et al., 2014; Naproxen; Arthropathy
1.3
## Rutten et al., 2014; Naproxen; Arthropathy
1.2
## Rutten et al., 2014; Pregabalin; Arthropathy
1.4
## Rutten et al., 2014; Pregabalin; Arthropathy
1.6
## Rutten et al., 2014; Pregabalin; Arthropathy
1.3
## Rutten et al., 2014; Pregabalin; Arthropathy
1.2
## Rutten et al., 2014; Pregabalin; Arthropathy
1.3
## Rutten et al., 2018; Gabapentin; Neuropathy: Trauma Injury
1.8
## Rutten et al., 2018; Gabapentin; Neuropathy: Trauma Injury
1.6
## Rutten et al., 2018; Gabapentin; Neuropathy: Diabetic-induced
1.9
## Rutten et al., 2018; Gabapentin; Neuropathy: Diabetic-induced
1.9
## Rutten et al., 2018; Morphine; Neuropathy: Trauma Injury
1.5
## Rutten et al., 2018; Morphine; Neuropathy: Trauma Injury
1.7
## Rutten et al., 2018; Morphine; Neuropathy: Trauma Injury
1.7
## Rutten et al., 2018; Morphine; Neuropathy: Diabetic-induced
1.5
## Rutten et al., 2018; Morphine; Neuropathy: Diabetic-induced
1.6
## Rutten et al., 2018; Morphine; Neuropathy: Diabetic-induced
1.6
## Rutten et al., 2018; Pregabalin; Neuropathy: Trauma Injury
1.5
## Rutten et al., 2018; Pregabalin; Neuropathy: Trauma Injury
1.4
## Rutten et al., 2018; Pregabalin; Neuropathy: Trauma Injury
1.6
## Rutten et al., 2018; Pregabalin; Neuropathy: Diabetic-induced
1.9
## Rutten et al., 2018; Pregabalin; Neuropathy: Diabetic-induced
1.9
```

```
## Rutten et al., 2018; Pregabalin; Neuropathy: Diabetic-induced
2.0
## Rutten et al., 2018; Pregabalin; Neuropathy: Diabetic-induced
1.9
## Rutten et al., 2018; Tramadol; Neuropathy: Diabetic-induced
0.7
## Rutten et al., 2018; Tramadol; Neuropathy: Diabetic-induced
1.5
## Rutten et al., 2018; Tramadol; Neuropathy: Diabetic-induced
0.7
##
## Number of studies combined: k = 69
##
##                         SMD            95%-CI    t  p-value
## Random effects model 0.5808 [ 0.3390; 0.8226] 4.79 < 0.0001
## Prediction interval         [-0.7118; 1.8734]
##
## Quantifying heterogeneity:
##  tau^2 = 0.4047 [0.3046; 1.2305]; tau = 0.6361 [0.5519; 1.1093]
##  I^2 = 53.8% [39.3%; 64.8%]; H = 1.47 [1.28; 1.69]
##
## Test of heterogeneity:
##       Q d.f.  p-value
##  147.15   68 < 0.0001
##
## Details on meta-analytical method:
## - Inverse variance method
## - Restricted maximum-likelihood estimator for tau^2
## - Q-profile method for confidence interval of tau^2 and tau
## - Hartung-Knapp adjustment for random effects model
```

```
# detecting outliers
```

```
##                                                    Author
lowerci  upperci
## 1  Georgieva et al., 2019; Pregabalin; Spinal cord injury
1.0987584 3.611014
## 2 Lau et al., 2013; Pregabalin; Neuropathy: Trauma Injury
0.8573724 2.821207
```

```
# meta-analysis with outliers removal ##
SMD
## Andrews et al., 2012; Ibuprofen; Somatic inflammation
1.5341
## Bryden et al., 2015; Celecoxib; Arthropathy
0.3582
## Bryden et al., 2015; Celecoxib; Arthropathy
0.7936
## Bryden et al., 2015; Celecoxib; Arthropathy
0.6324
## Bryden et al., 2015; Gabapentin ; Arthropathy                  -
0.2898
## Bryden et al., 2015; Gabapentin; Arthropathy                   -
0.0327
## Bryden et al., 2015; Gabapentin; Arthropathy                   -
0.0367
## Bryden et al., 2015; Ibuprofen; Arthropathy
0.5166
## Bryden et al., 2015; Ibuprofen; Arthropathy
0.4280
## Bryden et al., 2015; Ibuprofen; Arthropathy
0.2447
## Bryden et al., 2015; Morphine; Arthropathy
0.6919
## Bryden et al., 2015; Morphine; Arthropathy
0.1699
## Bryden et al., 2015; Morphine; Arthropathy
0.7987
## Bryden et al., 2015; Morphine; Arthropathy
0.7781
## Georgieva et al., 2019; Pregabalin; Spinal cord injury
1.7483
## Gould et al., 2016; Celecoxib; Somatic inflammation
0.9191
## Gould et al., 2016; Celecoxib; Somatic inflammation
0.3905
## Gould et al., 2016; Celecoxib; Somatic inflammation           -
0.1064
## Gould et al., 2016; Gabapentin; Somatic inflammation          -
0.0927
## Gould et al., 2016; Gabapentin; Somatic inflammation          -
1.2407
## Gould et al., 2016; Gabapentin; Somatic inflammation          -
0.1670
## Gould et al., 2016; Ibuprofen; Somatic inflammation
1.8166
```

```
## Gould et al., 2016; Indomethacin; Somatic inflammation
0.4873
## Gould et al., 2016; Indomethacin; Somatic inflammation
0.3141
## Gould et al., 2016; Indomethacin; Somatic inflammation
0.3659
## Gould et al., 2016; Morphine; Somatic inflammation
0.4095
## Gould et al., 2016; Morphine; Somatic inflammation
0.8877
## Gould et al., 2016; Morphine; Somatic inflammation          -
0.1140
## Gould et al., 2016; Tramadol; Somatic inflammation
0.6187
## Gould et al., 2016; Tramadol; Somatic inflammation          -
0.5030
## Gould et al., 2016; Tramadol; Somatic inflammation          -
1.0887
## Katri et al., 2019; Naproxen; Arthropathy                   -
0.3977
## Rutten et al., 2014; Ibuprofen; Arthropathy
2.1467
## Rutten et al., 2014; Ibuprofen; Arthropathy
2.3016
## Rutten et al., 2014; Morphine; Arthropathy
1.4692
## Rutten et al., 2014; Morphine; Arthropathy
1.2586
## Rutten et al., 2014; Morphine; Arthropathy
1.1209
## Rutten et al., 2014; Naproxen; Arthropathy
2.0364
## Rutten et al., 2014; Naproxen; Arthropathy
0.9457
## Rutten et al., 2014; Naproxen; Arthropathy
0.4620
## Rutten et al., 2014; Naproxen; Arthropathy
1.0277
## Rutten et al., 2014; Naproxen; Arthropathy
1.5052
## Rutten et al., 2014; Pregabalin; Arthropathy
1.4343
## Rutten et al., 2014; Pregabalin; Arthropathy
0.4772
## Rutten et al., 2014; Pregabalin; Arthropathy
1.0427
```

```
## Rutten et al., 2014; Pregabalin; Arthropathy
1.4475
## Rutten et al., 2014; Pregabalin; Arthropathy
1.3090
## Rutten et al., 2018; Gabapentin; Neuropathy: Trauma Injury
0.9613
## Rutten et al., 2018; Gabapentin; Neuropathy: Trauma Injury
1.6818
## Rutten et al., 2018; Gabapentin; Neuropathy: Diabetic-induced -
0.3123
## Rutten et al., 2018; Gabapentin; Neuropathy: Diabetic-induced
0.1925
## Rutten et al., 2018; Morphine; Neuropathy: Trauma Injury
1.5008
## Rutten et al., 2018; Morphine; Neuropathy: Trauma Injury
0.8370
## Rutten et al., 2018; Morphine; Neuropathy: Trauma Injury
0.8095
## Rutten et al., 2018; Morphine; Neuropathy: Diabetic-induced
0.3395
## Rutten et al., 2018; Morphine; Neuropathy: Diabetic-induced   -
0.1899
## Rutten et al., 2018; Morphine; Neuropathy: Diabetic-induced
0.0847
## Rutten et al., 2018; Pregabalin; Neuropathy: Trauma Injury
1.7115
## Rutten et al., 2018; Pregabalin; Neuropathy: Trauma Injury
2.0890
## Rutten et al., 2018; Pregabalin; Neuropathy: Trauma Injury
1.1622
## Rutten et al., 2018; Pregabalin; Neuropathy: Diabetic-induced
0.2304
## Rutten et al., 2018; Pregabalin; Neuropathy: Diabetic-induced -
0.0117
## Rutten et al., 2018; Pregabalin; Neuropathy: Diabetic-induced -
0.1285
## Rutten et al., 2018; Pregabalin; Neuropathy: Diabetic-induced -
0.2959
## Rutten et al., 2018; Tramadol; Neuropathy: Diabetic-induced   -
4.4069
## Rutten et al., 2018; Tramadol; Neuropathy: Diabetic-induced   -
0.5167
## Rutten et al., 2018; Tramadol; Neuropathy: Diabetic-induced   -
4.6307
##
95%-CI
```

```
## Andrews et al., 2012; Ibuprofen; Somatic inflammation         [
0.4147;  2.6536]
## Bryden et al., 2015; Celecoxib; Arthropathy                    [-
1.0409;  1.7572]
## Bryden et al., 2015; Celecoxib; Arthropathy                    [-
0.6557;  2.2429]
## Bryden et al., 2015; Celecoxib; Arthropathy                    [-
0.7941;  2.0589]
## Bryden et al., 2015; Gabapentin ; Arthropathy                  [-
1.6843;  1.1047]
## Bryden et al., 2015; Gabapentin; Arthropathy                   [-
1.4187;  1.3534]
## Bryden et al., 2015; Gabapentin; Arthropathy                   [-
1.4227;  1.3494]
## Bryden et al., 2015; Ibuprofen; Arthropathy                    [-
0.8141;  1.8474]
## Bryden et al., 2015; Ibuprofen; Arthropathy                    [-
0.8953;  1.7512]
## Bryden et al., 2015; Ibuprofen; Arthropathy                    [-
1.0674;  1.5568]
## Bryden et al., 2015; Morphine; Arthropathy                     [-
0.9058;  2.2896]
## Bryden et al., 2015; Morphine; Arthropathy                     [-
1.3826;  1.7223]
## Bryden et al., 2015; Morphine; Arthropathy                     [-
0.8147;  2.4121]
## Bryden et al., 2015; Morphine; Arthropathy                     [-
0.8321;  2.3883]
## Georgieva et al., 2019; Pregabalin; Spinal cord injury         [
0.6168;  2.8797]
## Gould et al., 2016; Celecoxib; Somatic inflammation            [-
0.3453;  2.1834]
## Gould et al., 2016; Celecoxib; Somatic inflammation            [-
0.8186;  1.5996]
## Gould et al., 2016; Celecoxib; Somatic inflammation            [-
1.2767;  1.0640]
## Gould et al., 2016; Gabapentin; Somatic inflammation           [-
1.4001;  1.2147]
## Gould et al., 2016; Gabapentin; Somatic inflammation           [-
2.6518;  0.1703]
## Gould et al., 2016; Gabapentin; Somatic inflammation           [-
1.4595;  1.1255]
## Gould et al., 2016; Ibuprofen; Somatic inflammation            [
0.6745;  2.9588]
## Gould et al., 2016; Indomethacin; Somatic inflammation         [-
0.5986;  1.5731]
```

```
## Gould et al., 2016; Indomethacin; Somatic inflammation          [-
0.7090;  1.3371]
## Gould et al., 2016; Indomethacin; Somatic inflammation          [-
0.6809;  1.4126]
## Gould et al., 2016; Morphine; Somatic inflammation              [-
0.8008;  1.6198]
## Gould et al., 2016; Morphine; Somatic inflammation              [-
0.3525;  2.1278]
## Gould et al., 2016; Morphine; Somatic inflammation              [-
1.3116;  1.0837]
## Gould et al., 2016; Tramadol; Somatic inflammation              [-
0.8061;  2.0435]
## Gould et al., 2016; Tramadol; Somatic inflammation              [-
1.9147;  0.9087]
## Gould et al., 2016; Tramadol; Somatic inflammation              [-
2.5918;  0.4143]
## Katri et al., 2019; Naproxen; Arthropathy                       [-
1.6503;  0.8548]
## Rutten et al., 2014; Ibuprofen; Arthropathy                     [
0.6613;  3.6320]
## Rutten et al., 2014; Ibuprofen; Arthropathy                     [
0.7703;  3.8329]
## Rutten et al., 2014; Morphine; Arthropathy                      [-
0.0213;  2.9598]
## Rutten et al., 2014; Morphine; Arthropathy                      [-
0.1853;  2.7025]
## Rutten et al., 2014; Morphine; Arthropathy                      [-
0.2957;  2.5375]
## Rutten et al., 2014; Naproxen; Arthropathy                      [
0.5825;  3.4902]
## Rutten et al., 2014; Naproxen; Arthropathy                      [-
0.2627;  2.1540]
## Rutten et al., 2014; Naproxen; Arthropathy                      [-
0.8639;  1.7880]
## Rutten et al., 2014; Naproxen; Arthropathy                      [-
0.3719;  2.4274]
## Rutten et al., 2014; Naproxen; Arthropathy                      [
0.0061;  3.0043]
## Rutten et al., 2014; Pregabalin; Arthropathy                    [
0.1328;  2.7357]
## Rutten et al., 2014; Pregabalin; Arthropathy                    [-
0.6745;  1.6288]
## Rutten et al., 2014; Pregabalin; Arthropathy                    [-
0.3596;  2.4450]
## Rutten et al., 2014; Pregabalin; Arthropathy                    [-
0.0379;  2.9330]
```

```
## Rutten et al., 2014; Pregabalin; Arthropathy                 [-
0.1455;  2.7636]
## Rutten et al., 2018; Gabapentin; Neuropathy: Trauma Injury    [-
0.0286;  1.9512]
## Rutten et al., 2018; Gabapentin; Neuropathy: Trauma Injury    [
0.5682;  2.7953]
## Rutten et al., 2018; Gabapentin; Neuropathy: Diabetic-induced [-
1.2256;  0.6011]
## Rutten et al., 2018; Gabapentin; Neuropathy: Diabetic-induced [-
0.7171;  1.1020]
## Rutten et al., 2018; Morphine; Neuropathy: Trauma Injury      [
0.2933;  2.7084]
## Rutten et al., 2018; Morphine; Neuropathy: Trauma Injury      [-
0.2057;  1.8797]
## Rutten et al., 2018; Morphine; Neuropathy: Trauma Injury      [-
0.2772;  1.8962]
## Rutten et al., 2018; Morphine; Neuropathy: Diabetic-induced   [-
0.8511;  1.5301]
## Rutten et al., 2018; Morphine; Neuropathy: Diabetic-induced   [-
1.3745;  0.9947]
## Rutten et al., 2018; Morphine; Neuropathy: Diabetic-induced   [-
1.0977;  1.2672]
## Rutten et al., 2018; Pregabalin; Neuropathy: Trauma Injury    [
0.4562;  2.9668]
## Rutten et al., 2018; Pregabalin; Neuropathy: Trauma Injury    [
0.7381;  3.4399]
## Rutten et al., 2018; Pregabalin; Neuropathy: Trauma Injury    [
0.0213;  2.3031]
## Rutten et al., 2018; Pregabalin; Neuropathy: Diabetic-induced [-
0.6802;  1.1410]
## Rutten et al., 2018; Pregabalin; Neuropathy: Diabetic-induced [-
0.9190;  0.8956]
## Rutten et al., 2018; Pregabalin; Neuropathy: Diabetic-induced [-
1.0061;  0.7492]
## Rutten et al., 2018; Pregabalin; Neuropathy: Diabetic-induced [-
1.2026;  0.6108]
## Rutten et al., 2018; Tramadol; Neuropathy: Diabetic-induced   [-
6.6147; -2.1991]
## Rutten et al., 2018; Tramadol; Neuropathy: Diabetic-induced   [-
1.7186;  0.6853]
## Rutten et al., 2018; Tramadol; Neuropathy: Diabetic-induced   [-
6.9191; -2.3424]
##
%W(random)
## Andrews et al., 2012; Ibuprofen; Somatic inflammation
1.7
```

```
## Bryden et al., 2015; Celecoxib; Arthropathy
1.3
## Bryden et al., 2015; Celecoxib; Arthropathy
1.3
## Bryden et al., 2015; Celecoxib; Arthropathy
1.3
## Bryden et al., 2015; Gabapentin ; Arthropathy
1.3
## Bryden et al., 2015; Gabapentin; Arthropathy
1.4
## Bryden et al., 2015; Gabapentin; Arthropathy
1.4
## Bryden et al., 2015; Ibuprofen; Arthropathy
1.4
## Bryden et al., 2015; Ibuprofen; Arthropathy
1.4
## Bryden et al., 2015; Ibuprofen; Arthropathy
1.4
## Bryden et al., 2015; Morphine; Arthropathy
1.1
## Bryden et al., 2015; Morphine; Arthropathy
1.2
## Bryden et al., 2015; Morphine; Arthropathy
1.1
## Bryden et al., 2015; Morphine; Arthropathy
1.1
## Georgieva et al., 2019; Pregabalin; Spinal cord injury
1.7
## Gould et al., 2016; Celecoxib; Somatic inflammation
1.5
## Gould et al., 2016; Celecoxib; Somatic inflammation
1.6
## Gould et al., 2016; Celecoxib; Somatic inflammation
1.6
## Gould et al., 2016; Gabapentin; Somatic inflammation
1.4
## Gould et al., 2016; Gabapentin; Somatic inflammation
1.3
## Gould et al., 2016; Gabapentin; Somatic inflammation
1.5
## Gould et al., 2016; Ibuprofen; Somatic inflammation
1.7
## Gould et al., 2016; Indomethacin; Somatic inflammation
1.8
## Gould et al., 2016; Indomethacin; Somatic inflammation
1.9
```

```
## Gould et al., 2016; Indomethacin; Somatic inflammation
1.8
## Gould et al., 2016; Morphine; Somatic inflammation
1.6
## Gould et al., 2016; Morphine; Somatic inflammation
1.5
## Gould et al., 2016; Morphine; Somatic inflammation
1.6
## Gould et al., 2016; Tramadol; Somatic inflammation
1.3
## Gould et al., 2016; Tramadol; Somatic inflammation
1.3
## Gould et al., 2016; Tramadol; Somatic inflammation
1.2
## Katri et al., 2019; Naproxen; Arthropathy
1.5
## Rutten et al., 2014; Ibuprofen; Arthropathy
1.2
## Rutten et al., 2014; Ibuprofen; Arthropathy
1.2
## Rutten et al., 2014; Morphine; Arthropathy
1.2
## Rutten et al., 2014; Morphine; Arthropathy
1.3
## Rutten et al., 2014; Morphine; Arthropathy
1.3
## Rutten et al., 2014; Naproxen; Arthropathy
1.3
## Rutten et al., 2014; Naproxen; Arthropathy
1.6
## Rutten et al., 2014; Naproxen; Arthropathy
1.4
## Rutten et al., 2014; Naproxen; Arthropathy
1.3
## Rutten et al., 2014; Naproxen; Arthropathy
1.2
## Rutten et al., 2014; Pregabalin; Arthropathy
1.5
## Rutten et al., 2014; Pregabalin; Arthropathy
1.7
## Rutten et al., 2014; Pregabalin; Arthropathy
1.3
## Rutten et al., 2014; Pregabalin; Arthropathy
1.2
## Rutten et al., 2014; Pregabalin; Arthropathy
1.3
```

```
## Rutten et al., 2018; Gabapentin; Neuropathy: Trauma Injury
1.9
## Rutten et al., 2018; Gabapentin; Neuropathy: Trauma Injury
1.7
## Rutten et al., 2018; Gabapentin; Neuropathy: Diabetic-induced
2.1
## Rutten et al., 2018; Gabapentin; Neuropathy: Diabetic-induced
2.1
## Rutten et al., 2018; Morphine; Neuropathy: Trauma Injury
1.6
## Rutten et al., 2018; Morphine; Neuropathy: Trauma Injury
1.8
## Rutten et al., 2018; Morphine; Neuropathy: Trauma Injury
1.8
## Rutten et al., 2018; Morphine; Neuropathy: Diabetic-induced
1.6
## Rutten et al., 2018; Morphine; Neuropathy: Diabetic-induced
1.6
## Rutten et al., 2018; Morphine; Neuropathy: Diabetic-induced
1.6
## Rutten et al., 2018; Pregabalin; Neuropathy: Trauma Injury
1.5
## Rutten et al., 2018; Pregabalin; Neuropathy: Trauma Injury
1.4
## Rutten et al., 2018; Pregabalin; Neuropathy: Trauma Injury
1.7
## Rutten et al., 2018; Pregabalin; Neuropathy: Diabetic-induced
2.1
## Rutten et al., 2018; Pregabalin; Neuropathy: Diabetic-induced
2.1
## Rutten et al., 2018; Pregabalin; Neuropathy: Diabetic-induced
2.1
## Rutten et al., 2018; Pregabalin; Neuropathy: Diabetic-induced
2.1
## Rutten et al., 2018; Tramadol; Neuropathy: Diabetic-induced
0.7
## Rutten et al., 2018; Tramadol; Neuropathy: Diabetic-induced
1.6
## Rutten et al., 2018; Tramadol; Neuropathy: Diabetic-induced
0.7
##
## Number of studies combined: k = 67
##
##                          SMD            95%-CI    t  p-value
## Random effects model 0.5310 [ 0.2942; 0.7679] 4.48 < 0.0001
## Prediction interval         [-0.6325; 1.6946]
```

```
##
## Quantifying heterogeneity:
##  tau^2 = 0.3254 [0.2529; 1.1659]; tau = 0.5704 [0.5029; 1.0798]
##  I^2 = 50.2% [34.0%; 62.5%]; H = 1.42 [1.23; 1.63]
##
## Test of heterogeneity:
##       Q d.f.  p-value
##  132.59   66 < 0.0001
##
## Details on meta-analytical method:
## - Inverse variance method
## - Restricted maximum-likelihood estimator for tau^2
## - Q-profile method for confidence interval of tau^2 and tau
## - Hartung-Knapp adjustment for random effects model
```

```
# meta-analysis with removal of studies with high risk of bias
```

```
##                                                                 SMD
## Andrews et al., 2012; Ibuprofen; Somatic inflammation    1.5341
## Bryden et al., 2015; Celecoxib; Arthropathy              0.3582
## Bryden et al., 2015; Celecoxib; Arthropathy              0.7936
## Bryden et al., 2015; Celecoxib; Arthropathy              0.6324
## Bryden et al., 2015; Gabapentin ; Arthropathy           -0.2898
## Bryden et al., 2015; Gabapentin; Arthropathy            -0.0327
## Bryden et al., 2015; Gabapentin; Arthropathy            -0.0367
## Bryden et al., 2015; Ibuprofen; Arthropathy              0.5166
## Bryden et al., 2015; Ibuprofen; Arthropathy              0.4280
## Bryden et al., 2015; Ibuprofen; Arthropathy              0.2447
## Bryden et al., 2015; Morphine; Arthropathy               0.6919
## Bryden et al., 2015; Morphine; Arthropathy               0.1699
## Bryden et al., 2015; Morphine; Arthropathy               0.7987
## Bryden et al., 2015; Morphine; Arthropathy               0.7781
## Georgieva et al., 2019; Pregabalin; Spinal cord injury   2.3549
## Georgieva et al., 2019; Pregabalin; Spinal cord injury   1.7483
## Gould et al., 2016; Celecoxib; Somatic inflammation      0.9191
## Gould et al., 2016; Celecoxib; Somatic inflammation      0.3905
## Gould et al., 2016; Celecoxib; Somatic inflammation     -0.1064
## Gould et al., 2016; Gabapentin; Somatic inflammation    -0.0927
## Gould et al., 2016; Gabapentin; Somatic inflammation    -1.2407
## Gould et al., 2016; Gabapentin; Somatic inflammation    -0.1670
## Gould et al., 2016; Ibuprofen; Somatic inflammation      1.8166
## Gould et al., 2016; Indomethacin; Somatic inflammation   0.4873
## Gould et al., 2016; Indomethacin; Somatic inflammation   0.3141
## Gould et al., 2016; Indomethacin; Somatic inflammation   0.3659
## Gould et al., 2016; Morphine; Somatic inflammation       0.4095
## Gould et al., 2016; Morphine; Somatic inflammation       0.8877
## Gould et al., 2016; Morphine; Somatic inflammation      -0.1140
## Gould et al., 2016; Tramadol; Somatic inflammation       0.6187
## Gould et al., 2016; Tramadol; Somatic inflammation      -0.5030
## Gould et al., 2016; Tramadol; Somatic inflammation      -1.0887
## Katri et al., 2019; Naproxen; Arthropathy               -0.3977
## Lau et al., 2013; Pregabalin; Neuropathy: Trauma Injury  1.8393
##                                                          
95%-CI
## Andrews et al., 2012; Ibuprofen; Somatic inflammation    [ 0.4147; 
2.6536]
## Bryden et al., 2015; Celecoxib; Arthropathy              [-1.0409; 
1.7572]
## Bryden et al., 2015; Celecoxib; Arthropathy              [-0.6557; 
2.2429]
## Bryden et al., 2015; Celecoxib; Arthropathy              [-0.7941; 
2.0589]
## Bryden et al., 2015; Gabapentin ; Arthropathy            [-1.6843; 
```

```
1.1047]
## Bryden et al., 2015; Gabapentin; Arthropathy            [-1.4187;
1.3534]
## Bryden et al., 2015; Gabapentin; Arthropathy            [-1.4227;
1.3494]
## Bryden et al., 2015; Ibuprofen; Arthropathy             [-0.8141;
1.8474]
## Bryden et al., 2015; Ibuprofen; Arthropathy             [-0.8953;
1.7512]
## Bryden et al., 2015; Ibuprofen; Arthropathy             [-1.0674;
1.5568]
## Bryden et al., 2015; Morphine; Arthropathy              [-0.9058;
2.2896]
## Bryden et al., 2015; Morphine; Arthropathy              [-1.3826;
1.7223]
## Bryden et al., 2015; Morphine; Arthropathy              [-0.8147;
2.4121]
## Bryden et al., 2015; Morphine; Arthropathy              [-0.8321;
2.3883]
## Georgieva et al., 2019; Pregabalin; Spinal cord injury  [ 1.0988;
3.6110]
## Georgieva et al., 2019; Pregabalin; Spinal cord injury  [ 0.6168;
2.8797]
## Gould et al., 2016; Celecoxib; Somatic inflammation     [-0.3453;
2.1834]
## Gould et al., 2016; Celecoxib; Somatic inflammation     [-0.8186;
1.5996]
## Gould et al., 2016; Celecoxib; Somatic inflammation     [-1.2767;
1.0640]
## Gould et al., 2016; Gabapentin; Somatic inflammation    [-1.4001;
1.2147]
## Gould et al., 2016; Gabapentin; Somatic inflammation    [-2.6518;
0.1703]
## Gould et al., 2016; Gabapentin; Somatic inflammation    [-1.4595;
1.1255]
## Gould et al., 2016; Ibuprofen; Somatic inflammation     [ 0.6745;
2.9588]
## Gould et al., 2016; Indomethacin; Somatic inflammation  [-0.5986;
1.5731]
## Gould et al., 2016; Indomethacin; Somatic inflammation  [-0.7090;
1.3371]
## Gould et al., 2016; Indomethacin; Somatic inflammation  [-0.6809;
1.4126]
## Gould et al., 2016; Morphine; Somatic inflammation      [-0.8008;
1.6198]
## Gould et al., 2016; Morphine; Somatic inflammation      [-0.3525;
```

```
2.1278]
## Gould et al., 2016; Morphine; Somatic inflammation      [-1.3116;
1.0837]
## Gould et al., 2016; Tramadol; Somatic inflammation      [-0.8061;
2.0435]
## Gould et al., 2016; Tramadol; Somatic inflammation      [-1.9147;
0.9087]
## Gould et al., 2016; Tramadol; Somatic inflammation      [-2.5918;
0.4143]
## Katri et al., 2019; Naproxen; Arthropathy               [-1.6503;
0.8548]
## Lau et al., 2013; Pregabalin; Neuropathy: Trauma Injury [ 0.8574;
2.8212]
##
%W(random)
## Andrews et al., 2012; Ibuprofen; Somatic inflammation
3.5
## Bryden et al., 2015; Celecoxib; Arthropathy
2.6
## Bryden et al., 2015; Celecoxib; Arthropathy
2.5
## Bryden et al., 2015; Celecoxib; Arthropathy
2.6
## Bryden et al., 2015; Gabapentin ; Arthropathy
2.7
## Bryden et al., 2015; Gabapentin; Arthropathy
2.7
## Bryden et al., 2015; Gabapentin; Arthropathy
2.7
## Bryden et al., 2015; Ibuprofen; Arthropathy
2.8
## Bryden et al., 2015; Ibuprofen; Arthropathy
2.9
## Bryden et al., 2015; Ibuprofen; Arthropathy
2.9
## Bryden et al., 2015; Morphine; Arthropathy
2.2
## Bryden et al., 2015; Morphine; Arthropathy
2.3
## Bryden et al., 2015; Morphine; Arthropathy
2.2
## Bryden et al., 2015; Morphine; Arthropathy
2.2
## Georgieva et al., 2019; Pregabalin; Spinal cord injury
3.0
## Georgieva et al., 2019; Pregabalin; Spinal cord injury
```

```
3.5
## Gould et al., 2016; Celecoxib; Somatic inflammation
3.0
## Gould et al., 2016; Celecoxib; Somatic inflammation
3.2
## Gould et al., 2016; Celecoxib; Somatic inflammation
3.3
## Gould et al., 2016; Gabapentin; Somatic inflammation
2.9
## Gould et al., 2016; Gabapentin; Somatic inflammation
2.6
## Gould et al., 2016; Gabapentin; Somatic inflammation
2.9
## Gould et al., 2016; Ibuprofen; Somatic inflammation
3.4
## Gould et al., 2016; Indomethacin; Somatic inflammation
3.6
## Gould et al., 2016; Indomethacin; Somatic inflammation
3.9
## Gould et al., 2016; Indomethacin; Somatic inflammation
3.8
## Gould et al., 2016; Morphine; Somatic inflammation
3.2
## Gould et al., 2016; Morphine; Somatic inflammation
3.1
## Gould et al., 2016; Morphine; Somatic inflammation
3.2
## Gould et al., 2016; Tramadol; Somatic inflammation
2.6
## Gould et al., 2016; Tramadol; Somatic inflammation
2.6
## Gould et al., 2016; Tramadol; Somatic inflammation
2.4
## Katri et al., 2019; Naproxen; Arthropathy
3.1
## Lau et al., 2013; Pregabalin; Neuropathy: Trauma Injury
4.0
##
## Number of studies combined: k = 34
##
##                          SMD            95%-CI    t p-value
## Random effects model 0.4870 [ 0.2075; 0.7665] 3.55  0.0012
## Prediction interval         [-0.5566; 1.5307]
##
## Quantifying heterogeneity:
##  tau^2 = 0.2437 [0.0003; 0.6583]; tau = 0.4936 [0.0174; 0.8113]
```

```
##  I^2 = 35.0% [1.1%; 57.3%]; H = 1.24 [1.01; 1.53]
##
## Test of heterogeneity:
##      Q d.f. p-value
##  50.77   33  0.0248
##
## Details on meta-analytical method:
## - Inverse variance method
## - Restricted maximum-likelihood estimator for tau^2
## - Q-profile method for confidence interval of tau^2 and tau
## - Hartung-Knapp adjustment for random effects model
```

```
# meta-analysis based on "excluding studies that reported burrowing as a primary outcome measure from those reporting it as a secondary outcome measure"
```

The sensitivity test based on "excluding studies that reported burrowing as a primary outcome measure from those reporting it as a secondary outcome measure" was not possible as only five studies declared such information.

```
# influence analysis
```

```
## Leave-One-Out Analysis (Sorted by I2)
##  ------------------------------------
##
Effect
## Omitting Rutten et al., 2018; Tramadol; Neuropathy: Diabetic-
induced.2    0.616
## Omitting Rutten et al., 2018; Tramadol; Neuropathy: Diabetic-
induced      0.616
## Omitting Georgieva et al., 2019; Pregabalin; Spinal cord injury
0.555
## Omitting Gould et al., 2016; Gabapentin; Somatic inflammation.1
0.605
## Omitting Lau et al., 2013; Pregabalin; Neuropathy: Trauma Injury
0.558
## Omitting Gould et al., 2016; Tramadol; Somatic inflammation.2
0.601
## Omitting Rutten et al., 2014; Ibuprofen; Arthropathy.1
0.561
## Omitting Rutten et al., 2018; Pregabalin; Neuropathy: Trauma
Injury.1     0.560
## Omitting Gould et al., 2016; Ibuprofen; Somatic inflammation
0.561
## Omitting Georgieva et al., 2019; Pregabalin; Spinal cord injury.1
0.562
## Omitting Rutten et al., 2014; Ibuprofen; Arthropathy
0.562
## Omitting Rutten et al., 2014; Naproxen; Arthropathy
0.563
## Omitting Rutten et al., 2018; Gabapentin; Neuropathy: Trauma
Injury.1     0.563
## Omitting Rutten et al., 2018; Gabapentin; Neuropathy: Diabetic-
induced    0.599
## Omitting Rutten et al., 2018; Pregabalin; Neuropathy: Diabetic-
induced.3  0.598
## Omitting Rutten et al., 2018; Tramadol; Neuropathy: Diabetic-
induced.1    0.598
## Omitting Rutten et al., 2018; Pregabalin; Neuropathy: Trauma
Injury       0.564
## Omitting Andrews et al., 2012; Ibuprofen; Somatic inflammation
0.565
## Omitting Katri et al., 2019; Naproxen; Arthropathy
0.595
## Omitting Rutten et al., 2018; Pregabalin; Neuropathy: Diabetic-
induced.2  0.595
## Omitting Gould et al., 2016; Tramadol; Somatic inflammation.1
0.595
```

```
## Omitting Rutten et al., 2018; Morphine; Neuropathy: Trauma Injury
0.567
## Omitting Rutten et al., 2014; Pregabalin; Arthropathy
0.568
## Omitting Rutten et al., 2018; Pregabalin; Neuropathy: Diabetic-
induced.1  0.592
## Omitting Bryden et al., 2015; Gabapentin ; Arthropathy
0.592
## Omitting Rutten et al., 2014; Morphine; Arthropathy
0.570
## Omitting Rutten et al., 2014; Naproxen; Arthropathy.4
0.569
## Omitting Rutten et al., 2018; Morphine; Neuropathy: Diabetic-
induced.1    0.593
## Omitting Gould et al., 2016; Celecoxib; Somatic inflammation.2
0.592
## Omitting Gould et al., 2016; Gabapentin; Somatic inflammation.2
0.592
## Omitting Gould et al., 2016; Morphine; Somatic inflammation.2
0.592
## Omitting Rutten et al., 2014; Pregabalin; Arthropathy.3
0.570
## Omitting Rutten et al., 2018; Pregabalin; Neuropathy: Trauma
Injury.2     0.571
## Omitting Bryden et al., 2015; Gabapentin; Arthropathy
0.589
## Omitting Bryden et al., 2015; Gabapentin; Arthropathy.1
0.589
## Omitting Gould et al., 2016; Gabapentin; Somatic inflammation
0.590
## Omitting Rutten et al., 2014; Morphine; Arthropathy.1
0.572
## Omitting Rutten et al., 2014; Pregabalin; Arthropathy.4
0.571
## Omitting Rutten et al., 2018; Gabapentin; Neuropathy: Diabetic-
induced.1  0.588
## Omitting Rutten et al., 2014; Morphine; Arthropathy.2
0.574
## Omitting Rutten et al., 2014; Naproxen; Arthropathy.3
0.575
## Omitting Rutten et al., 2014; Pregabalin; Arthropathy.2
0.574
## Omitting Rutten et al., 2018; Gabapentin; Neuropathy: Trauma
Injury       0.573
## Omitting Rutten et al., 2018; Morphine; Neuropathy: Diabetic-
induced.2    0.588
```

```
## Omitting Rutten et al., 2018; Pregabalin; Neuropathy: Diabetic-
induced    0.587
## Omitting Bryden et al., 2015; Celecoxib; Arthropathy
0.583
## Omitting Bryden et al., 2015; Celecoxib; Arthropathy.1
0.578
## Omitting Bryden et al., 2015; Ibuprofen; Arthropathy.2
0.585
## Omitting Bryden et al., 2015; Morphine; Arthropathy.1
0.585
## Omitting Bryden et al., 2015; Morphine; Arthropathy.2
0.578
## Omitting Gould et al., 2016; Celecoxib; Somatic inflammation
0.576
## Omitting Gould et al., 2016; Celecoxib; Somatic inflammation.1
0.583
## Omitting Gould et al., 2016; Indomethacin; Somatic inflammation.1
0.585
## Omitting Gould et al., 2016; Indomethacin; Somatic inflammation.2
0.584
## Omitting Gould et al., 2016; Morphine; Somatic inflammation
0.583
## Omitting Gould et al., 2016; Morphine; Somatic inflammation.1
0.576
## Omitting Rutten et al., 2014; Naproxen; Arthropathy.1
0.575
## Omitting Rutten et al., 2018; Morphine; Neuropathy: Trauma
Injury.1       0.576
## Omitting Rutten et al., 2018; Morphine; Neuropathy: Trauma
Injury.2       0.577
## Omitting Rutten et al., 2018; Morphine; Neuropathy: Diabetic-
induced      0.584
## Omitting Bryden et al., 2015; Celecoxib; Arthropathy.2
0.580
## Omitting Bryden et al., 2015; Ibuprofen; Arthropathy
0.581
## Omitting Bryden et al., 2015; Ibuprofen; Arthropathy.1
0.583
## Omitting Bryden et al., 2015; Morphine; Arthropathy
0.579
## Omitting Bryden et al., 2015; Morphine; Arthropathy.3
0.578
## Omitting Gould et al., 2016; Indomethacin; Somatic inflammation
0.582
## Omitting Gould et al., 2016; Tramadol; Somatic inflammation
0.580
```

```
## Omitting Rutten et al., 2014; Naproxen; Arthropathy.2
0.582
## Omitting Rutten et al., 2014; Pregabalin; Arthropathy.1
0.582
##
LLCI
## Omitting Rutten et al., 2018; Tramadol; Neuropathy: Diabetic-
induced.2   0.397
## Omitting Rutten et al., 2018; Tramadol; Neuropathy: Diabetic-
induced     0.397
## Omitting Georgieva et al., 2019; Pregabalin; Spinal cord injury
0.317
## Omitting Gould et al., 2016; Gabapentin; Somatic inflammation.1
0.367
## Omitting Lau et al., 2013; Pregabalin; Neuropathy: Trauma Injury
0.316
## Omitting Gould et al., 2016; Tramadol; Somatic inflammation.2
0.361
## Omitting Rutten et al., 2014; Ibuprofen; Arthropathy.1
0.321
## Omitting Rutten et al., 2018; Pregabalin; Neuropathy: Trauma
Injury.1    0.320
## Omitting Gould et al., 2016; Ibuprofen; Somatic inflammation
0.319
## Omitting Georgieva et al., 2019; Pregabalin; Spinal cord injury.1
0.319
## Omitting Rutten et al., 2014; Ibuprofen; Arthropathy
0.321
## Omitting Rutten et al., 2014; Naproxen; Arthropathy
0.321
## Omitting Rutten et al., 2018; Gabapentin; Neuropathy: Trauma
Injury.1    0.320
## Omitting Rutten et al., 2018; Gabapentin; Neuropathy: Diabetic-
induced   0.355
## Omitting Rutten et al., 2018; Pregabalin; Neuropathy: Diabetic-
induced.3 0.354
## Omitting Rutten et al., 2018; Tramadol; Neuropathy: Diabetic-
induced.1   0.355
## Omitting Rutten et al., 2018; Pregabalin; Neuropathy: Trauma
Injury      0.321
## Omitting Andrews et al., 2012; Ibuprofen; Somatic inflammation
0.321
## Omitting Katri et al., 2019; Naproxen; Arthropathy
0.352
## Omitting Rutten et al., 2018; Pregabalin; Neuropathy: Diabetic-
induced.2 0.350
```

```
## Omitting Gould et al., 2016; Tramadol; Somatic inflammation.1
0.352
## Omitting Rutten et al., 2018; Morphine; Neuropathy: Trauma Injury
0.323
## Omitting Rutten et al., 2014; Pregabalin; Arthropathy
0.324
## Omitting Rutten et al., 2018; Pregabalin; Neuropathy: Diabetic-
induced.1 0.347
## Omitting Bryden et al., 2015; Gabapentin ; Arthropathy
0.348
## Omitting Rutten et al., 2014; Morphine; Arthropathy
0.326
## Omitting Rutten et al., 2014; Naproxen; Arthropathy.4
0.326
## Omitting Rutten et al., 2018; Morphine; Neuropathy: Diabetic-
induced.1   0.348
## Omitting Gould et al., 2016; Celecoxib; Somatic inflammation.2
0.347
## Omitting Gould et al., 2016; Gabapentin; Somatic inflammation.2
0.347
## Omitting Gould et al., 2016; Morphine; Somatic inflammation.2
0.347
## Omitting Rutten et al., 2014; Pregabalin; Arthropathy.3
0.326
## Omitting Rutten et al., 2018; Pregabalin; Neuropathy: Trauma
Injury.2    0.326
## Omitting Bryden et al., 2015; Gabapentin; Arthropathy
0.344
## Omitting Bryden et al., 2015; Gabapentin; Arthropathy.1
0.344
## Omitting Gould et al., 2016; Gabapentin; Somatic inflammation
0.346
## Omitting Rutten et al., 2014; Morphine; Arthropathy.1
0.327
## Omitting Rutten et al., 2014; Pregabalin; Arthropathy.4
0.327
## Omitting Rutten et al., 2018; Gabapentin; Neuropathy: Diabetic-
induced.1 0.342
## Omitting Rutten et al., 2014; Morphine; Arthropathy.2
0.328
## Omitting Rutten et al., 2014; Naproxen; Arthropathy.3
0.329
## Omitting Rutten et al., 2014; Pregabalin; Arthropathy.2
0.329
## Omitting Rutten et al., 2018; Gabapentin; Neuropathy: Trauma
Injury      0.327
```

```
## Omitting Rutten et al., 2018; Morphine; Neuropathy: Diabetic-induced.2   0.343
## Omitting Rutten et al., 2018; Pregabalin; Neuropathy: Diabetic-induced   0.341
## Omitting Bryden et al., 2015; Celecoxib; Arthropathy
0.338
## Omitting Bryden et al., 2015; Celecoxib; Arthropathy.1
0.332
## Omitting Bryden et al., 2015; Ibuprofen; Arthropathy.2
0.340
## Omitting Bryden et al., 2015; Morphine; Arthropathy.1
0.340
## Omitting Bryden et al., 2015; Morphine; Arthropathy.2
0.333
## Omitting Gould et al., 2016; Celecoxib; Somatic inflammation
0.330
## Omitting Gould et al., 2016; Celecoxib; Somatic inflammation.1
0.337
## Omitting Gould et al., 2016; Indomethacin; Somatic inflammation.1
0.339
## Omitting Gould et al., 2016; Indomethacin; Somatic inflammation.2
0.338
## Omitting Gould et al., 2016; Morphine; Somatic inflammation
0.337
## Omitting Gould et al., 2016; Morphine; Somatic inflammation.1
0.330
## Omitting Rutten et al., 2014; Naproxen; Arthropathy.1
0.329
## Omitting Rutten et al., 2018; Morphine; Neuropathy: Trauma Injury.1       0.330
## Omitting Rutten et al., 2018; Morphine; Neuropathy: Trauma Injury.2       0.330
## Omitting Rutten et al., 2018; Morphine; Neuropathy: Diabetic-induced     0.338
## Omitting Bryden et al., 2015; Celecoxib; Arthropathy.2
0.334
## Omitting Bryden et al., 2015; Ibuprofen; Arthropathy
0.336
## Omitting Bryden et al., 2015; Ibuprofen; Arthropathy.1
0.337
## Omitting Bryden et al., 2015; Morphine; Arthropathy
0.334
## Omitting Bryden et al., 2015; Morphine; Arthropathy.3
0.333
## Omitting Gould et al., 2016; Indomethacin; Somatic inflammation
0.336
```

```
## Omitting Gould et al., 2016; Tramadol; Somatic inflammation
0.334
## Omitting Rutten et al., 2014; Naproxen; Arthropathy.2
0.336
## Omitting Rutten et al., 2014; Pregabalin; Arthropathy.1
0.336
##
ULCI
## Omitting Rutten et al., 2018; Tramadol; Neuropathy: Diabetic-
induced.2   0.834
## Omitting Rutten et al., 2018; Tramadol; Neuropathy: Diabetic-
induced     0.835
## Omitting Georgieva et al., 2019; Pregabalin; Spinal cord injury
0.793
## Omitting Gould et al., 2016; Gabapentin; Somatic inflammation.1
0.844
## Omitting Lau et al., 2013; Pregabalin; Neuropathy: Trauma Injury
0.799
## Omitting Gould et al., 2016; Tramadol; Somatic inflammation.2
0.842
## Omitting Rutten et al., 2014; Ibuprofen; Arthropathy.1
0.801
## Omitting Rutten et al., 2018; Pregabalin; Neuropathy: Trauma
Injury.1    0.801
## Omitting Gould et al., 2016; Ibuprofen; Somatic inflammation
0.803
## Omitting Georgieva et al., 2019; Pregabalin; Spinal cord injury.1
0.804
## Omitting Rutten et al., 2014; Ibuprofen; Arthropathy
0.803
## Omitting Rutten et al., 2014; Naproxen; Arthropathy
0.804
## Omitting Rutten et al., 2018; Gabapentin; Neuropathy: Trauma
Injury.1    0.805
## Omitting Rutten et al., 2018; Gabapentin; Neuropathy: Diabetic-
induced   0.842
## Omitting Rutten et al., 2018; Pregabalin; Neuropathy: Diabetic-
induced.3 0.842
## Omitting Rutten et al., 2018; Tramadol; Neuropathy: Diabetic-
induced.1   0.841
## Omitting Rutten et al., 2018; Pregabalin; Neuropathy: Trauma
Injury      0.807
## Omitting Andrews et al., 2012; Ibuprofen; Somatic inflammation
0.809
## Omitting Katri et al., 2019; Naproxen; Arthropathy
0.839
```

```
## Omitting Rutten et al., 2018; Pregabalin; Neuropathy: Diabetic-
induced.2 0.840
## Omitting Gould et al., 2016; Tramadol; Somatic inflammation.1
0.838
## Omitting Rutten et al., 2018; Morphine; Neuropathy: Trauma Injury
0.810
## Omitting Rutten et al., 2014; Pregabalin; Arthropathy
0.813
## Omitting Rutten et al., 2018; Pregabalin; Neuropathy: Diabetic-
induced.1 0.838
## Omitting Bryden et al., 2015; Gabapentin ; Arthropathy
0.836
## Omitting Rutten et al., 2014; Morphine; Arthropathy
0.814
## Omitting Rutten et al., 2014; Naproxen; Arthropathy.4
0.813
## Omitting Rutten et al., 2018; Morphine; Neuropathy: Diabetic-
induced.1   0.837
## Omitting Gould et al., 2016; Celecoxib; Somatic inflammation.2
0.836
## Omitting Gould et al., 2016; Gabapentin; Somatic inflammation.2
0.836
## Omitting Gould et al., 2016; Morphine; Somatic inflammation.2
0.836
## Omitting Rutten et al., 2014; Pregabalin; Arthropathy.3
0.814
## Omitting Rutten et al., 2018; Pregabalin; Neuropathy: Trauma
Injury.2    0.816
## Omitting Bryden et al., 2015; Gabapentin; Arthropathy
0.834
## Omitting Bryden et al., 2015; Gabapentin; Arthropathy.1
0.834
## Omitting Gould et al., 2016; Gabapentin; Somatic inflammation
0.835
## Omitting Rutten et al., 2014; Morphine; Arthropathy.1
0.817
## Omitting Rutten et al., 2014; Pregabalin; Arthropathy.4
0.816
## Omitting Rutten et al., 2018; Gabapentin; Neuropathy: Diabetic-
induced.1 0.834
## Omitting Rutten et al., 2014; Morphine; Arthropathy.2
0.819
## Omitting Rutten et al., 2014; Naproxen; Arthropathy.3
0.820
## Omitting Rutten et al., 2014; Pregabalin; Arthropathy.2
0.820
```

```
## Omitting Rutten et al., 2018; Gabapentin; Neuropathy: Trauma
Injury      0.820
## Omitting Rutten et al., 2018; Morphine; Neuropathy: Diabetic-
induced.2   0.834
## Omitting Rutten et al., 2018; Pregabalin; Neuropathy: Diabetic-
induced   0.834
## Omitting Bryden et al., 2015; Celecoxib; Arthropathy
0.829
## Omitting Bryden et al., 2015; Celecoxib; Arthropathy.1
0.823
## Omitting Bryden et al., 2015; Ibuprofen; Arthropathy.2
0.831
## Omitting Bryden et al., 2015; Morphine; Arthropathy.1
0.831
## Omitting Bryden et al., 2015; Morphine; Arthropathy.2
0.823
## Omitting Gould et al., 2016; Celecoxib; Somatic inflammation
0.821
## Omitting Gould et al., 2016; Celecoxib; Somatic inflammation.1
0.829
## Omitting Gould et al., 2016; Indomethacin; Somatic inflammation.1
0.831
## Omitting Gould et al., 2016; Indomethacin; Somatic inflammation.2
0.830
## Omitting Gould et al., 2016; Morphine; Somatic inflammation
0.829
## Omitting Gould et al., 2016; Morphine; Somatic inflammation.1
0.822
## Omitting Rutten et al., 2014; Naproxen; Arthropathy.1
0.821
## Omitting Rutten et al., 2018; Morphine; Neuropathy: Trauma
Injury.1      0.822
## Omitting Rutten et al., 2018; Morphine; Neuropathy: Trauma
Injury.2      0.823
## Omitting Rutten et al., 2018; Morphine; Neuropathy: Diabetic-
induced     0.830
## Omitting Bryden et al., 2015; Celecoxib; Arthropathy.2
0.825
## Omitting Bryden et al., 2015; Ibuprofen; Arthropathy
0.827
## Omitting Bryden et al., 2015; Ibuprofen; Arthropathy.1
0.828
## Omitting Bryden et al., 2015; Morphine; Arthropathy
0.825
## Omitting Bryden et al., 2015; Morphine; Arthropathy.3
0.824
```

```
## Omitting Gould et al., 2016; Indomethacin; Somatic inflammation
0.828
## Omitting Gould et al., 2016; Tramadol; Somatic inflammation
0.826
## Omitting Rutten et al., 2014; Naproxen; Arthropathy.2
0.828
## Omitting Rutten et al., 2014; Pregabalin; Arthropathy.1
0.828
##
I2
## Omitting Rutten et al., 2018; Tramadol; Neuropathy: Diabetic-
induced.2   0.473
## Omitting Rutten et al., 2018; Tramadol; Neuropathy: Diabetic-
induced     0.474
## Omitting Georgieva et al., 2019; Pregabalin; Spinal cord injury
0.519
## Omitting Gould et al., 2016; Gabapentin; Somatic inflammation.1
0.524
## Omitting Lau et al., 2013; Pregabalin; Neuropathy: Trauma Injury
0.524
## Omitting Gould et al., 2016; Tramadol; Somatic inflammation.2
0.529
## Omitting Rutten et al., 2014; Ibuprofen; Arthropathy.1
0.529
## Omitting Rutten et al., 2018; Pregabalin; Neuropathy: Trauma
Injury.1    0.529
## Omitting Gould et al., 2016; Ibuprofen; Somatic inflammation
0.530
## Omitting Georgieva et al., 2019; Pregabalin; Spinal cord injury.1
0.531
## Omitting Rutten et al., 2014; Ibuprofen; Arthropathy
0.531
## Omitting Rutten et al., 2014; Naproxen; Arthropathy
0.532
## Omitting Rutten et al., 2018; Gabapentin; Neuropathy: Trauma
Injury.1    0.532
## Omitting Rutten et al., 2018; Gabapentin; Neuropathy: Diabetic-
induced   0.533
## Omitting Rutten et al., 2018; Pregabalin; Neuropathy: Diabetic-
induced.3 0.533
## Omitting Rutten et al., 2018; Tramadol; Neuropathy: Diabetic-
induced.1   0.534
## Omitting Rutten et al., 2018; Pregabalin; Neuropathy: Trauma
Injury      0.535
## Omitting Andrews et al., 2012; Ibuprofen; Somatic inflammation
0.536
```

```
## Omitting Katri et al., 2019; Naproxen; Arthropathy
0.537
## Omitting Rutten et al., 2018; Pregabalin; Neuropathy: Diabetic-
induced.2 0.537
## Omitting Gould et al., 2016; Tramadol; Somatic inflammation.1
0.538
## Omitting Rutten et al., 2018; Morphine; Neuropathy: Trauma Injury
0.538
## Omitting Rutten et al., 2014; Pregabalin; Arthropathy
0.539
## Omitting Rutten et al., 2018; Pregabalin; Neuropathy: Diabetic-
induced.1 0.539
## Omitting Bryden et al., 2015; Gabapentin ; Arthropathy
0.540
## Omitting Rutten et al., 2014; Morphine; Arthropathy
0.540
## Omitting Rutten et al., 2014; Naproxen; Arthropathy.4
0.540
## Omitting Rutten et al., 2018; Morphine; Neuropathy: Diabetic-
induced.1   0.540
## Omitting Gould et al., 2016; Celecoxib; Somatic inflammation.2
0.541
## Omitting Gould et al., 2016; Gabapentin; Somatic inflammation.2
0.541
## Omitting Gould et al., 2016; Morphine; Somatic inflammation.2
0.541
## Omitting Rutten et al., 2014; Pregabalin; Arthropathy.3
0.541
## Omitting Rutten et al., 2018; Pregabalin; Neuropathy: Trauma
Injury.2    0.541
## Omitting Bryden et al., 2015; Gabapentin; Arthropathy
0.542
## Omitting Bryden et al., 2015; Gabapentin; Arthropathy.1
0.542
## Omitting Gould et al., 2016; Gabapentin; Somatic inflammation
0.542
## Omitting Rutten et al., 2014; Morphine; Arthropathy.1
0.542
## Omitting Rutten et al., 2014; Pregabalin; Arthropathy.4
0.542
## Omitting Rutten et al., 2018; Gabapentin; Neuropathy: Diabetic-
induced.1 0.542
## Omitting Rutten et al., 2014; Morphine; Arthropathy.2
0.543
## Omitting Rutten et al., 2014; Naproxen; Arthropathy.3
0.543
```

```
## Omitting Rutten et al., 2014; Pregabalin; Arthropathy.2
0.543
## Omitting Rutten et al., 2018; Gabapentin; Neuropathy: Trauma
Injury      0.543
## Omitting Rutten et al., 2018; Morphine; Neuropathy: Diabetic-
induced.2   0.543
## Omitting Rutten et al., 2018; Pregabalin; Neuropathy: Diabetic-
induced   0.543
## Omitting Bryden et al., 2015; Celecoxib; Arthropathy
0.544
## Omitting Bryden et al., 2015; Celecoxib; Arthropathy.1
0.544
## Omitting Bryden et al., 2015; Ibuprofen; Arthropathy.2
0.544
## Omitting Bryden et al., 2015; Morphine; Arthropathy.1
0.544
## Omitting Bryden et al., 2015; Morphine; Arthropathy.2
0.544
## Omitting Gould et al., 2016; Celecoxib; Somatic inflammation
0.544
## Omitting Gould et al., 2016; Celecoxib; Somatic inflammation.1
0.544
## Omitting Gould et al., 2016; Indomethacin; Somatic inflammation.1
0.544
## Omitting Gould et al., 2016; Indomethacin; Somatic inflammation.2
0.544
## Omitting Gould et al., 2016; Morphine; Somatic inflammation
0.544
## Omitting Gould et al., 2016; Morphine; Somatic inflammation.1
0.544
## Omitting Rutten et al., 2014; Naproxen; Arthropathy.1
0.544
## Omitting Rutten et al., 2018; Morphine; Neuropathy: Trauma
Injury.1      0.544
## Omitting Rutten et al., 2018; Morphine; Neuropathy: Trauma
Injury.2      0.544
## Omitting Rutten et al., 2018; Morphine; Neuropathy: Diabetic-
induced     0.544
## Omitting Bryden et al., 2015; Celecoxib; Arthropathy.2
0.545
## Omitting Bryden et al., 2015; Ibuprofen; Arthropathy
0.545
## Omitting Bryden et al., 2015; Ibuprofen; Arthropathy.1
0.545
## Omitting Bryden et al., 2015; Morphine; Arthropathy
0.545
```

```
## Omitting Bryden et al., 2015; Morphine; Arthropathy.3
0.545
## Omitting Gould et al., 2016; Indomethacin; Somatic inflammation
0.545
## Omitting Gould et al., 2016; Tramadol; Somatic inflammation
0.545
## Omitting Rutten et al., 2014; Naproxen; Arthropathy.2
0.545
## Omitting Rutten et al., 2014; Pregabalin; Arthropathy.1
0.545
##
##
## Influence Diagnostics
##  -------------------
##
rstudent
## Omitting Andrews et al., 2012; Ibuprofen; Somatic inflammation
1.127
## Omitting Bryden et al., 2015; Celecoxib; Arthropathy
-0.232
## Omitting Bryden et al., 2015; Celecoxib; Arthropathy.1
0.218
## Omitting Bryden et al., 2015; Celecoxib; Arthropathy.2
0.054
## Omitting Bryden et al., 2015; Gabapentin ; Arthropathy
-0.917
## Omitting Bryden et al., 2015; Gabapentin; Arthropathy
-0.646
## Omitting Bryden et al., 2015; Gabapentin; Arthropathy.1
-0.650
## Omitting Bryden et al., 2015; Ibuprofen; Arthropathy
-0.069
## Omitting Bryden et al., 2015; Ibuprofen; Arthropathy.1
-0.164
## Omitting Bryden et al., 2015; Ibuprofen; Arthropathy.2
-0.364
## Omitting Bryden et al., 2015; Morphine; Arthropathy
0.108
## Omitting Bryden et al., 2015; Morphine; Arthropathy.1
-0.405
## Omitting Bryden et al., 2015; Morphine; Arthropathy.2
0.210
## Omitting Bryden et al., 2015; Morphine; Arthropathy.3
0.190
## Omitting Georgieva et al., 2019; Pregabalin; Spinal cord injury
2.035
```

```
## Omitting Georgieva et al., 2019; Pregabalin; Spinal cord injury.1
1.384
## Omitting Gould et al., 2016; Celecoxib; Somatic inflammation
0.373
## Omitting Gould et al., 2016; Celecoxib; Somatic inflammation.1
-0.214
## Omitting Gould et al., 2016; Celecoxib; Somatic inflammation.2
-0.791
## Omitting Gould et al., 2016; Gabapentin; Somatic inflammation
-0.733
## Omitting Gould et al., 2016; Gabapentin; Somatic inflammation.1
-1.949
## Omitting Gould et al., 2016; Gabapentin; Somatic inflammation.2
-0.820
## Omitting Gould et al., 2016; Ibuprofen; Somatic inflammation
1.462
## Omitting Gould et al., 2016; Indomethacin; Somatic inflammation
-0.110
## Omitting Gould et al., 2016; Indomethacin; Somatic inflammation.1
-0.323
## Omitting Gould et al., 2016; Indomethacin; Somatic inflammation.2
-0.257
## Omitting Gould et al., 2016; Morphine; Somatic inflammation
-0.192
## Omitting Gould et al., 2016; Morphine; Somatic inflammation.1
0.342
## Omitting Gould et al., 2016; Morphine; Somatic inflammation.2
-0.791
## Omitting Gould et al., 2016; Tramadol; Somatic inflammation
0.039
## Omitting Gould et al., 2016; Tramadol; Somatic inflammation.1
-1.138
## Omitting Gould et al., 2016; Tramadol; Somatic inflammation.2
-1.707
## Omitting Katri et al., 2019; Naproxen; Arthropathy
-1.096
## Omitting Lau et al., 2013; Pregabalin; Neuropathy: Trauma Injury
1.604
## Omitting Rutten et al., 2014; Ibuprofen; Arthropathy
1.608
## Omitting Rutten et al., 2014; Ibuprofen; Arthropathy.1
1.738
## Omitting Rutten et al., 2014; Morphine; Arthropathy
0.900
## Omitting Rutten et al., 2014; Morphine; Arthropathy.1
0.698
```

```
## Omitting Rutten et al., 2014; Morphine; Arthropathy.2
0.562
## Omitting Rutten et al., 2014; Naproxen; Arthropathy
1.511
## Omitting Rutten et al., 2014; Naproxen; Arthropathy.1
0.412
## Omitting Rutten et al., 2014; Naproxen; Arthropathy.2
-0.127
## Omitting Rutten et al., 2014; Naproxen; Arthropathy.3
0.468
## Omitting Rutten et al., 2014; Naproxen; Arthropathy.4
0.934
## Omitting Rutten et al., 2014; Pregabalin; Arthropathy
0.933
## Omitting Rutten et al., 2014; Pregabalin; Arthropathy.1
-0.119
## Omitting Rutten et al., 2014; Pregabalin; Arthropathy.2
0.483
## Omitting Rutten et al., 2014; Pregabalin; Arthropathy.3
0.880
## Omitting Rutten et al., 2014; Pregabalin; Arthropathy.4
0.747
## Omitting Rutten et al., 2018; Gabapentin; Neuropathy: Trauma
Injury          0.468
## Omitting Rutten et al., 2018; Gabapentin; Neuropathy: Trauma
Injury.1        1.312
## Omitting Rutten et al., 2018; Gabapentin; Neuropathy: Diabetic-
induced      -1.151
## Omitting Rutten et al., 2018; Gabapentin; Neuropathy: Diabetic-
induced.1    -0.492
## Omitting Rutten et al., 2018; Morphine; Neuropathy: Trauma Injury
1.048
## Omitting Rutten et al., 2018; Morphine; Neuropathy: Trauma
Injury.1          0.308
## Omitting Rutten et al., 2018; Morphine; Neuropathy: Trauma
Injury.2          0.271
## Omitting Rutten et al., 2018; Morphine; Neuropathy: Diabetic-
induced        -0.273
## Omitting Rutten et al., 2018; Morphine; Neuropathy: Diabetic-
induced.1      -0.883
## Omitting Rutten et al., 2018; Morphine; Neuropathy: Diabetic-
induced.2      -0.566
## Omitting Rutten et al., 2018; Pregabalin; Neuropathy: Trauma
Injury          1.268
## Omitting Rutten et al., 2018; Pregabalin; Neuropathy: Trauma
Injury.1        1.640
```

```
## Omitting Rutten et al., 2018; Pregabalin; Neuropathy: Trauma
Injury.2       0.676
## Omitting Rutten et al., 2018; Pregabalin; Neuropathy: Diabetic-
induced     -0.443
## Omitting Rutten et al., 2018; Pregabalin; Neuropathy: Diabetic-
induced.1   -0.755
## Omitting Rutten et al., 2018; Pregabalin; Neuropathy: Diabetic-
induced.2   -0.919
## Omitting Rutten et al., 2018; Pregabalin; Neuropathy: Diabetic-
induced.3   -1.132
## Omitting Rutten et al., 2018; Tramadol; Neuropathy: Diabetic-
induced       -3.982
## Omitting Rutten et al., 2018; Tramadol; Neuropathy: Diabetic-
induced.1     -1.260
## Omitting Rutten et al., 2018; Tramadol; Neuropathy: Diabetic-
induced.2     -4.038
##
dffits
## Omitting Andrews et al., 2012; Ibuprofen; Somatic inflammation
0.145
## Omitting Bryden et al., 2015; Celecoxib; Arthropathy
-0.025
## Omitting Bryden et al., 2015; Celecoxib; Arthropathy.1
0.027
## Omitting Bryden et al., 2015; Celecoxib; Arthropathy.2
0.009
## Omitting Bryden et al., 2015; Gabapentin ; Arthropathy
-0.106
## Omitting Bryden et al., 2015; Gabapentin; Arthropathy
-0.074
## Omitting Bryden et al., 2015; Gabapentin; Arthropathy.1
-0.074
## Omitting Bryden et al., 2015; Ibuprofen; Arthropathy
-0.006
## Omitting Bryden et al., 2015; Ibuprofen; Arthropathy.1
-0.017
## Omitting Bryden et al., 2015; Ibuprofen; Arthropathy.2
-0.041
## Omitting Bryden et al., 2015; Morphine; Arthropathy
0.014
## Omitting Bryden et al., 2015; Morphine; Arthropathy.1
-0.042
## Omitting Bryden et al., 2015; Morphine; Arthropathy.2
0.024
## Omitting Bryden et al., 2015; Morphine; Arthropathy.3
0.022
```

```
## Omitting Georgieva et al., 2019; Pregabalin; Spinal cord injury
0.240
## Omitting Georgieva et al., 2019; Pregabalin; Spinal cord injury.1
0.175
## Omitting Gould et al., 2016; Celecoxib; Somatic inflammation
0.048
## Omitting Gould et al., 2016; Celecoxib; Somatic inflammation.1
-0.024
## Omitting Gould et al., 2016; Celecoxib; Somatic inflammation.2
-0.099
## Omitting Gould et al., 2016; Gabapentin; Somatic inflammation
-0.087
## Omitting Gould et al., 2016; Gabapentin; Somatic inflammation.1
-0.228
## Omitting Gould et al., 2016; Gabapentin; Somatic inflammation.2
-0.098
## Omitting Gould et al., 2016; Ibuprofen; Somatic inflammation
0.184
## Omitting Gould et al., 2016; Indomethacin; Somatic inflammation
-0.011
## Omitting Gould et al., 2016; Indomethacin; Somatic inflammation.1
-0.040
## Omitting Gould et al., 2016; Indomethacin; Somatic inflammation.2
-0.031
## Omitting Gould et al., 2016; Morphine; Somatic inflammation
-0.021
## Omitting Gould et al., 2016; Morphine; Somatic inflammation.1
0.044
## Omitting Gould et al., 2016; Morphine; Somatic inflammation.2
-0.098
## Omitting Gould et al., 2016; Tramadol; Somatic inflammation
0.007
## Omitting Gould et al., 2016; Tramadol; Somatic inflammation.1
-0.131
## Omitting Gould et al., 2016; Tramadol; Somatic inflammation.2
-0.192
## Omitting Katri et al., 2019; Naproxen; Arthropathy
-0.135
## Omitting Lau et al., 2013; Pregabalin; Neuropathy: Trauma Injury
0.215
## Omitting Rutten et al., 2014; Ibuprofen; Arthropathy
0.175
## Omitting Rutten et al., 2014; Ibuprofen; Arthropathy.1
0.185
## Omitting Rutten et al., 2014; Morphine; Arthropathy
0.101
```

```
## Omitting Rutten et al., 2014; Morphine; Arthropathy.1
0.080
## Omitting Rutten et al., 2014; Morphine; Arthropathy.2
0.066
## Omitting Rutten et al., 2014; Naproxen; Arthropathy
0.167
## Omitting Rutten et al., 2014; Naproxen; Arthropathy.1
0.054
## Omitting Rutten et al., 2014; Naproxen; Arthropathy.2
-0.013
## Omitting Rutten et al., 2014; Naproxen; Arthropathy.3
0.056
## Omitting Rutten et al., 2014; Naproxen; Arthropathy.4
0.104
## Omitting Rutten et al., 2014; Pregabalin; Arthropathy
0.112
## Omitting Rutten et al., 2014; Pregabalin; Arthropathy.1
-0.012
## Omitting Rutten et al., 2014; Pregabalin; Arthropathy.2
0.058
## Omitting Rutten et al., 2014; Pregabalin; Arthropathy.3
0.099
## Omitting Rutten et al., 2014; Pregabalin; Arthropathy.4
0.085
## Omitting Rutten et al., 2018; Gabapentin; Neuropathy: Trauma
Injury        0.066
## Omitting Rutten et al., 2018; Gabapentin; Neuropathy: Trauma
Injury.1      0.168
## Omitting Rutten et al., 2018; Gabapentin; Neuropathy: Diabetic-
induced   -0.163
## Omitting Rutten et al., 2018; Gabapentin; Neuropathy: Diabetic-
induced.1 -0.066
## Omitting Rutten et al., 2018; Morphine; Neuropathy: Trauma Injury
0.130
## Omitting Rutten et al., 2018; Morphine; Neuropathy: Trauma
Injury.1        0.044
## Omitting Rutten et al., 2018; Morphine; Neuropathy: Trauma
Injury.2        0.038
## Omitting Rutten et al., 2018; Morphine; Neuropathy: Diabetic-
induced      -0.032
## Omitting Rutten et al., 2018; Morphine; Neuropathy: Diabetic-
induced.1    -0.110
## Omitting Rutten et al., 2018; Morphine; Neuropathy: Diabetic-
induced.2    -0.069
## Omitting Rutten et al., 2018; Pregabalin; Neuropathy: Trauma
Injury        0.153
```

```
## Omitting Rutten et al., 2018; Pregabalin; Neuropathy: Trauma
Injury.1      0.188
## Omitting Rutten et al., 2018; Pregabalin; Neuropathy: Trauma
Injury.2      0.088
## Omitting Rutten et al., 2018; Pregabalin; Neuropathy: Diabetic-
induced    -0.059
## Omitting Rutten et al., 2018; Pregabalin; Neuropathy: Diabetic-
induced.1 -0.104
## Omitting Rutten et al., 2018; Pregabalin; Neuropathy: Diabetic-
induced.2 -0.130
## Omitting Rutten et al., 2018; Pregabalin; Neuropathy: Diabetic-
induced.3 -0.160
## Omitting Rutten et al., 2018; Tramadol; Neuropathy: Diabetic-
induced      -0.331
## Omitting Rutten et al., 2018; Tramadol; Neuropathy: Diabetic-
induced.1    -0.159
## Omitting Rutten et al., 2018; Tramadol; Neuropathy: Diabetic-
induced.2    -0.326
##
cook.d
## Omitting Andrews et al., 2012; Ibuprofen; Somatic inflammation
0.021
## Omitting Bryden et al., 2015; Celecoxib; Arthropathy
0.001
## Omitting Bryden et al., 2015; Celecoxib; Arthropathy.1
0.001
## Omitting Bryden et al., 2015; Celecoxib; Arthropathy.2
0.000
## Omitting Bryden et al., 2015; Gabapentin ; Arthropathy
0.011
## Omitting Bryden et al., 2015; Gabapentin; Arthropathy
0.005
## Omitting Bryden et al., 2015; Gabapentin; Arthropathy.1
0.006
## Omitting Bryden et al., 2015; Ibuprofen; Arthropathy
0.000
## Omitting Bryden et al., 2015; Ibuprofen; Arthropathy.1
0.000
## Omitting Bryden et al., 2015; Ibuprofen; Arthropathy.2
0.002
## Omitting Bryden et al., 2015; Morphine; Arthropathy
0.000
## Omitting Bryden et al., 2015; Morphine; Arthropathy.1
0.002
## Omitting Bryden et al., 2015; Morphine; Arthropathy.2
0.001
```

```
## Omitting Bryden et al., 2015; Morphine; Arthropathy.3
0.000
## Omitting Georgieva et al., 2019; Pregabalin; Spinal cord injury
0.055
## Omitting Georgieva et al., 2019; Pregabalin; Spinal cord injury.1
0.030
## Omitting Gould et al., 2016; Celecoxib; Somatic inflammation
0.002
## Omitting Gould et al., 2016; Celecoxib; Somatic inflammation.1
0.001
## Omitting Gould et al., 2016; Celecoxib; Somatic inflammation.2
0.010
## Omitting Gould et al., 2016; Gabapentin; Somatic inflammation
0.008
## Omitting Gould et al., 2016; Gabapentin; Somatic inflammation.1
0.050
## Omitting Gould et al., 2016; Gabapentin; Somatic inflammation.2
0.010
## Omitting Gould et al., 2016; Ibuprofen; Somatic inflammation
0.033
## Omitting Gould et al., 2016; Indomethacin; Somatic inflammation
0.000
## Omitting Gould et al., 2016; Indomethacin; Somatic inflammation.1
0.002
## Omitting Gould et al., 2016; Indomethacin; Somatic inflammation.2
0.001
## Omitting Gould et al., 2016; Morphine; Somatic inflammation
0.000
## Omitting Gould et al., 2016; Morphine; Somatic inflammation.1
0.002
## Omitting Gould et al., 2016; Morphine; Somatic inflammation.2
0.010
## Omitting Gould et al., 2016; Tramadol; Somatic inflammation
0.000
## Omitting Gould et al., 2016; Tramadol; Somatic inflammation.1
0.017
## Omitting Gould et al., 2016; Tramadol; Somatic inflammation.2
0.036
## Omitting Katri et al., 2019; Naproxen; Arthropathy
0.018
## Omitting Lau et al., 2013; Pregabalin; Neuropathy: Trauma Injury
0.044
## Omitting Rutten et al., 2014; Ibuprofen; Arthropathy
0.030
## Omitting Rutten et al., 2014; Ibuprofen; Arthropathy.1
0.033
```

```
## Omitting Rutten et al., 2014; Morphine; Arthropathy
0.010
## Omitting Rutten et al., 2014; Morphine; Arthropathy.1
0.007
## Omitting Rutten et al., 2014; Morphine; Arthropathy.2
0.004
## Omitting Rutten et al., 2014; Naproxen; Arthropathy
0.027
## Omitting Rutten et al., 2014; Naproxen; Arthropathy.1
0.003
## Omitting Rutten et al., 2014; Naproxen; Arthropathy.2
0.000
## Omitting Rutten et al., 2014; Naproxen; Arthropathy.3
0.003
## Omitting Rutten et al., 2014; Naproxen; Arthropathy.4
0.011
## Omitting Rutten et al., 2014; Pregabalin; Arthropathy
0.013
## Omitting Rutten et al., 2014; Pregabalin; Arthropathy.1
0.000
## Omitting Rutten et al., 2014; Pregabalin; Arthropathy.2
0.003
## Omitting Rutten et al., 2014; Pregabalin; Arthropathy.3
0.010
## Omitting Rutten et al., 2014; Pregabalin; Arthropathy.4
0.007
## Omitting Rutten et al., 2018; Gabapentin; Neuropathy: Trauma
Injury       0.004
## Omitting Rutten et al., 2018; Gabapentin; Neuropathy: Trauma
Injury.1     0.028
## Omitting Rutten et al., 2018; Gabapentin; Neuropathy: Diabetic-
induced    0.026
## Omitting Rutten et al., 2018; Gabapentin; Neuropathy: Diabetic-
induced.1  0.004
## Omitting Rutten et al., 2018; Morphine; Neuropathy: Trauma Injury
0.017
## Omitting Rutten et al., 2018; Morphine; Neuropathy: Trauma
Injury.1       0.002
## Omitting Rutten et al., 2018; Morphine; Neuropathy: Trauma
Injury.2       0.001
## Omitting Rutten et al., 2018; Morphine; Neuropathy: Diabetic-
induced      0.001
## Omitting Rutten et al., 2018; Morphine; Neuropathy: Diabetic-
induced.1    0.012
## Omitting Rutten et al., 2018; Morphine; Neuropathy: Diabetic-
induced.2    0.005
```

```
## Omitting Rutten et al., 2018; Pregabalin; Neuropathy: Trauma
Injury        0.023
## Omitting Rutten et al., 2018; Pregabalin; Neuropathy: Trauma
Injury.1      0.034
## Omitting Rutten et al., 2018; Pregabalin; Neuropathy: Trauma
Injury.2      0.008
## Omitting Rutten et al., 2018; Pregabalin; Neuropathy: Diabetic-
induced    0.004
## Omitting Rutten et al., 2018; Pregabalin; Neuropathy: Diabetic-
induced.1  0.011
## Omitting Rutten et al., 2018; Pregabalin; Neuropathy: Diabetic-
induced.2  0.017
## Omitting Rutten et al., 2018; Pregabalin; Neuropathy: Diabetic-
induced.3  0.025
## Omitting Rutten et al., 2018; Tramadol; Neuropathy: Diabetic-
induced       0.104
## Omitting Rutten et al., 2018; Tramadol; Neuropathy: Diabetic-
induced.1     0.025
## Omitting Rutten et al., 2018; Tramadol; Neuropathy: Diabetic-
induced.2     0.101
##
cov.r
## Omitting Andrews et al., 2012; Ibuprofen; Somatic inflammation
1.011
## Omitting Bryden et al., 2015; Celecoxib; Arthropathy
1.030
## Omitting Bryden et al., 2015; Celecoxib; Arthropathy.1
1.029
## Omitting Bryden et al., 2015; Celecoxib; Arthropathy.2
1.031
## Omitting Bryden et al., 2015; Gabapentin ; Arthropathy
1.016
## Omitting Bryden et al., 2015; Gabapentin; Arthropathy
1.023
## Omitting Bryden et al., 2015; Gabapentin; Arthropathy.1
1.023
## Omitting Bryden et al., 2015; Ibuprofen; Arthropathy
1.033
## Omitting Bryden et al., 2015; Ibuprofen; Arthropathy.1
1.033
## Omitting Bryden et al., 2015; Ibuprofen; Arthropathy.2
1.031
## Omitting Bryden et al., 2015; Morphine; Arthropathy
1.027
## Omitting Bryden et al., 2015; Morphine; Arthropathy.1
1.025
```

```
## Omitting Bryden et al., 2015; Morphine; Arthropathy.2
1.026
## Omitting Bryden et al., 2015; Morphine; Arthropathy.3
1.026
## Omitting Georgieva et al., 2019; Pregabalin; Spinal cord injury
0.958
## Omitting Georgieva et al., 2019; Pregabalin; Spinal cord injury.1
0.998
## Omitting Gould et al., 2016; Celecoxib; Somatic inflammation
1.032
## Omitting Gould et al., 2016; Celecoxib; Somatic inflammation.1
1.035
## Omitting Gould et al., 2016; Celecoxib; Somatic inflammation.2
1.023
## Omitting Gould et al., 2016; Gabapentin; Somatic inflammation
1.023
## Omitting Gould et al., 2016; Gabapentin; Somatic inflammation.1
0.966
## Omitting Gould et al., 2016; Gabapentin; Somatic inflammation.2
1.020
## Omitting Gould et al., 2016; Ibuprofen; Somatic inflammation
0.993
## Omitting Gould et al., 2016; Indomethacin; Somatic inflammation
1.040
## Omitting Gould et al., 2016; Indomethacin; Somatic inflammation.1
1.039
## Omitting Gould et al., 2016; Indomethacin; Somatic inflammation.2
1.040
## Omitting Gould et al., 2016; Morphine; Somatic inflammation
1.035
## Omitting Gould et al., 2016; Morphine; Somatic inflammation.1
1.033
## Omitting Gould et al., 2016; Morphine; Somatic inflammation.2
1.023
## Omitting Gould et al., 2016; Tramadol; Somatic inflammation
1.031
## Omitting Gould et al., 2016; Tramadol; Somatic inflammation.1
1.007
## Omitting Gould et al., 2016; Tramadol; Somatic inflammation.2
0.982
## Omitting Katri et al., 2019; Naproxen; Arthropathy
1.010
## Omitting Lau et al., 2013; Pregabalin; Neuropathy: Trauma Injury
0.982
## Omitting Rutten et al., 2014; Ibuprofen; Arthropathy
0.988
```

```
## Omitting Rutten et al., 2014; Ibuprofen; Arthropathy.1
0.982
## Omitting Rutten et al., 2014; Morphine; Arthropathy
1.016
## Omitting Rutten et al., 2014; Morphine; Arthropathy.1
1.022
## Omitting Rutten et al., 2014; Morphine; Arthropathy.2
1.026
## Omitting Rutten et al., 2014; Naproxen; Arthropathy
0.993
## Omitting Rutten et al., 2014; Naproxen; Arthropathy.1
1.033
## Omitting Rutten et al., 2014; Naproxen; Arthropathy.2
1.033
## Omitting Rutten et al., 2014; Naproxen; Arthropathy.3
1.028
## Omitting Rutten et al., 2014; Naproxen; Arthropathy.4
1.015
## Omitting Rutten et al., 2014; Pregabalin; Arthropathy
1.017
## Omitting Rutten et al., 2014; Pregabalin; Arthropathy.1
1.038
## Omitting Rutten et al., 2014; Pregabalin; Arthropathy.2
1.027
## Omitting Rutten et al., 2014; Pregabalin; Arthropathy.3
1.017
## Omitting Rutten et al., 2014; Pregabalin; Arthropathy.4
1.021
## Omitting Rutten et al., 2018; Gabapentin; Neuropathy: Trauma
Injury      1.038
## Omitting Rutten et al., 2018; Gabapentin; Neuropathy: Trauma
Injury.1    1.002
## Omitting Rutten et al., 2018; Gabapentin; Neuropathy: Diabetic-
induced   1.010
## Omitting Rutten et al., 2018; Gabapentin; Neuropathy: Diabetic-
induced.1 1.039
## Omitting Rutten et al., 2018; Morphine; Neuropathy: Trauma Injury
1.014
## Omitting Rutten et al., 2018; Morphine; Neuropathy: Trauma
Injury.1      1.039
## Omitting Rutten et al., 2018; Morphine; Neuropathy: Trauma
Injury.2      1.039
## Omitting Rutten et al., 2018; Morphine; Neuropathy: Diabetic-
induced     1.035
## Omitting Rutten et al., 2018; Morphine; Neuropathy: Diabetic-
induced.1   1.020
```

```
## Omitting Rutten et al., 2018; Morphine; Neuropathy: Diabetic-induced.2   1.030
## Omitting Rutten et al., 2018; Pregabalin; Neuropathy: Trauma Injury      1.004
## Omitting Rutten et al., 2018; Pregabalin; Neuropathy: Trauma Injury.1    0.985
## Omitting Rutten et al., 2018; Pregabalin; Neuropathy: Trauma Injury.2    1.029
## Omitting Rutten et al., 2018; Pregabalin; Neuropathy: Diabetic-induced   1.040
## Omitting Rutten et al., 2018; Pregabalin; Neuropathy: Diabetic-induced.1 1.030
## Omitting Rutten et al., 2018; Pregabalin; Neuropathy: Diabetic-induced.2 1.023
## Omitting Rutten et al., 2018; Pregabalin; Neuropathy: Diabetic-induced.3 1.011
## Omitting Rutten et al., 2018; Tramadol; Neuropathy: Diabetic-induced     0.889
## Omitting Rutten et al., 2018; Tramadol; Neuropathy: Diabetic-induced.1   1.003
## Omitting Rutten et al., 2018; Tramadol; Neuropathy: Diabetic-induced.2   0.892
##
QE.del
## Omitting Andrews et al., 2012; Ibuprofen; Somatic inflammation
144.295
## Omitting Bryden et al., 2015; Celecoxib; Arthropathy
147.056
## Omitting Bryden et al., 2015; Celecoxib; Arthropathy.1
147.065
## Omitting Bryden et al., 2015; Celecoxib; Arthropathy.2
147.146
## Omitting Bryden et al., 2015; Gabapentin ; Arthropathy
145.649
## Omitting Bryden et al., 2015; Gabapentin; Arthropathy
146.399
## Omitting Bryden et al., 2015; Gabapentin; Arthropathy.1
146.389
## Omitting Bryden et al., 2015; Ibuprofen; Arthropathy
147.144
## Omitting Bryden et al., 2015; Ibuprofen; Arthropathy.1
147.102
## Omitting Bryden et al., 2015; Ibuprofen; Arthropathy.2
146.902
## Omitting Bryden et al., 2015; Morphine; Arthropathy
147.132
```

```
## Omitting Bryden et al., 2015; Morphine; Arthropathy.1
146.885
## Omitting Bryden et al., 2015; Morphine; Arthropathy.2
147.079
## Omitting Bryden et al., 2015; Morphine; Arthropathy.3
147.092
## Omitting Georgieva et al., 2019; Pregabalin; Spinal cord injury
139.350
## Omitting Georgieva et al., 2019; Pregabalin; Spinal cord injury.1
142.966
## Omitting Gould et al., 2016; Celecoxib; Somatic inflammation
146.867
## Omitting Gould et al., 2016; Celecoxib; Somatic inflammation.1
147.059
## Omitting Gould et al., 2016; Celecoxib; Somatic inflammation.2
145.819
## Omitting Gould et al., 2016; Gabapentin; Somatic inflammation
146.130
## Omitting Gould et al., 2016; Gabapentin; Somatic inflammation.1
140.703
## Omitting Gould et al., 2016; Gabapentin; Somatic inflammation.2
145.861
## Omitting Gould et al., 2016; Ibuprofen; Somatic inflammation
142.551
## Omitting Gould et al., 2016; Indomethacin; Somatic inflammation
147.125
## Omitting Gould et al., 2016; Indomethacin; Somatic inflammation.1
146.892
## Omitting Gould et al., 2016; Indomethacin; Somatic inflammation.2
146.992
## Omitting Gould et al., 2016; Morphine; Somatic inflammation
147.077
## Omitting Gould et al., 2016; Morphine; Somatic inflammation.1
146.908
## Omitting Gould et al., 2016; Morphine; Somatic inflammation.2
145.852
## Omitting Gould et al., 2016; Tramadol; Somatic inflammation
147.148
## Omitting Gould et al., 2016; Tramadol; Somatic inflammation.1
144.877
## Omitting Gould et al., 2016; Tramadol; Somatic inflammation.2
142.386
## Omitting Katri et al., 2019; Naproxen; Arthropathy
144.791
## Omitting Lau et al., 2013; Pregabalin; Neuropathy: Trauma Injury
140.659
```

```
## Omitting Rutten et al., 2014; Ibuprofen; Arthropathy
142.821
## Omitting Rutten et al., 2014; Ibuprofen; Arthropathy.1
142.235
## Omitting Rutten et al., 2014; Morphine; Arthropathy
145.763
## Omitting Rutten et al., 2014; Morphine; Arthropathy.1
146.287
## Omitting Rutten et al., 2014; Morphine; Arthropathy.2
146.580
## Omitting Rutten et al., 2014; Naproxen; Arthropathy
143.242
## Omitting Rutten et al., 2014; Naproxen; Arthropathy.1
146.789
## Omitting Rutten et al., 2014; Naproxen; Arthropathy.2
147.122
## Omitting Rutten et al., 2014; Naproxen; Arthropathy.3
146.749
## Omitting Rutten et al., 2014; Naproxen; Arthropathy.4
145.666
## Omitting Rutten et al., 2014; Pregabalin; Arthropathy
145.464
## Omitting Rutten et al., 2014; Pregabalin; Arthropathy.1
147.122
## Omitting Rutten et al., 2014; Pregabalin; Arthropathy.2
146.724
## Omitting Rutten et al., 2014; Pregabalin; Arthropathy.3
145.820
## Omitting Rutten et al., 2014; Pregabalin; Arthropathy.4
146.169
## Omitting Rutten et al., 2018; Gabapentin; Neuropathy: Trauma
Injury      146.561
## Omitting Rutten et al., 2018; Gabapentin; Neuropathy: Trauma
Injury.1    143.305
## Omitting Rutten et al., 2018; Gabapentin; Neuropathy: Diabetic-
induced   143.408
## Omitting Rutten et al., 2018; Gabapentin; Neuropathy: Diabetic-
induced.1 146.445
## Omitting Rutten et al., 2018; Morphine; Neuropathy: Trauma Injury
144.871
## Omitting Rutten et al., 2018; Morphine; Neuropathy: Trauma
Injury.1       146.909
## Omitting Rutten et al., 2018; Morphine; Neuropathy: Trauma
Injury.2       146.973
## Omitting Rutten et al., 2018; Morphine; Neuropathy: Diabetic-
induced     146.996
```

```
## Omitting Rutten et al., 2018; Morphine; Neuropathy: Diabetic-
induced.1   145.515
## Omitting Rutten et al., 2018; Morphine; Neuropathy: Diabetic-
induced.2   146.474
## Omitting Rutten et al., 2018; Pregabalin; Neuropathy: Trauma
Injury       143.972
## Omitting Rutten et al., 2018; Pregabalin; Neuropathy: Trauma
Injury.1     142.283
## Omitting Rutten et al., 2018; Pregabalin; Neuropathy: Trauma
Injury.2     146.125
## Omitting Rutten et al., 2018; Pregabalin; Neuropathy: Diabetic-
induced   146.579
## Omitting Rutten et al., 2018; Pregabalin; Neuropathy: Diabetic-
induced.1 145.488
## Omitting Rutten et al., 2018; Pregabalin; Neuropathy: Diabetic-
induced.2 144.594
## Omitting Rutten et al., 2018; Pregabalin; Neuropathy: Diabetic-
induced.3 143.491
## Omitting Rutten et al., 2018; Tramadol; Neuropathy: Diabetic-
induced     127.485
## Omitting Rutten et al., 2018; Tramadol; Neuropathy: Diabetic-
induced.1   143.920
## Omitting Rutten et al., 2018; Tramadol; Neuropathy: Diabetic-
induced.2   127.170
##
hat
## Omitting Andrews et al., 2012; Ibuprofen; Somatic inflammation
0.016
## Omitting Bryden et al., 2015; Celecoxib; Arthropathy
0.013
## Omitting Bryden et al., 2015; Celecoxib; Arthropathy.1
0.013
## Omitting Bryden et al., 2015; Celecoxib; Arthropathy.2
0.013
## Omitting Bryden et al., 2015; Gabapentin ; Arthropathy
0.013
## Omitting Bryden et al., 2015; Gabapentin; Arthropathy
0.013
## Omitting Bryden et al., 2015; Gabapentin; Arthropathy.1
0.013
## Omitting Bryden et al., 2015; Ibuprofen; Arthropathy
0.014
## Omitting Bryden et al., 2015; Ibuprofen; Arthropathy.1
0.014
## Omitting Bryden et al., 2015; Ibuprofen; Arthropathy.2
0.014
```

```
## Omitting Bryden et al., 2015; Morphine; Arthropathy
0.011
## Omitting Bryden et al., 2015; Morphine; Arthropathy.1
0.012
## Omitting Bryden et al., 2015; Morphine; Arthropathy.2
0.011
## Omitting Bryden et al., 2015; Morphine; Arthropathy.3
0.011
## Omitting Georgieva et al., 2019; Pregabalin; Spinal cord injury
0.015
## Omitting Georgieva et al., 2019; Pregabalin; Spinal cord injury.1
0.016
## Omitting Gould et al., 2016; Celecoxib; Somatic inflammation
0.015
## Omitting Gould et al., 2016; Celecoxib; Somatic inflammation.1
0.015
## Omitting Gould et al., 2016; Celecoxib; Somatic inflammation.2
0.016
## Omitting Gould et al., 2016; Gabapentin; Somatic inflammation
0.014
## Omitting Gould et al., 2016; Gabapentin; Somatic inflammation.1
0.013
## Omitting Gould et al., 2016; Gabapentin; Somatic inflammation.2
0.014
## Omitting Gould et al., 2016; Ibuprofen; Somatic inflammation
0.016
## Omitting Gould et al., 2016; Indomethacin; Somatic inflammation
0.017
## Omitting Gould et al., 2016; Indomethacin; Somatic inflammation.1
0.018
## Omitting Gould et al., 2016; Indomethacin; Somatic inflammation.2
0.017
## Omitting Gould et al., 2016; Morphine; Somatic inflammation
0.015
## Omitting Gould et al., 2016; Morphine; Somatic inflammation.1
0.015
## Omitting Gould et al., 2016; Morphine; Somatic inflammation.2
0.015
## Omitting Gould et al., 2016; Tramadol; Somatic inflammation
0.013
## Omitting Gould et al., 2016; Tramadol; Somatic inflammation.1
0.013
## Omitting Gould et al., 2016; Tramadol; Somatic inflammation.2
0.012
## Omitting Katri et al., 2019; Naproxen; Arthropathy
0.015
```

```
## Omitting Lau et al., 2013; Pregabalin; Neuropathy: Trauma Injury
0.018
## Omitting Rutten et al., 2014; Ibuprofen; Arthropathy
0.012
## Omitting Rutten et al., 2014; Ibuprofen; Arthropathy.1
0.012
## Omitting Rutten et al., 2014; Morphine; Arthropathy
0.012
## Omitting Rutten et al., 2014; Morphine; Arthropathy.1
0.013
## Omitting Rutten et al., 2014; Morphine; Arthropathy.2
0.013
## Omitting Rutten et al., 2014; Naproxen; Arthropathy
0.013
## Omitting Rutten et al., 2014; Naproxen; Arthropathy.1
0.015
## Omitting Rutten et al., 2014; Naproxen; Arthropathy.2
0.014
## Omitting Rutten et al., 2014; Naproxen; Arthropathy.3
0.013
## Omitting Rutten et al., 2014; Naproxen; Arthropathy.4
0.012
## Omitting Rutten et al., 2014; Pregabalin; Arthropathy
0.014
## Omitting Rutten et al., 2014; Pregabalin; Arthropathy.1
0.016
## Omitting Rutten et al., 2014; Pregabalin; Arthropathy.2
0.013
## Omitting Rutten et al., 2014; Pregabalin; Arthropathy.3
0.012
## Omitting Rutten et al., 2014; Pregabalin; Arthropathy.4
0.013
## Omitting Rutten et al., 2018; Gabapentin; Neuropathy: Trauma
Injury      0.018
## Omitting Rutten et al., 2018; Gabapentin; Neuropathy: Trauma
Injury.1    0.016
## Omitting Rutten et al., 2018; Gabapentin; Neuropathy: Diabetic-
induced   0.019
## Omitting Rutten et al., 2018; Gabapentin; Neuropathy: Diabetic-
induced.1 0.019
## Omitting Rutten et al., 2018; Morphine; Neuropathy: Trauma Injury
0.015
## Omitting Rutten et al., 2018; Morphine; Neuropathy: Trauma
Injury.1      0.017
## Omitting Rutten et al., 2018; Morphine; Neuropathy: Trauma
Injury.2      0.017
```

```
## Omitting Rutten et al., 2018; Morphine; Neuropathy: Diabetic-
induced     0.015
## Omitting Rutten et al., 2018; Morphine; Neuropathy: Diabetic-
induced.1   0.016
## Omitting Rutten et al., 2018; Morphine; Neuropathy: Diabetic-
induced.2   0.016
## Omitting Rutten et al., 2018; Pregabalin; Neuropathy: Trauma
Injury      0.015
## Omitting Rutten et al., 2018; Pregabalin; Neuropathy: Trauma
Injury.1    0.014
## Omitting Rutten et al., 2018; Pregabalin; Neuropathy: Trauma
Injury.2    0.016
## Omitting Rutten et al., 2018; Pregabalin; Neuropathy: Diabetic-
induced   0.019
## Omitting Rutten et al., 2018; Pregabalin; Neuropathy: Diabetic-
induced.1 0.019
## Omitting Rutten et al., 2018; Pregabalin; Neuropathy: Diabetic-
induced.2 0.020
## Omitting Rutten et al., 2018; Pregabalin; Neuropathy: Diabetic-
induced.3 0.019
## Omitting Rutten et al., 2018; Tramadol; Neuropathy: Diabetic-
induced     0.007
## Omitting Rutten et al., 2018; Tramadol; Neuropathy: Diabetic-
induced.1   0.015
## Omitting Rutten et al., 2018; Tramadol; Neuropathy: Diabetic-
induced.2   0.007
##
weight
## Omitting Andrews et al., 2012; Ibuprofen; Somatic inflammation
1.637
## Omitting Bryden et al., 2015; Celecoxib; Arthropathy
1.309
## Omitting Bryden et al., 2015; Celecoxib; Arthropathy.1
1.258
## Omitting Bryden et al., 2015; Celecoxib; Arthropathy.2
1.281
## Omitting Bryden et al., 2015; Gabapentin ; Arthropathy
1.314
## Omitting Bryden et al., 2015; Gabapentin; Arthropathy
1.323
## Omitting Bryden et al., 2015; Gabapentin; Arthropathy.1
1.323
## Omitting Bryden et al., 2015; Ibuprofen; Arthropathy
1.382
## Omitting Bryden et al., 2015; Ibuprofen; Arthropathy.1
1.391
```

```
## Omitting Bryden et al., 2015; Ibuprofen; Arthropathy.2
1.403
## Omitting Bryden et al., 2015; Morphine; Arthropathy
1.119
## Omitting Bryden et al., 2015; Morphine; Arthropathy.1
1.159
## Omitting Bryden et al., 2015; Morphine; Arthropathy.2
1.106
## Omitting Bryden et al., 2015; Morphine; Arthropathy.3
1.108
## Omitting Georgieva et al., 2019; Pregabalin; Spinal cord injury
1.467
## Omitting Georgieva et al., 2019; Pregabalin; Spinal cord injury.1
1.622
## Omitting Gould et al., 2016; Celecoxib; Somatic inflammation
1.458
## Omitting Gould et al., 2016; Celecoxib; Somatic inflammation.1
1.524
## Omitting Gould et al., 2016; Celecoxib; Somatic inflammation.2
1.572
## Omitting Gould et al., 2016; Gabapentin; Somatic inflammation
1.408
## Omitting Gould et al., 2016; Gabapentin; Somatic inflammation.1
1.296
## Omitting Gould et al., 2016; Gabapentin; Somatic inflammation.2
1.425
## Omitting Gould et al., 2016; Ibuprofen; Somatic inflammation
1.608
## Omitting Gould et al., 2016; Indomethacin; Somatic inflammation
1.682
## Omitting Gould et al., 2016; Indomethacin; Somatic inflammation.1
1.767
## Omitting Gould et al., 2016; Indomethacin; Somatic inflammation.2
1.735
## Omitting Gould et al., 2016; Morphine; Somatic inflammation
1.522
## Omitting Gould et al., 2016; Morphine; Somatic inflammation.1
1.486
## Omitting Gould et al., 2016; Morphine; Somatic inflammation.2
1.538
## Omitting Gould et al., 2016; Tramadol; Somatic inflammation
1.282
## Omitting Gould et al., 2016; Tramadol; Somatic inflammation.1
1.296
## Omitting Gould et al., 2016; Tramadol; Somatic inflammation.2
1.205
```

```
## Omitting Katri et al., 2019; Naproxen; Arthropathy
1.472
## Omitting Lau et al., 2013; Pregabalin; Neuropathy: Trauma Injury
1.825
## Omitting Rutten et al., 2014; Ibuprofen; Arthropathy
1.222
## Omitting Rutten et al., 2014; Ibuprofen; Arthropathy.1
1.179
## Omitting Rutten et al., 2014; Morphine; Arthropathy
1.217
## Omitting Rutten et al., 2014; Morphine; Arthropathy.1
1.263
## Omitting Rutten et al., 2014; Morphine; Arthropathy.2
1.291
## Omitting Rutten et al., 2014; Naproxen; Arthropathy
1.253
## Omitting Rutten et al., 2014; Naproxen; Arthropathy.1
1.525
## Omitting Rutten et al., 2014; Naproxen; Arthropathy.2
1.388
## Omitting Rutten et al., 2014; Naproxen; Arthropathy.3
1.308
## Omitting Rutten et al., 2014; Naproxen; Arthropathy.4
1.209
## Omitting Rutten et al., 2014; Pregabalin; Arthropathy
1.415
## Omitting Rutten et al., 2014; Pregabalin; Arthropathy.1
1.596
## Omitting Rutten et al., 2014; Pregabalin; Arthropathy.2
1.306
## Omitting Rutten et al., 2014; Pregabalin; Arthropathy.3
1.222
## Omitting Rutten et al., 2014; Pregabalin; Arthropathy.4
1.252
## Omitting Rutten et al., 2018; Gabapentin; Neuropathy: Trauma
Injury       1.814
## Omitting Rutten et al., 2018; Gabapentin; Neuropathy: Trauma
Injury.1     1.645
## Omitting Rutten et al., 2018; Gabapentin; Neuropathy: Diabetic-
induced    1.924
## Omitting Rutten et al., 2018; Gabapentin; Neuropathy: Diabetic-
induced.1  1.930
## Omitting Rutten et al., 2018; Morphine; Neuropathy: Trauma Injury
1.526
## Omitting Rutten et al., 2018; Morphine; Neuropathy: Trauma
Injury.1       1.740
```

```
## Omitting Rutten et al., 2018; Morphine; Neuropathy: Trauma
Injury.2       1.680
## Omitting Rutten et al., 2018; Morphine; Neuropathy: Diabetic-
induced      1.547
## Omitting Rutten et al., 2018; Morphine; Neuropathy: Diabetic-
induced.1    1.554
## Omitting Rutten et al., 2018; Morphine; Neuropathy: Diabetic-
induced.2    1.557
## Omitting Rutten et al., 2018; Pregabalin; Neuropathy: Trauma
Injury       1.468
## Omitting Rutten et al., 2018; Pregabalin; Neuropathy: Trauma
Injury.1     1.360
## Omitting Rutten et al., 2018; Pregabalin; Neuropathy: Trauma
Injury.2     1.609
## Omitting Rutten et al., 2018; Pregabalin; Neuropathy: Diabetic-
induced    1.928
## Omitting Rutten et al., 2018; Pregabalin; Neuropathy: Diabetic-
induced.1  1.933
## Omitting Rutten et al., 2018; Pregabalin; Neuropathy: Diabetic-
induced.2  1.977
## Omitting Rutten et al., 2018; Pregabalin; Neuropathy: Diabetic-
induced.3  1.934
## Omitting Rutten et al., 2018; Tramadol; Neuropathy: Diabetic-
induced      0.715
## Omitting Rutten et al., 2018; Tramadol; Neuropathy: Diabetic-
induced.1    1.533
## Omitting Rutten et al., 2018; Tramadol; Neuropathy: Diabetic-
induced.2    0.677
##
infl
## Omitting Andrews et al., 2012; Ibuprofen; Somatic inflammation
## Omitting Bryden et al., 2015; Celecoxib; Arthropathy
## Omitting Bryden et al., 2015; Celecoxib; Arthropathy.1
## Omitting Bryden et al., 2015; Celecoxib; Arthropathy.2
## Omitting Bryden et al., 2015; Gabapentin ; Arthropathy
## Omitting Bryden et al., 2015; Gabapentin; Arthropathy
## Omitting Bryden et al., 2015; Gabapentin; Arthropathy.1
## Omitting Bryden et al., 2015; Ibuprofen; Arthropathy
## Omitting Bryden et al., 2015; Ibuprofen; Arthropathy.1
## Omitting Bryden et al., 2015; Ibuprofen; Arthropathy.2
## Omitting Bryden et al., 2015; Morphine; Arthropathy
## Omitting Bryden et al., 2015; Morphine; Arthropathy.1
## Omitting Bryden et al., 2015; Morphine; Arthropathy.2
## Omitting Bryden et al., 2015; Morphine; Arthropathy.3
## Omitting Georgieva et al., 2019; Pregabalin; Spinal cord injury
## Omitting Georgieva et al., 2019; Pregabalin; Spinal cord injury.1
```

```
## Omitting Gould et al., 2016; Celecoxib; Somatic inflammation
## Omitting Gould et al., 2016; Celecoxib; Somatic inflammation.1
## Omitting Gould et al., 2016; Celecoxib; Somatic inflammation.2
## Omitting Gould et al., 2016; Gabapentin; Somatic inflammation
## Omitting Gould et al., 2016; Gabapentin; Somatic inflammation.1
## Omitting Gould et al., 2016; Gabapentin; Somatic inflammation.2
## Omitting Gould et al., 2016; Ibuprofen; Somatic inflammation
## Omitting Gould et al., 2016; Indomethacin; Somatic inflammation
## Omitting Gould et al., 2016; Indomethacin; Somatic inflammation.1
## Omitting Gould et al., 2016; Indomethacin; Somatic inflammation.2
## Omitting Gould et al., 2016; Morphine; Somatic inflammation
## Omitting Gould et al., 2016; Morphine; Somatic inflammation.1
## Omitting Gould et al., 2016; Morphine; Somatic inflammation.2
## Omitting Gould et al., 2016; Tramadol; Somatic inflammation
## Omitting Gould et al., 2016; Tramadol; Somatic inflammation.1
## Omitting Gould et al., 2016; Tramadol; Somatic inflammation.2
## Omitting Katri et al., 2019; Naproxen; Arthropathy
## Omitting Lau et al., 2013; Pregabalin; Neuropathy: Trauma Injury
## Omitting Rutten et al., 2014; Ibuprofen; Arthropathy
## Omitting Rutten et al., 2014; Ibuprofen; Arthropathy.1
## Omitting Rutten et al., 2014; Morphine; Arthropathy
## Omitting Rutten et al., 2014; Morphine; Arthropathy.1
## Omitting Rutten et al., 2014; Morphine; Arthropathy.2
## Omitting Rutten et al., 2014; Naproxen; Arthropathy
## Omitting Rutten et al., 2014; Naproxen; Arthropathy.1
## Omitting Rutten et al., 2014; Naproxen; Arthropathy.2
## Omitting Rutten et al., 2014; Naproxen; Arthropathy.3
## Omitting Rutten et al., 2014; Naproxen; Arthropathy.4
## Omitting Rutten et al., 2014; Pregabalin; Arthropathy
## Omitting Rutten et al., 2014; Pregabalin; Arthropathy.1
## Omitting Rutten et al., 2014; Pregabalin; Arthropathy.2
## Omitting Rutten et al., 2014; Pregabalin; Arthropathy.3
## Omitting Rutten et al., 2014; Pregabalin; Arthropathy.4
## Omitting Rutten et al., 2018; Gabapentin; Neuropathy: Trauma
Injury
## Omitting Rutten et al., 2018; Gabapentin; Neuropathy: Trauma
Injury.1
## Omitting Rutten et al., 2018; Gabapentin; Neuropathy: Diabetic-
induced
## Omitting Rutten et al., 2018; Gabapentin; Neuropathy: Diabetic-
induced.1
## Omitting Rutten et al., 2018; Morphine; Neuropathy: Trauma Injury
## Omitting Rutten et al., 2018; Morphine; Neuropathy: Trauma
Injury.1
## Omitting Rutten et al., 2018; Morphine; Neuropathy: Trauma
Injury.2
```

```
## Omitting Rutten et al., 2018; Morphine; Neuropathy: Diabetic-
induced
## Omitting Rutten et al., 2018; Morphine; Neuropathy: Diabetic-
induced.1
## Omitting Rutten et al., 2018; Morphine; Neuropathy: Diabetic-
induced.2
## Omitting Rutten et al., 2018; Pregabalin; Neuropathy: Trauma
Injury
## Omitting Rutten et al., 2018; Pregabalin; Neuropathy: Trauma
Injury.1
## Omitting Rutten et al., 2018; Pregabalin; Neuropathy: Trauma
Injury.2
## Omitting Rutten et al., 2018; Pregabalin; Neuropathy: Diabetic-
induced
## Omitting Rutten et al., 2018; Pregabalin; Neuropathy: Diabetic-
induced.1
## Omitting Rutten et al., 2018; Pregabalin; Neuropathy: Diabetic-
induced.2
## Omitting Rutten et al., 2018; Pregabalin; Neuropathy: Diabetic-
induced.3
## Omitting Rutten et al., 2018; Tramadol; Neuropathy: Diabetic-
induced
## Omitting Rutten et al., 2018; Tramadol; Neuropathy: Diabetic-
induced.1
## Omitting Rutten et al., 2018; Tramadol; Neuropathy: Diabetic-
induced.2
##
##
## Baujat Diagnostics (sorted by Heterogeneity Contribution)
##  -------------------------------------------------------
##
HetContrib
## Omitting Rutten et al., 2018; Tramadol; Neuropathy: Diabetic-
induced.2       19.897
## Omitting Rutten et al., 2018; Tramadol; Neuropathy: Diabetic-
induced         19.578
## Omitting Georgieva et al., 2019; Pregabalin; Spinal cord injury
7.693
## Omitting Gould et al., 2016; Gabapentin; Somatic inflammation.1
6.377
## Omitting Lau et al., 2013; Pregabalin; Neuropathy: Trauma Injury
6.345
## Omitting Rutten et al., 2014; Ibuprofen; Arthropathy.1
4.871
## Omitting Rutten et al., 2018; Pregabalin; Neuropathy: Trauma
Injury.1         4.810
```

```
## Omitting Gould et al., 2016; Tramadol; Somatic inflammation.2
4.720
## Omitting Gould et al., 2016; Ibuprofen; Somatic inflammation
4.523
## Omitting Rutten et al., 2014; Ibuprofen; Arthropathy
4.288
## Omitting Georgieva et al., 2019; Pregabalin; Spinal cord injury.1
4.114
## Omitting Rutten et al., 2014; Naproxen; Arthropathy
3.869
## Omitting Rutten et al., 2018; Gabapentin; Neuropathy: Trauma
Injury.1         3.779
## Omitting Rutten et al., 2018; Gabapentin; Neuropathy: Diabetic-
induced        3.645
## Omitting Rutten et al., 2018; Pregabalin; Neuropathy: Diabetic-
induced.3      3.563
## Omitting Rutten et al., 2018; Tramadol; Neuropathy: Diabetic-
induced.1        3.183
## Omitting Rutten et al., 2018; Pregabalin; Neuropathy: Trauma
Injury           3.136
## Omitting Andrews et al., 2012; Ibuprofen; Somatic inflammation
2.806
## Omitting Rutten et al., 2018; Pregabalin; Neuropathy: Diabetic-
induced.2      2.485
## Omitting Katri et al., 2019; Naproxen; Arthropathy
2.328
## Omitting Gould et al., 2016; Tramadol; Somatic inflammation.1
2.250
## Omitting Rutten et al., 2018; Morphine; Neuropathy: Trauma Injury
2.247
## Omitting Rutten et al., 2014; Pregabalin; Arthropathy
1.665
## Omitting Rutten et al., 2018; Pregabalin; Neuropathy: Diabetic-
induced.1      1.619
## Omitting Rutten et al., 2018; Morphine; Neuropathy: Diabetic-
induced.1        1.611
## Omitting Bryden et al., 2015; Gabapentin ; Arthropathy
1.485
## Omitting Rutten et al., 2014; Naproxen; Arthropathy.4
1.472
## Omitting Rutten et al., 2014; Morphine; Arthropathy
1.375
## Omitting Rutten et al., 2014; Pregabalin; Arthropathy.3
1.318
## Omitting Gould et al., 2016; Celecoxib; Somatic inflammation.2
1.311
```

```
## Omitting Gould et al., 2016; Morphine; Somatic inflammation.2
1.280
## Omitting Gould et al., 2016; Gabapentin; Somatic inflammation.2
1.274
## Omitting Gould et al., 2016; Gabapentin; Somatic inflammation
1.009
## Omitting Rutten et al., 2018; Pregabalin; Neuropathy: Trauma
Injury.2         1.009
## Omitting Rutten et al., 2014; Pregabalin; Arthropathy.4
0.972
## Omitting Rutten et al., 2014; Morphine; Arthropathy.1
0.855
## Omitting Bryden et al., 2015; Gabapentin; Arthropathy.1
0.754
## Omitting Bryden et al., 2015; Gabapentin; Arthropathy
0.744
## Omitting Rutten et al., 2018; Gabapentin; Neuropathy: Diabetic-
induced.1      0.688
## Omitting Rutten et al., 2018; Morphine; Neuropathy: Diabetic-
induced.2        0.667
## Omitting Rutten et al., 2018; Gabapentin; Neuropathy: Trauma
Injury           0.578
## Omitting Rutten et al., 2014; Morphine; Arthropathy.2
0.566
## Omitting Rutten et al., 2018; Pregabalin; Neuropathy: Diabetic-
induced        0.558
## Omitting Rutten et al., 2014; Pregabalin; Arthropathy.2
0.423
## Omitting Rutten et al., 2014; Naproxen; Arthropathy.3
0.398
## Omitting Rutten et al., 2014; Naproxen; Arthropathy.1
0.357
## Omitting Gould et al., 2016; Celecoxib; Somatic inflammation
0.281
## Omitting Bryden et al., 2015; Morphine; Arthropathy.1
0.265
## Omitting Gould et al., 2016; Indomethacin; Somatic inflammation.1
0.254
## Omitting Bryden et al., 2015; Ibuprofen; Arthropathy.2
0.247
## Omitting Gould et al., 2016; Morphine; Somatic inflammation.1
0.241
## Omitting Rutten et al., 2018; Morphine; Neuropathy: Trauma
Injury.1           0.238
## Omitting Rutten et al., 2018; Morphine; Neuropathy: Trauma
Injury.2           0.175
```

```
## Omitting Gould et al., 2016; Indomethacin; Somatic inflammation.2
0.157
## Omitting Rutten et al., 2018; Morphine; Neuropathy: Diabetic-
induced          0.153
## Omitting Bryden et al., 2015; Celecoxib; Arthropathy
0.094
## Omitting Gould et al., 2016; Celecoxib; Somatic inflammation.1
0.092
## Omitting Bryden et al., 2015; Celecoxib; Arthropathy.1
0.086
## Omitting Gould et al., 2016; Morphine; Somatic inflammation
0.074
## Omitting Bryden et al., 2015; Morphine; Arthropathy.2
0.072
## Omitting Bryden et al., 2015; Morphine; Arthropathy.3
0.060
## Omitting Bryden et al., 2015; Ibuprofen; Arthropathy.1
0.049
## Omitting Rutten et al., 2014; Naproxen; Arthropathy.2
0.029
## Omitting Rutten et al., 2014; Pregabalin; Arthropathy.1
0.029
## Omitting Gould et al., 2016; Indomethacin; Somatic inflammation
0.026
## Omitting Bryden et al., 2015; Morphine; Arthropathy
0.020
## Omitting Bryden et al., 2015; Ibuprofen; Arthropathy
0.008
## Omitting Bryden et al., 2015; Celecoxib; Arthropathy.2
0.006
## Omitting Gould et al., 2016; Tramadol; Somatic inflammation
0.003
##
InfluenceEffectSize
## Omitting Rutten et al., 2018; Tramadol; Neuropathy: Diabetic-
induced.2                 0.084
## Omitting Rutten et al., 2018; Tramadol; Neuropathy: Diabetic-
induced                   0.089
## Omitting Georgieva et al., 2019; Pregabalin; Spinal cord injury
0.109
## Omitting Gould et al., 2016; Gabapentin; Somatic inflammation.1
0.071
## Omitting Lau et al., 2013; Pregabalin; Neuropathy: Trauma Injury
0.148
## Omitting Rutten et al., 2014; Ibuprofen; Arthropathy.1
0.046
```

```
## Omitting Rutten et al., 2018; Pregabalin; Neuropathy: Trauma
Injury.1                    0.059
## Omitting Gould et al., 2016; Tramadol; Somatic inflammation.2
0.046
## Omitting Gould et al., 2016; Ibuprofen; Somatic inflammation
0.078
## Omitting Rutten et al., 2014; Ibuprofen; Arthropathy
0.043
## Omitting Georgieva et al., 2019; Pregabalin; Spinal cord injury.1
0.072
## Omitting Rutten et al., 2014; Naproxen; Arthropathy
0.041
## Omitting Rutten et al., 2018; Gabapentin; Neuropathy: Trauma
Injury.1                    0.068
## Omitting Rutten et al., 2018; Gabapentin; Neuropathy: Diabetic-
induced                   0.099
## Omitting Rutten et al., 2018; Pregabalin; Neuropathy: Diabetic-
induced.3                 0.098
## Omitting Rutten et al., 2018; Tramadol; Neuropathy: Diabetic-
induced.1                   0.049
## Omitting Rutten et al., 2018; Pregabalin; Neuropathy: Trauma
Injury                      0.044
## Omitting Andrews et al., 2012; Ibuprofen; Somatic inflammation
0.050
## Omitting Rutten et al., 2018; Pregabalin; Neuropathy: Diabetic-
induced.2                 0.073
## Omitting Katri et al., 2019; Naproxen; Arthropathy
0.033
## Omitting Gould et al., 2016; Tramadol; Somatic inflammation.1
0.025
## Omitting Rutten et al., 2018; Morphine; Neuropathy: Trauma Injury
0.034
## Omitting Rutten et al., 2014; Pregabalin; Arthropathy
0.022
## Omitting Rutten et al., 2018; Pregabalin; Neuropathy: Diabetic-
induced.1                 0.044
## Omitting Rutten et al., 2018; Morphine; Neuropathy: Diabetic-
induced.1                   0.026
## Omitting Bryden et al., 2015; Gabapentin ; Arthropathy
0.017
## Omitting Rutten et al., 2014; Naproxen; Arthropathy.4
0.015
## Omitting Rutten et al., 2014; Morphine; Arthropathy
0.014
## Omitting Rutten et al., 2014; Pregabalin; Arthropathy.3
0.013
```

```
## Omitting Gould et al., 2016; Celecoxib; Somatic inflammation.2
0.021
## Omitting Gould et al., 2016; Morphine; Somatic inflammation.2
0.020
## Omitting Gould et al., 2016; Gabapentin; Somatic inflammation.2
0.017
## Omitting Gould et al., 2016; Gabapentin; Somatic inflammation
0.013
## Omitting Rutten et al., 2018; Pregabalin; Neuropathy: Trauma
Injury.2                  0.017
## Omitting Rutten et al., 2014; Pregabalin; Arthropathy.4
0.010
## Omitting Rutten et al., 2014; Morphine; Arthropathy.1
0.009
## Omitting Bryden et al., 2015; Gabapentin; Arthropathy.1
0.009
## Omitting Bryden et al., 2015; Gabapentin; Arthropathy
0.009
## Omitting Rutten et al., 2018; Gabapentin; Neuropathy: Diabetic-
induced.1                0.019
## Omitting Rutten et al., 2018; Morphine; Neuropathy: Diabetic-
induced.2                  0.011
## Omitting Rutten et al., 2018; Gabapentin; Neuropathy: Trauma
Injury                     0.013
## Omitting Rutten et al., 2014; Morphine; Arthropathy.2
0.006
## Omitting Rutten et al., 2018; Pregabalin; Neuropathy: Diabetic-
induced                  0.015
## Omitting Rutten et al., 2014; Pregabalin; Arthropathy.2
0.005
## Omitting Rutten et al., 2014; Naproxen; Arthropathy.3
0.005
## Omitting Rutten et al., 2014; Naproxen; Arthropathy.1
0.005
## Omitting Gould et al., 2016; Celecoxib; Somatic inflammation
0.004
## Omitting Bryden et al., 2015; Morphine; Arthropathy.1
0.002
## Omitting Gould et al., 2016; Indomethacin; Somatic inflammation.1
0.005
## Omitting Bryden et al., 2015; Ibuprofen; Arthropathy.2
0.003
## Omitting Gould et al., 2016; Morphine; Somatic inflammation.1
0.003
## Omitting Rutten et al., 2018; Morphine; Neuropathy: Trauma
Injury.1                    0.005
```

```
## Omitting Rutten et al., 2018; Morphine; Neuropathy: Trauma
Injury.2                        0.003
## Omitting Gould et al., 2016; Indomethacin; Somatic inflammation.2
0.003
## Omitting Rutten et al., 2018; Morphine; Neuropathy: Diabetic-
induced                         0.002
## Omitting Bryden et al., 2015; Celecoxib; Arthropathy
0.001
## Omitting Gould et al., 2016; Celecoxib; Somatic inflammation.1
0.001
## Omitting Bryden et al., 2015; Celecoxib; Arthropathy.1
0.001
## Omitting Gould et al., 2016; Morphine; Somatic inflammation
0.001
## Omitting Bryden et al., 2015; Morphine; Arthropathy.2
0.001
## Omitting Bryden et al., 2015; Morphine; Arthropathy.3
0.001
## Omitting Bryden et al., 2015; Ibuprofen; Arthropathy.1
0.001
## Omitting Rutten et al., 2014; Naproxen; Arthropathy.2
0.000
## Omitting Rutten et al., 2014; Pregabalin; Arthropathy.1
0.000
## Omitting Gould et al., 2016; Indomethacin; Somatic inflammation
0.001
## Omitting Bryden et al., 2015; Morphine; Arthropathy
0.000
## Omitting Bryden et al., 2015; Ibuprofen; Arthropathy
0.000
## Omitting Bryden et al., 2015; Celecoxib; Arthropathy.2
0.000
## Omitting Gould et al., 2016; Tramadol; Somatic inflammation
0.000
```

S6

Table. The number of cohort-level comparisons and animals for study design characteristics used in mice modelling experiments.

	No. of studies	No. of reports	No. of cohort-level comparisons	No. of animals
**Disease Model**				
Neuropathy: Trauma injury	1	1	5	88
Cancer	4	4	4	109
Migraine	1	1	4	52
Somatic inflammation	1	1	2	34
Visceral inflammation	2	2	2	26
Complex regional pain syndrome	1	1	1	20
Dental injury	1	1	1	18
Lumbar intervertebral disc degeneration	1	1	1	39
Mucositis	1	1	1	16
Procedure-associated pain	1	1	1	11
**Mouse Strain**				
C57BL/6	9	9	10	209

Not reported	1	1	7	104
FVB/NJ	1	1	2	33
B6D2F1	1	1	1	16
Balb/c	1	1	1	33
ICR	1	1	1	18
**Sex**				
Female	8	8	12	188
Male	4	4	8	191
Not reported	2	2	2	34
**Substrate Type**				
Corncob bedding	2	2	12	194
Food pellet	4	4	4	97
Pebble	2	2	2	24
Not reported	2	2	2	26
Sand	1	1	1	33
Bedding material	1	1	1	39
**Burrowing Outcome**				
Amount displaced	11	11	21	402

Latency to burrow	1	1	1	11

S7

Table. The number of cohort-level comparisons and animals for study design characteristics used in mice intervention experiments.

	No. of studies	No. of reports	No. of cohort-level comparisons	No. of animals
**Disease Model**				
Procedure-associated pain	5	5	7	128
Nociplastic pain	1	1	4	44
Cancer	2	2	3	48
Visceral inflammation	1	1	2	30
Complex regional pain syndrome	1	1	1	9
Neuropathy: Trauma injury	1	1	1	16
**Drug Class**				
NSAID	3	3	5	96
Unknown mechanism of action	2	2	4	58
Gabapentinoid	2	2	3	38
Opioid	2	2	2	36
Bradykinin receptor antagonist	1	1	2	22

Antihyperglycaemic	1	1	1	9
Combined therapy	1	1	1	16
**Mouse Strain**				
C57BL/6	9	9	13	215
Swiss	1	1	4	44
Not reported	1	1	1	16
**Sex**				
Female	8	8	12	183
Male	1	1	4	44
Mixed	1	1	1	32
Not reported	1	1	1	16
**Substrate Type**				
Food pellet	6	6	11	172
Pebble	2	2	3	48
Corncob bedding	2	2	2	25
Not reported	1	1	2	30
**Burrowing Outcome**				
Amount displaced	6	6	11	147

Duration of burrowing	3	3	5	96
Latency to burrow	2	2	2	32

S8.

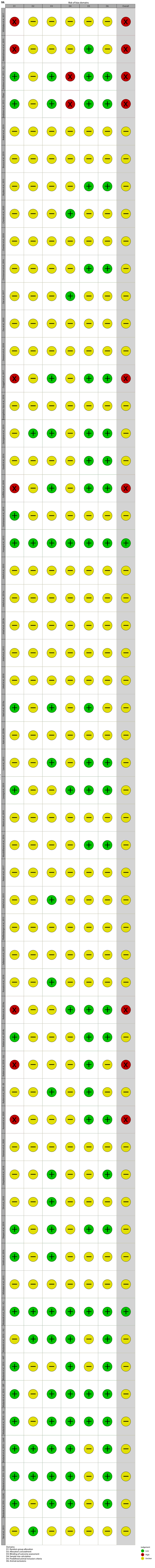
Risk of bias domains
D1
D2
D3
D4
D5
D6
Overall
Study
Domains:
D1: Random group allocation
D2: Allocation concealment
D3: Blinding of outcome assessment
D4: Sample size calculation
D5: Predefined animal inclusion criteria
D6: Animal exclusions
Judgement
Low
High
Unclear

S9

Table. Animal suppliers for experiments conducted in mouse strains.

Experiment Type	Strain	Animal Supplier	No. of k
Animal Modelling	C57BL/6	Jackson Laboratories	3
		Charles River	2
		Animal Resource Centre (Perth, Australia)	2
		Not reported	2
		In-house breeding facility (Fuellinsdorf, Switzerland)	1
	Not reported	Charles River	7
	FVB/NJ	Jackson Laboratories	2
	Balb/c	Envigo	1
	B6D2F1	Not reported	1
	ICR	BioLasco	1
Drug Intervention	C57BL/6	In-house breeding facility (University of Zurich)	5
		Animal Resource Centre (Perth, Australia)	3
		Not reported	3
		Charles River	2
		Jackson Laboratories	1
	Swiss	Not reported	4
	Not reported	Jackson Laboratories	1

Table. Animal suppliers for experiments conducted in rat strains.

Experiment Type	Strain	Animal Supplier	No. of k
Animal Modelling	Sprague Dawley	Charles River	16
		Janvier Laboratories	3
		Animal Resources Centre (Perth, Australia)	2
		Harlan Laboratories	2
		Not reported	2
		Laboratory Animal Services (University of Adelaide)	1
		Taconic	1
	Wistar	Charles River	7
		B&K Universal Ltd.	2
		Harlan Laboratories	1
		In-house breeding facility (University of Santa Maria)	1
	Wistar Hannover	Charles River	10
	LEW/CrlCrlj	Charles River	1
	Lewis	Envigo	1
	Zucker diabetic fatty (fa/fa) obese rat	Charles River	1

Drug Intervention	Sprague Dawley	Charles River	23
		Janvier Laboratories	19
		Harlan Laboratories	6
	Wistar Hannover	Charles River	17
	Zucker diabetic fatty (fa/fa) obese rat	Charles River	10
	Wistar	Charles River	6
		In-house breeding facility (University of Santa Maria)	1
	DA/Arc	Laboratory Animal Services (University of Adelaide)	4
	Lewis	Envigo	3

S10

Table. The N range and its median of each mouse disease models used in animal modelling and drug intervention experiments

Disease Model – Animal Modelling	k	N Range	Median
Neuropathy: Trauma induced	5	15 - 22	18
Cancer	4	9 - 52	24
Migraine	4	12 - 14	13
Somatic inflammation	2	14 - 20	17
Visceral inflammation	2	11 - 15	13
Complex regional pain syndrome	1	20	20
Dental injury	1	18	18
Lumbar intervertebral disc degeneration	1	39	39
Mucositis	1	16	16
Procedure-associated pain	1	11	11

Disease Model – Drug Intervention	k	N Range	Median
Procedure-associated pain	7	16 - 32	16
Nociplastic pain	4	11	11
Cancer	3	14 - 20	14
Visceral inflammation	2	15	15
Complex regional pain syndrome	1	9	9
Neuropathy: Trauma induced	1	16	16

Table. The N range and its median of each rat disease models used in animal modelling and drug intervention experiments

Disease Model – Animal Modelling	k	N Range	Median
Somatic inflammation	19	8 - 32	18.5
Neuropathy: Trauma induced	13	6 - 37	20
Arthropathy	9	13 - 30	19
Procedure-associated pain	3	12 - 26	19
Cancer	1	50	50
Migraine	1	10	10
Mucositis	1	32	32
Neuropathy: Antiretroviral-induced	1	15	15
Neuropathy: Chemotherapy-induced	1	12	12
Neuropathy: Diabetic induced	1	29	29
Ultraviolet B and heat rekindling	1	16	16

Disease Model – Animal Modelling	k	N Range	Median
Arthropathy	35	10 - 22	12
Somatic inflammation	21	11 - 23	14
Neuropathy: Diabetic induced	12	15 - 21	17

Neuropathy: Trauma induced	10	15 - 26	15.5
Spinal cord injury	6	20 - 29	28
Mucositis	4	10	10
Procedure-associated pain	1	16	16

S11

Table. The Number of reports which reported relevant information on acclimatisation, animal husbandry and experimental conditions.

Items reported	Number of reports	%	Data
Animals given a time period to acclimatise	34	61	Median = 56 hr;<br>Range = 1 – 504 hr
Number of animals housed per cage	36	64	Median = 3<br>Range = 1 – 6
Temperature of the housing environment	37	66	Median = 20°C;<br>Range = 19 – 24°C
Humidity of the housing environment	27	48	Median = 45%;<br>Range = 15 – 60%
Noise level of the housing environment	0	0	N/A
Number of light/dark cycle	51	91	Median = 12/12 hr;<br>Range = 11/13 hr – 14/10 hr
In which phase, the burrowing assessment was conducted	42	75	Dark phase = 10 reports<br>Light phase = 32 reports
Experimental lighting condition	14	25	Median = 30 lux;<br>Range = 0.8 – 50 lux
Temperature during the burrowing assessment	11	20	Median = 22°C;<br>Range = 19 – 20°C
Humidity during the burrowing assessment	10	18	Median = 45%;<br>Range = 15 – 50%
Noise level during the burrowing assessment	1	2	Median = 45dB;<br>Range = 45dB
Animals given access to food and/or water during the burrowing assessment	21	38	Food only = 1 report<br>Water only = 1 report<br>Food and water = 6 reports<br>No access = 13 reports
Colour of the burrow tube	13	23	Transparent colour = 1 report<br>Solid colour = 6 reports

			Tinted colour = 6 reports
Length of the burrow tube	45	80	Median = 32cm<br>Range = 12.5 – 45cm
Diameter of the burrow tube	45	80	Median = 10cm<br>Range = 5.1 – 20cm
Elevated height of the frontal open end of the burrow tube above the ground	37	66	Median = 6cm<br>Range = 0 – 7cm

S12

Table. A list of protocols that are referenced by the included studies.

Referenced protocol	Type of burrowing metric(s)	Cited No.
Deacon, 2006 [1]	Weight displaced	17
Andrews et al., 2012 [2]	Weight displaced	15
Jirkof et al., 2010 [3]	• Weight displaced<br>• Latency to burrow<br>• Duration of burrowing	7
Wodarski et al., 2016 [4]	Weight displaced	4
Rutten et al., 2014 [5]	Weight displaced	4
Jirkof et al., 2013 [6]	Weight displaced	3
Rutten et al., 2014 [7]	Weight displaced	3
Huang et al., 2013 [8]	• Weight displaced<br>• Latency to burrow	2
Das V et al., 2017 [9]	Weight displaced	1
Deacon, 2009 [10]	Weight displaced	1
Deacon, 2012 [11]	Weight displaced	1
Contet et al., 2001 [12]	Weight displaced	1
Rutten et al., 2018 [13]	Weight displaced	1
Deseure and Hans, 2018 [14]	Weight displaced	1
Jirkof et al., 2014 [15]	• Weight displaced<br>• Latency to burrow<br>• Duration of burrowing	1
Jirkof et al., 2012 [16]	Weight displaced	1
Bryden et al., 2015 [17]	Weight displaced	1
Gould et al., 2016 [18]	Weight displaced	1

1. Deacon RMJ. Burrowing in rodents: A sensitive method for detecting behavioral dysfunction. *Nat Protoc*. 2006;1(1):118-121. doi:10.1038/nprot.2006.19

2. Andrews N, Legg E, Lisak D, et al. Spontaneous burrowing behaviour in the rat is reduced by peripheral nerve injury or inflammation associated pain. *Eur J Pain*. 2012;16(4):485-495. doi:10.1016/j.ejpain.2011.07.012

3. Jirkof P, Cesarovic N, Rettich A, Nicholls F, Seifert B, Arras M. Burrowing behavior as an indicator of post-laparotomy pain in mice. *Front Behav Neurosci*. 2010;4:165. doi:10.3389/fnbeh.2010.00165

4. Wodarski R, Delaney A, Ultenius C, et al. Cross-centre replication of suppressed burrowing behaviour as an ethologically relevant pain outcome measure in the rat: a prospective multicentre study. *Pain*. 2016;157(10):2350-2365. doi:10.1097/j.pain.0000000000000657

5. Rutten K, Schiene K, Robens A, et al. Burrowing as a non-reflex behavioural readout for analgesic action In a rat model of sub-chronic knee joint inflammation. *Eur J Pain (United Kingdom)*. 2014;18(2):204-212. doi:10.1002/j.1532-2149.2013.00358.x

6. Jirkof P, Leucht K, Cesarovic N, et al. Burrowing is a sensitive behavioural assay for monitoring general wellbeing during dextran sulfate sodium colitis in laboratory mice. *Lab Anim*. 2013;47(4):274-283. doi:10.1177/0023677213493409

7. Rutten K, Robens A, Read SJ, Christoph T. Pharmacological validation of a refined burrowing paradigm for prediction of analgesic efficacy in a rat model of sub-chronic knee joint inflammation. *Eur J Pain (United Kingdom)*. 2014;18(2):213-222. doi:10.1002/j.1532-2149.2013.00359.x

8. Huang W, Calvo M, Karu K, et al. A clinically relevant rodent model of the HIV antiretroviral drug stavudine induced painful peripheral neuropathy. *Pain*. 2013;154(4):560-575. doi:10.1016/j.pain.2012.12.023

9. Das V, Kroin JS, Moric M, Buvanendran A. Biochemical and Pharmacological Characterization of a Mice Model of Complex Regional Pain Syndrome. *Reg Anesth Pain Med*. 42(4):507-516. doi:10.1097/AAP.0000000000000622

10. Deacon RMJ. Burrowing: A sensitive behavioural assay, tested in five species of laboratory rodents. *Behav Brain Res*. 2009;200(1):128-133. doi:10.1016/j.bbr.2009.01.007

11. Deacon R. Assessing burrowing, nest construction, and hoarding in mice. *J Vis Exp*. 2012;(59):1-10. doi:10.3791/2607

12. Contet C, Rawlins JNP, Deacon RMJ. A comparison of 129S2/SvHsd and C57BL/6JOlaHsd mice on a test battery assessing sensorimotor, affective and cognitive behaviours: Implications for the study of genetically modified mice. *Behav Brain Res*. 2001;124(1):33-46. doi:10.1016/S0166-4328(01)00231-5

13. Rutten K, Gould SA, Bryden L, Doods H, Christoph T, Pekcec A. Standard analgesics reverse burrowing deficits in a rat CCI model of neuropathic pain, but not in models of type 1 and type 2 diabetes-induced neuropathic pain. *Behav Brain Res*. 2018;350:129-138. doi:10.1016/j.bbr.2018.04.049

14. Deseure K, Hans G. Orofacial neuropathic pain reduces spontaneous burrowing behavior in rats. *Physiol Behav*. 2018;191:91-94. doi:10.1016/j.physbeh.2018.04.020

15. Jirkof P. Burrowing and nest building behavior as indicators of well-being in mice. *J Neurosci Methods*. 2014;234:139-146. doi:10.1016/j.jneumeth.2014.02.001

16. Jirkof P, Cesarovic N, Rettich A, Fleischmann T, Arras M. Individual housing of female mice: Influence on postsurgical behaviour and recovery. *Lab Anim*. 2012;46(4):325-334. doi:10.1258/la.2012.012027

17. Bryden LA, Nicholson JR, Doods H, Pekcec A. Deficits in spontaneous burrowing behavior in the rat bilateral monosodium iodoacetate model of osteoarthritis: an objective measure of pain-related behavior and analgesic efficacy. *Osteoarthr Cartil*. 2015;23(9):1605-1612. doi:10.1016/j.joca.2015.05.001

18. Gould SA, Doods H, Lamla T, Pekcec A. Pharmacological characterization of intraplantar Complete Freund's Adjuvant-induced burrowing deficits. *Behav Brain Res*. 2016;301:142-151. doi:10.1016/j.bbr.2015.12.019
